# Diverse Reactivity of Amidinate-Supported Boron Centers with the Hypersilyl Anion and Access to a Monomeric Secondary Boron Hydride

**DOI:** 10.1021/acs.inorgchem.4c00612

**Published:** 2024-04-23

**Authors:** Sanjukta Pahar, Yara van Ingen, Rasool Babaahmadi, Benson M. Kariuki, Thomas Wirth, Emma Richards, Rebecca L. Melen

**Affiliations:** † Cardiff Catalysis Institute, School of Chemistry, Cardiff University, Translational Research Hub, Maindy Road, Cathays, Cardiff CF24 4HQ, Cymru/Wales, U.K.; ‡ School of Chemistry, Cardiff University, Main Building, Park Place, Cardiff CF10 3AT, Cymru/Wales, U.K.

## Abstract

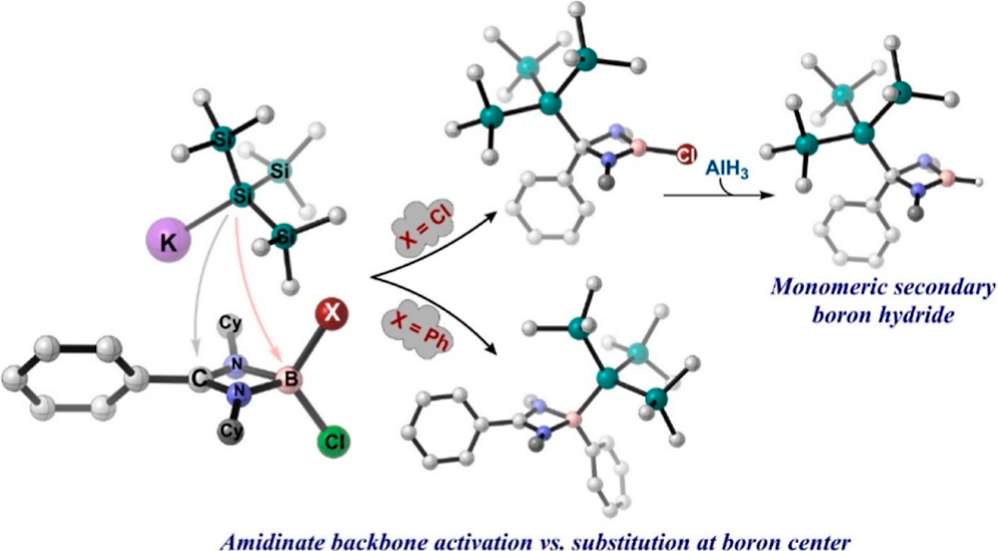

Diverse reactivity of the bulky tris(trimethylsilyl)silyl substituent [Si(SiMe_3_)_3_], also known as the hypersilyl group, was observed for amidinate-supported dichloro- and phenylchloroborane complexes. Treatment of the dichloroborane with potassium tris(trimethylsilyl)silyl led to the activation of the backbone β-carbon center and formation of saturated four-membered heterocyclic chloroboranes R′{Si(SiMe_3_)_3_}C(NR)_2_BCl [R′ = Ph, R = Cy (**3**); R′ = Ph, R = *i*Pr (**6**); R′ = *t*Bu, R = Cy (**8**)], whereas the four-membered amidinate hypersilyl-substituted phenyl borane **4** {PhC(NCy)_2_B(Ph)[Si(SiMe_3_)_3_]} was observed for the case of an amidinate-supported phenylchloroborane. The highly deshielded ^11^B NMR spectroscopic resonance and the distinct difference in the ^29^Si NMR spectrum confirmed the presence of a σ-donating hypersilyl effect on compounds **3**, **6**, and **8**. Reaction of **3** with the Lewis acid AlCl_3_ led to the formation of complex **11** in which an unusual cleavage of one of the C–N bonds of the amidinate backbone is observed. Nucleophilic substitution at the boron center of saturated chloroborane **3** with phenyllithium generated the phenylborane derivative **12**, whereas the secondary monomeric boron hydride **13** was observed after treatment with alane (AlH_3_). All compounds (**2**–**13**) have been fully characterized by NMR spectroscopy and single-crystal X-ray structure determination studies.

## Abstract 2

Diverse reactivity of strong σ-donating hypersilyl group on CN_2_B heterocycles of amidinate-supported dichloroboranes and phenylchloroborane afforded the unprecedented β-carbon activation of the amidinate ligand backbone with the hypersilyl group for dichloro-substituted amidinate–borane complexes, whereas the boron-substituted product has been observed for phenylchloroborane. A rare and thermally stable monomeric secondary borohydride was accomplished from the hypersilyl backbone-substituted saturated choloroborane, without adding any significant steric or electronic support to the compound.

## Introduction

**Scheme 1:**
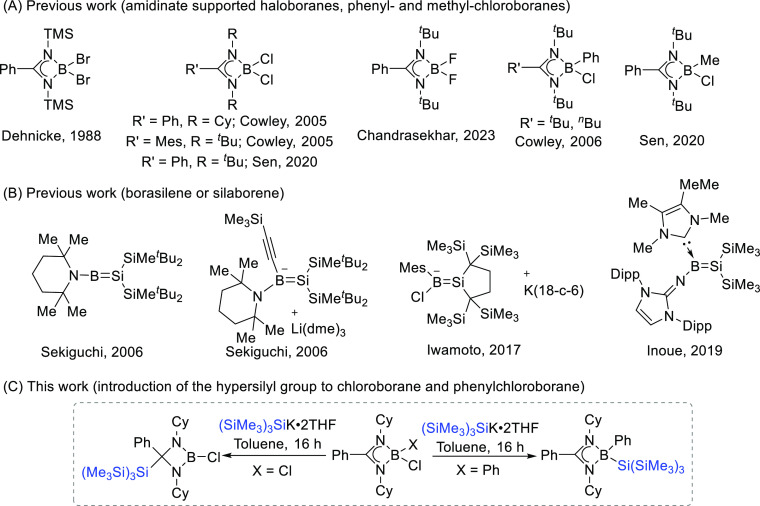
(A) Selected Examples of Amidinate-Supported Haloborane, Phenyl-, and Methyl-Chloro Boranes; (B) Reported Structures of Borasilene or Silaborene; (C) Diverse Reactivities of the Hypersilyl Group with Dichloro- and Phenylchloroborane Found in This Work

The early 21^st^ century witnessed enormous interest in the development of bidentate monoanionic nitrogen–donor amidinate ligands of the general formula [R′C(NR)_2_]^−^, as a popular replacement for the cyclopentadienyl ligand, as well as a close association with guanidinates as the ligand architecture.^1^ Among a plethora of ligands, amidinates have been designed in such a way that the steric and electronic effects can be readily modified by tailoring the C- and N-centered substituents to stabilize and tune the reactivity of various transition metals, lanthanides, and more recently, main group compounds. Following the first use with rare-earth metals in 1992 by Edelmann et al.,^2^ the application of amidinates rapidly expanded and fulfilled the quest for the preparation and stabilization of low-valent main group compounds^3−10^ together with applications in homogeneous catalysis^11−15^ and materials chemistry.^12^ Within the domain of *p*-block chemistry, group 13 and group 14 metal halide and metal–alkyl fragments have been well established using the amidinate ligand scaffold. For example, amidinate-supported silylenes have been reported for various small molecule activation and hydroboration reactions,^11,16−19^ and amidinate-supported alkyl aluminum cations have been shown as active catalysts for olefin polymerization.^20,21^ In addition, amidinate-based tetra-coordinated boron compounds have also been proven to act as photoluminescent materials^22^ and have been shown to activate gaseous CO and CO_2_, as well as carbonyl and nitrile functional groups.^18^ Surprisingly, a survey of known amidinate boron compounds reveals that the majority of amidinate boranes contain either halogen,^22,24,25^ Ph,^27,28^ or Me^29^ groups at the boron center (Scheme 1A). We hypothesize that alternative reactivity or catalytic activity of amidinate-supported boranes could be achieved by adding strong σ-donor ligands and effectively reducing the HOMO–LUMO gap.^26^ However, this would be more synthetically challenging. In 1981, Brook discovered the first well-defined organometallic compound containing a Si=C double bond by the photolysis of an acylsilane, using the hypersilyl group, [tris(trimethylsilyl)silyl], with main group compounds.^30^ More recently, substantial research has been reported on the use of the tris(trimethylsilyl)silyl group as a stabilizing group for novel main-group elements by the groups of Stalke,^31^ Aldridge,^32^ Inoue,^33,34^ Castel,^35,36^ Rivard,^37^ Leszczyńska,^38^ Sen,^3,17,39−41^ Marschner, Müller, Baumgartner, and others.^42−44^ The rapid growth of the use of the hypersilyl group in the synthesis and stabilization of several organometallic and main group compounds is a result of its pronounced steric effect, along with the strong σ-donor strength of the silyl ligand. The commercial readiness of the precursors and the easy access of further functionalization of the SiMe_3_ moieties makes the hypersilyl group advantageous with a delicate balance between strong σ-donation and kinetic protection. Although a few borasilene or silaborene compounds have been reported with other silyl precursors (Scheme 1B), there does not seem to be any literature on boranes containing the hypersilyl group until date.^45−47^ Herein, we investigate the different reactivities of the hypersilyl group on amidinate-supported dichloroboranes and phenylchloroborane by the treatment of the four-membered CN_2_B heterocycle with KSi(SiMe_3_)_3_ (Scheme 1C). The reactivity of the Si(SiMe_3_)_3_-substituted products is also explored.

## Results and Discussion

**Scheme 2:**
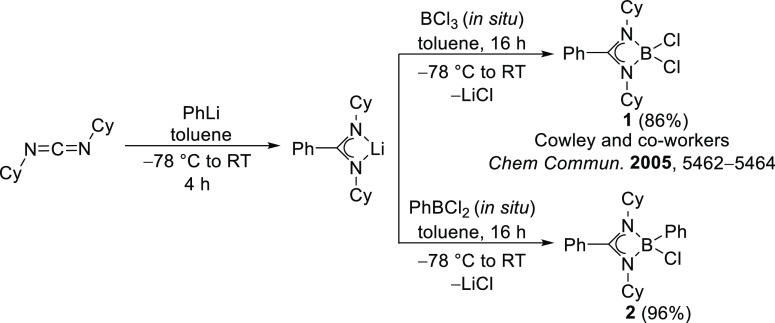
Synthesis of Dichloroborane (**1**) and Phenylchloroborane (**2**)

**Figure 1:**
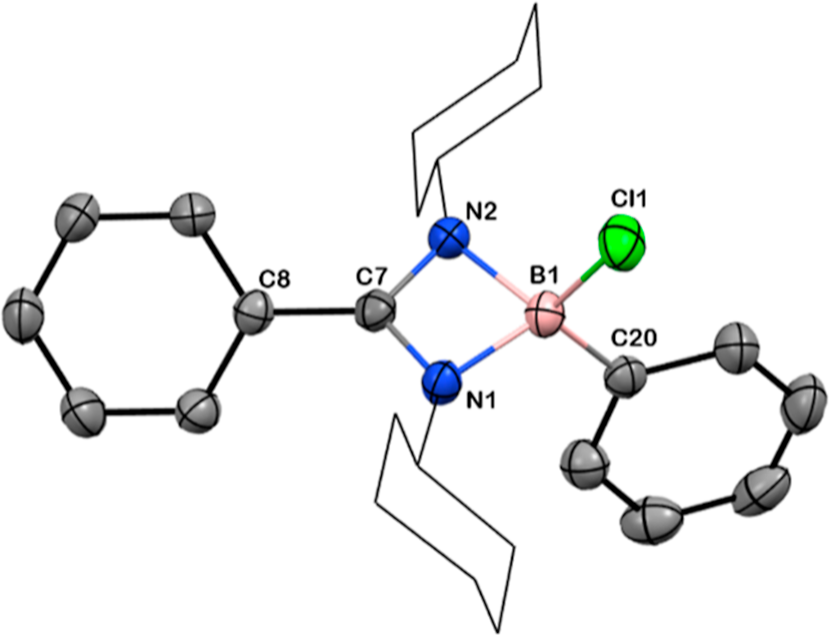
Solid-state structure of **2**. Anisotropic displacement parameters are drawn at 50% probability. B is shown in pink, C in gray, N in blue, and Cl in green. *Cy* groups have been drawn as wireframes, and H atoms are omitted for clarity. Selected bond lengths [Å] and angles [deg]: B1–Cl1 1.867(2), N1–B1 1.572(2), N2–B1 1.594(2), C7–N1 1.329(2), C7–N2 1.334(2), B1–C20 1.594(2), C7–C8 1.471(2); N1–C7–N2 101.4(1), N1–B1–N2 81.3(1), N2–B1–Cl1 111.7(1), N1–B1–C20 116.0(1), Cl1–B1–C20 113.0(1).

Initially, the dichloroborane precursor LBCl_2_ {L = [PhC(NCy)_2_]} was synthesized following literature procedures.^24^ We commenced our investigation by preparing amidinate-supported dichloroborane (**1**) and phenylchloroborane (**2**) by treating an equimolar amount of BCl_3_ or PhBCl_2_, respectively, with lithium *N*,*N*′-dicyclohexylamidinate [Ph(NCy)_2_Li] (Scheme 2). While compound **1** had been previously reported by Cowley and co-workers,^48^**2** could be extracted with toluene to afford a thermally stable colorless crystalline solid in 96% yield. Compound **2** crystallizes in monoclinic symmetry with the *P*2_1_/*c* space group. The B–Cl bond length 1.867(2) Å for **2** is comparatively longer than the reported bond length for compound **1** [1.832(3) Å] (Figure 1). The ^11^B NMR spectrum showed a peak at δ = 8.7 ppm for **2**, similar to that observed for dichloroborane precursor **1** (δ = 6.7 ppm).

**Scheme 3:**
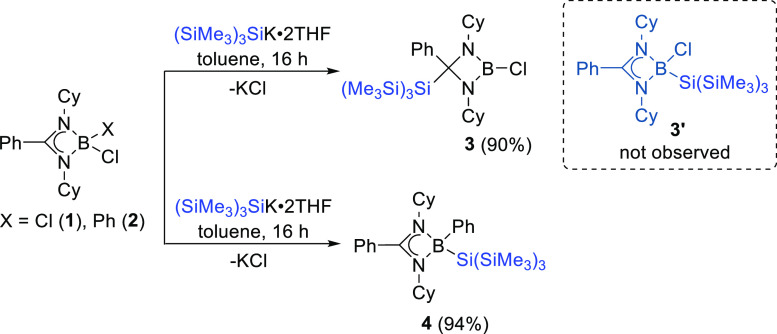
Diverse Reactivity of the Hypersilyl [tris(trimethylsilyl)silyl] Group with Compounds **1** and **2** and the Synthesis of Compounds **3** and **4**

**Figure 2:**
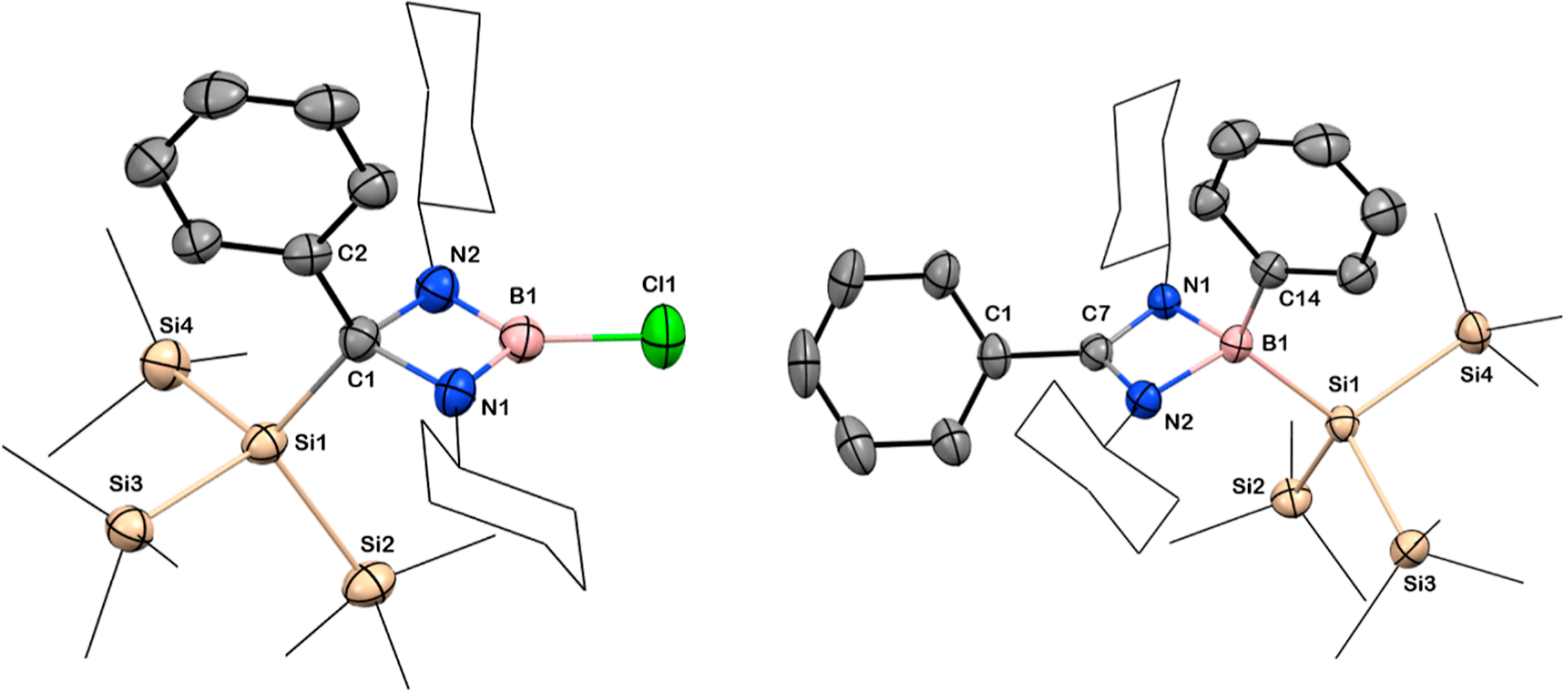
Solid-state structures of compounds **3** and **4**. Anisotropic displacement parameters are depicted at the 50% probability level. B is shown in pink, C in gray, N in blue, Cl in green, and Si in beige. *Cy* and Si*Me*_3_ groups have been drawn as wireframes, and H atoms are omitted for clarity. Selected bond lengths [Å] and angles [deg]: for **3**: B1–Cl1 1.772(3), C1–Si1 1.977(2), Si1–Si2 2.3763(9), B1–N1 1.403(3), B1–N2 1.408(3), C1–N1 1.497(3), C1–N2 1.509(3); N1–B1–N2 95.6(2), N1–C1–N2 87.7(2), N2–B1–Cl1 130.9(2), N1–B1–Cl1 133.5(2), N1–C1–Si1 112.6(1), C1–C1–Si1 117.8(2); for **4**: B1–C14 1.612(3), B1–Si1 2.102(2), Si1–Si2 2.3530(7), B1–N1 2.593(2), B1–N2 1.607(3), C1–C7 1.482(3); N1–B1–N2 80.3(1), N1–C7–N2 101.2(2), N2– B1–Si1 116.3(1), C14–B1–Si1 116.3(1).

In line with the significant growth of the use of the electropositive tris(trimethylsilyl)silyl group for the synthesis of numerous organometallic compounds,^49^ we treated compounds **1** and **2** with equimolar amounts of KSi(SiMe_3_)_3_ in toluene at room temperature. While **2** smoothly afforded the hypersilyl-substituted phenylborane derivative (**4**) in high yields (94%) from substitution at the boron atom via traditional salt metathesis; under similar reaction conditions, **1** led to the formation of compound **3** instead of the analogous hypersilyl-substituted chloroborane **3′** (Scheme 3). Here, substitution at the backbone β-carbon of the amidinate moiety occurred giving the product **3** in 90% yield. Concentrated toluene solutions of both **3** and **4** at low temperature (−20 °C) produced high quality colorless crystals within 3–4 days. Single-crystal X-ray diffraction studies confirmed the structures of both compounds. The single crystal of compound **3** undergoes a solid-state phase transition at 200 K from the high temperature form (290 K) to the low temperature form (120 K), and the transformation occurs in a single-crystal-to-single-crystal manner (discussed in Supporting Information). The molecular structure of **3** contains a B–Cl bond length of 1.772(3) Å (Figure 2), which is shorter than that of the previously reported dichloroborane precursor **1** [1.832(3) Å]. A slightly longer Si–C bond than their covalent radii was observed for **3** at 1.977(2) Å, and an elongated B–Si bond of 2.102(2) Å was observed for **4** (Figure 2). **3** and **4** were further characterized by multinuclear NMR spectroscopy. Although the ^1^H NMR spectroscopic resonance for the three SiMe_3_ groups for **3** and **4** were observed in the same range, at δ = 0.40 and 0.53 ppm, respectively, the distinguishable ^11^B resonances at δ = 21.8 and 9.1 ppm validated the different reactivity and coordination around the boron center of **3** and **4**, respectively. The ^29^Si NMR spectroscopic signals were detected at δ = −70.28 [*Si*(SiMe_3_)] and −13.58 [Si(*Si*Me_3_)] ppm with a large resonance gap between two Si centers for **3**, whereas for **4**, these were observed at δ = −10.77 [*Si*(SiMe_3_)] and −11.30 [Si(*Si*Me_3_)] ppm. The molecular ion peaks were detected with the highest relative intensity at *m*/*z* 577.3232 and 619.3937 for **3** and **4**, respectively. Interestingly, when we tried similar reactions with difluoro and dibromo boranes, we had no success in observing either the backbone-substituted or the hypersilyl-substituted borane products by either NMR spectroscopic studies or single-crystal X-ray diffraction.

**Figure 3:**
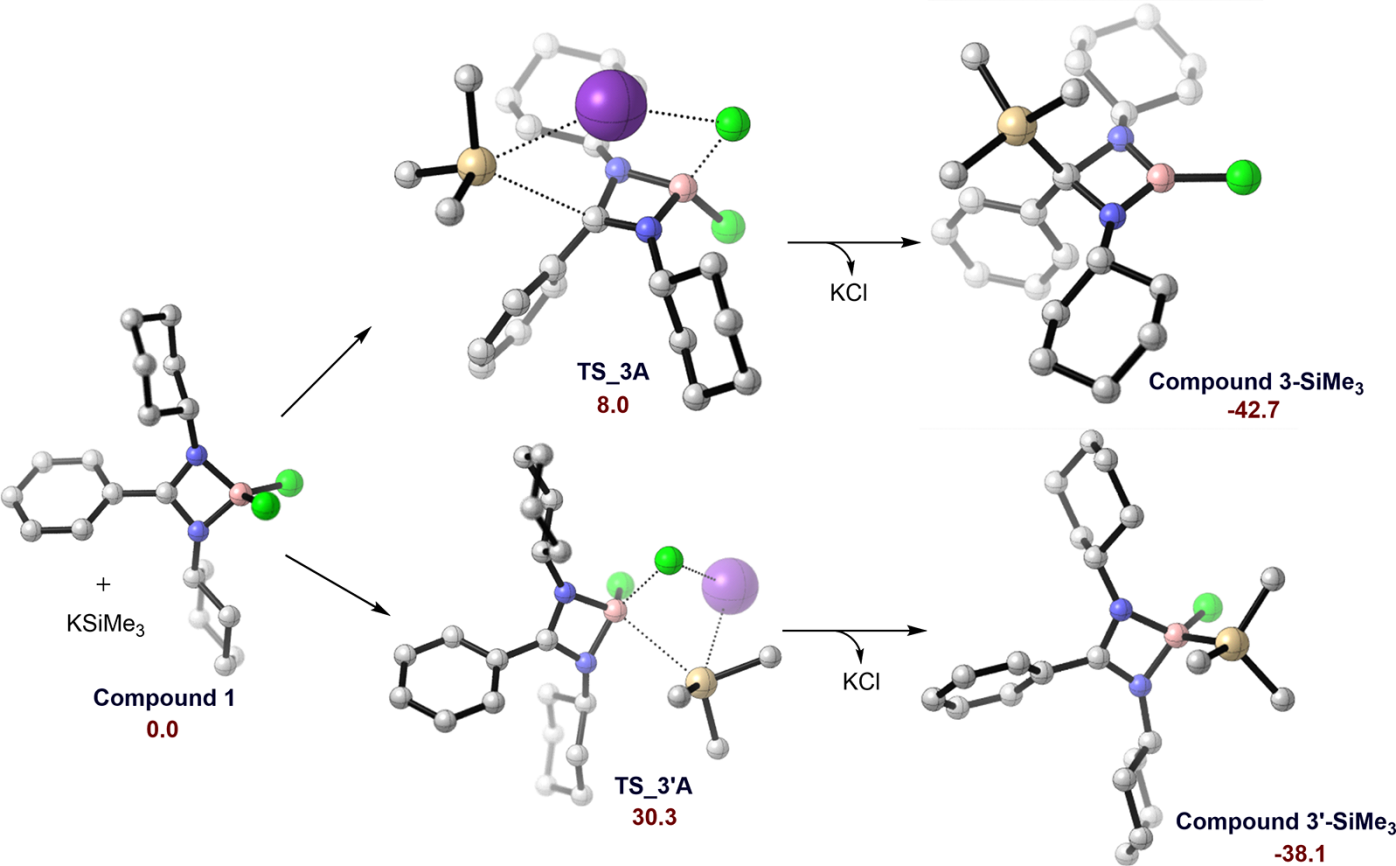
Energy profile for the formation of **3-SiMe_3_** versus **3′-SiMe_3_**. Relative energies are given in kcal/mol. B is shown in pink, C in gray, N in blue, Cl in green, K in purple, and Si in beige. Hydrogen atoms are omitted for clarity.

Encouraged by the clean and high-yielding formation of **3** over **3′**, we conducted density functional theory (DFT) studies to understand the thermodynamic and kinetic accessibility of the reaction at the SMD (toluene)/M06-2X-D3/def2-TZVP//BP86/6-31g(d) level of theory considering a less bulky silyl anion (–SiMe_3_) as **3**-**SiMe_3_** and **3′**-**SiMe_3_** (Figure 3). The DFT calculations reveal that the formation of **3**-**SiMe_3_** is favorable (−42.7 kcal/mol) with an activation barrier of 8.0 kcal/mol (**TS_3A**), whereas the reaction energy is much higher for the formation of **3′**-**SiMe_3_** (−38.1 kcal/mol), as well as its respective transition state **TS_3′A** (30.3 kcal/mol), which determines the formation of backbone β-carbon-substituted product **3**.

**Scheme 4:**
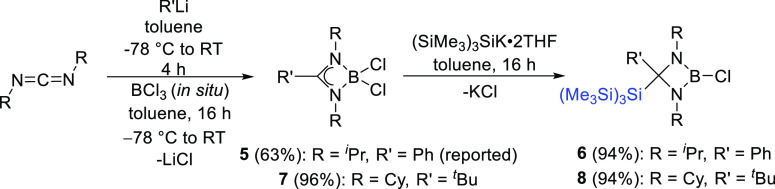
Synthesis of Dichloroboranes **5** (Reported) and **7** and Backbone-Substituted Hypersilyl Group Containing Choloroboranes **6** and **8**

**Figure 4:**
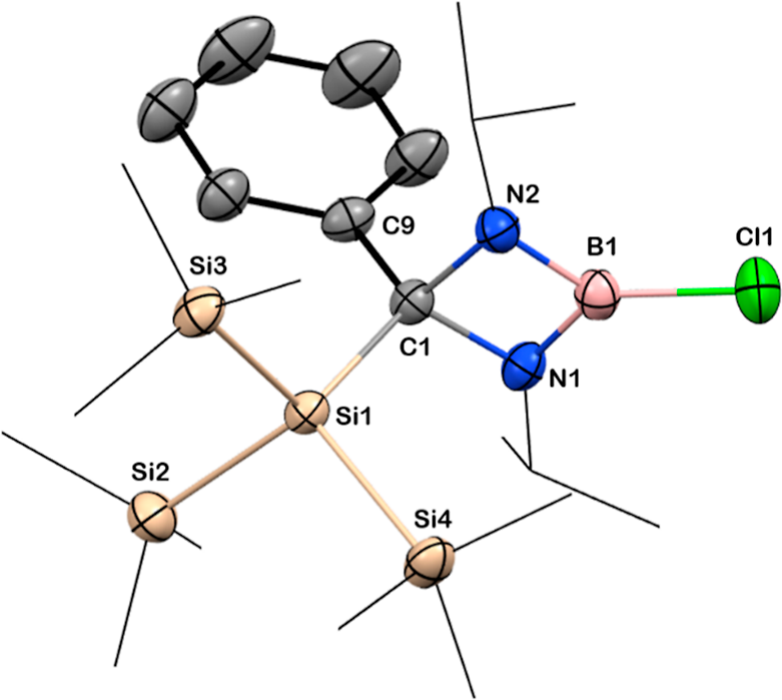
Solid-state structure of **6**. Anisotropic displacement parameters are depicted at the 50% probability level. B is shown in pink, C in gray, N in blue, Cl in green, and Si in beige. ^*i*^Pr and Si*Me*_3_ groups have been drawn as wireframes, and H atoms are omitted for clarity. Selected bond lengths [Å] and angles [deg]: B1–Cl1 1.768(2), C1–Si1 1.974(2), C1–N1 1.502(2), C1–N2 2.511(2), B1–N1 1.409(2), B1–N2 1.412(3), C1–C9 1.522(2); N1–B1–N2 95.1(1), N1–C1–N2 87.5(1), N1–C1–Si1 112.6(1), C9–C1–Si1 117.4(1), N2–B1–Cl1 132.2(2), N1–B1–Cl1 132.7(2).

To understand the preferred formation of **3** instead of **3′** and to investigate the effect of the substituents on the nitrogen center of the amidinate ligand, we prepared the previously reported dichloroborane **5**,^23^ containing an isopropyl group attached to the nitrogen center of the amidinate ligand, and performed the reaction with KSi(SiMe_3_)_3_ under similar reaction conditions (Scheme 4). As for dichloroborane **3**, an attack at the backbone of the amidinate occurred, and the formation of **6** was observed in 94% yield. The downfield shift in the ^11^B and ^29^Si NMR spectrum at δ = 22.1 ppm and δ = −69.73 [*Si*(SiMe_3_)] and −13.52 [Si(*Si*Me_3_)] ppm, respectively, further confirmed the formation of **6**, along with ^1^H NMR spectroscopic resonances at δ = 1.42 and 0.67 ppm for the 6H atoms of the isopropyl groups and at δ = 0.36 ppm for the 27H of the three Si*Me*_3_ groups. Single crystals of **6** were grown from a saturated toluene solution in a –20 °C freezer, and it crystallized in the triclinic space group *P*1̅ with B–Cl and C–Si bond lengths of 1.768(2) and 1.974(2) Å, respectively (Figure 4).

**Figure 5:**
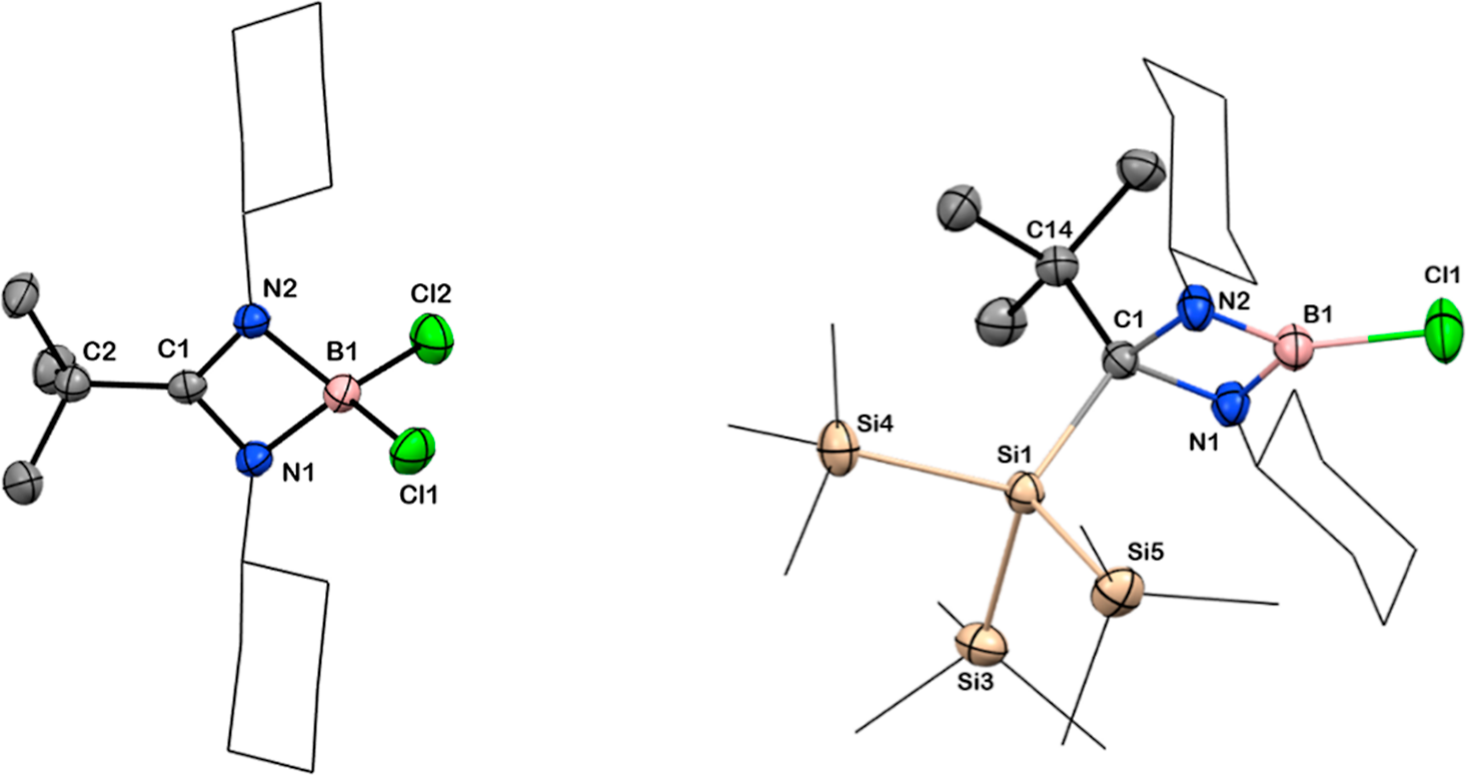
Solid-state structures of compounds **7** and **8**. Anisotropic displacement parameters are depicted at the 50% probability level. B is shown in pink, C in gray, N in blue, Cl in green, and Si in beige. *Cy* and Si*Me*_3_ groups have been drawn as wireframes, and H atoms are omitted for clarity. Selected bond lengths [Å] and angles [deg]: For **7**: B1–Cl1 1.834(2), B1–Cl2 1.844(2), N1–B1 1.551(2), N2–B1 1.558(3), C1–N1 1.342(2), C1–N2 1.342(2), C1–C2 1.510(3); N1–C1–N2 100.0(1), N1–B1–N2 82.7(1), N2–B1–Cl2 114.6(1), N1–B1–Cl1 115.9(1), Cl1–B1–Cl2 110.8(1); For **8**: B1–Cl1 1.767(2), C1–Si1 2.002(2), C1–N1 2.514(3), C1–N2 2.513(3), B1–N1 1.414(3), B1–N2 1.407(3), C1–C14 1.563(3); N1–B1–N2 95.6(2), N1–C1–N2 87.3(1), N1–C1–Si1 110.5(1), C14–C1–Si1 116.8(1).

In an attempt to prevent attack at the backbone β-carbon center of the amidinate ligand and to encourage substitution at the boron center, we introduced further steric hindrance into the ligand by changing the phenyl group attached to the carbon atom of the amidinate ligand with a more bulky *tert*-butyl group. Compound **7** was prepared following the same literature procedure^23^ for **5** using *tert*-butyl lithium instead of phenyllithium in the first step. **7** was then reacted with KSi(SiMe_3_)_3_ under similar reaction conditions to those described for the synthesis of **6**. Again, an attack occurred at carbon, yielding the analogous saturated chloroborane compound **8** in high yields (94%) (Scheme 4). Colorless single crystals of **7** and **8** suitable for X-ray diffraction studies were grown from saturated toluene solutions at −20 °C. The notable downfield ^11^B NMR spectroscopic shift of **8** at δ = 20.8 ppm compared to δ = 5.1 ppm of **7** again confirms the formation of **8** from the attack of the hypersilyl group at the backbone carbon of the amidinate scaffold. The ^1^H and ^29^Si NMR spectroscopic shifts for the three Si(Si*Me*_3_) groups show peaks at δ = 0.42 ppm (27H), δ = −71.62 [*Si*(SiMe_3_)], and −12.05 [Si(*Si*Me_3_)] ppm, respectively, and the molecular ion peak was detected at *m*/*z* 557.3547 for **8**. Compound **7** crystallized in the triclinic space group *P*1̅, whereas **8** crystallized in the monoclinic space group *P*2_1_/*c* (Figure 5). The B–Cl bond length [1.767(2) Å] is significantly shorter for compound **8** than for **7** [1.844(2) and 1.834(2) Å] similar to the amidinate-supported saturated chloroboranes discussed earlier (**3** and **6**).

**Scheme 5:**
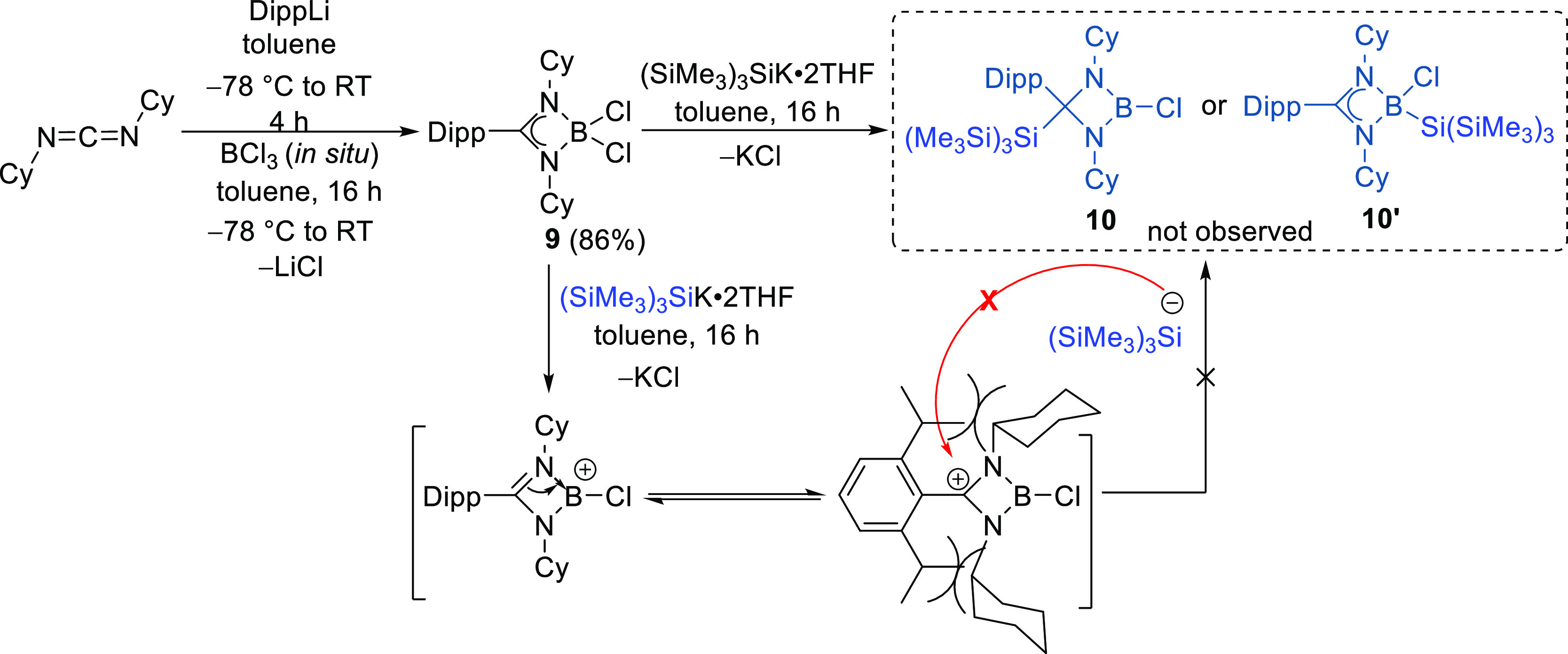
Synthesis of Dipp-Amidinate-Substituted Dichloroborane **9**

**Figure 6:**
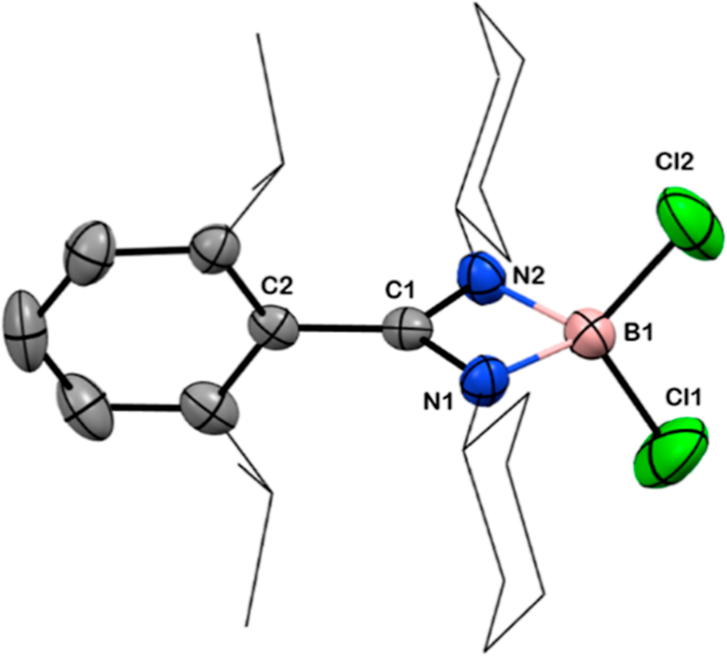
Solid-state structure of compound **9**. Anisotropic displacement parameters are depicted at the 50% probability level. B is shown in pink, C in gray, N in blue, Cl in green, and Si in beige. *Cy* and ^*i*^Pr groups have been drawn as wireframes, and H atoms are omitted for clarity. Selected bond lengths [Å] and angles [deg]: B1–Cl1 1.814(9), B1–Cl2 1.822(9), N1–B1 1.571(9), N2–B1 1.571(9), C1–N1 1.334(7), C1–N2 1.328(8), C1–C2 1.484(6); N1–C1–N2 101.5(5), N1–B1–N2 82.0(5), N2–B1–Cl2 114.7(5), N1–B1–Cl1 113.9(5), Cl1–B1–Cl2 112.4(5).

To introduce further steric hindrance at the backbone carbon, we synthesized the Dipp (Dipp = 2,6-diisopropylphenyl) amidinate-substituted dichloroborane [DippC(NCy)_2_BCl_2_] (**9**, 86% yield) and treated it with KSi(SiMe_3_)_3_ following the same reaction procedure. Despite a distinct color change in the reaction from pale yellow to red, neither the backbone-substituted nor the hypersilyl-substituted boron product was observed (Scheme 5). A saturated toluene solution of **9** formed suitable single crystals for X-ray diffraction studies and proved the desired steric protection with proximal alignment of the hydrogens from the isopropyl groups of the Dipp substituents and cyclohexyl groups of the amidinate ligand framework in compound **9** (molecular structure with H atoms shown in Figure S48 in the Supporting Information) to prevent the backbone attack by the hypersilyl group (Figure 6).

**Scheme 6:**
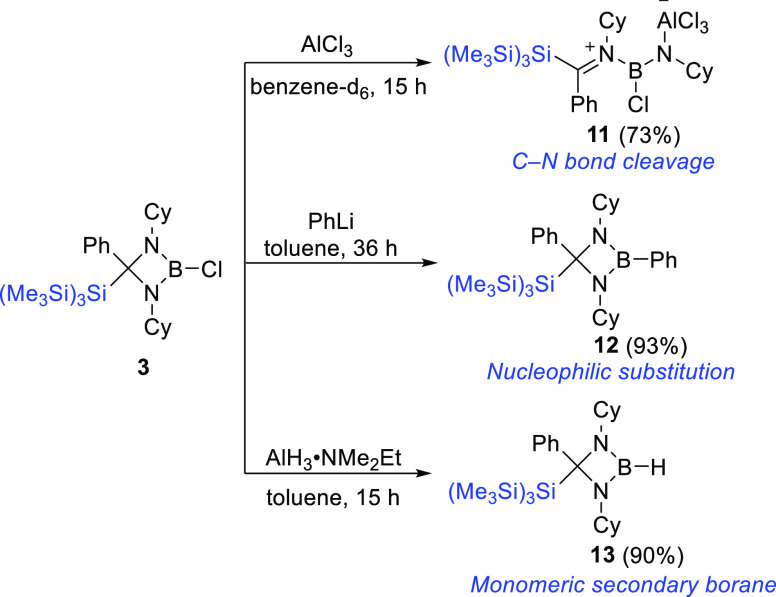
Reactivity of Chloroborane **3** and Synthesis of **11**–**13**

**Figure 7:**
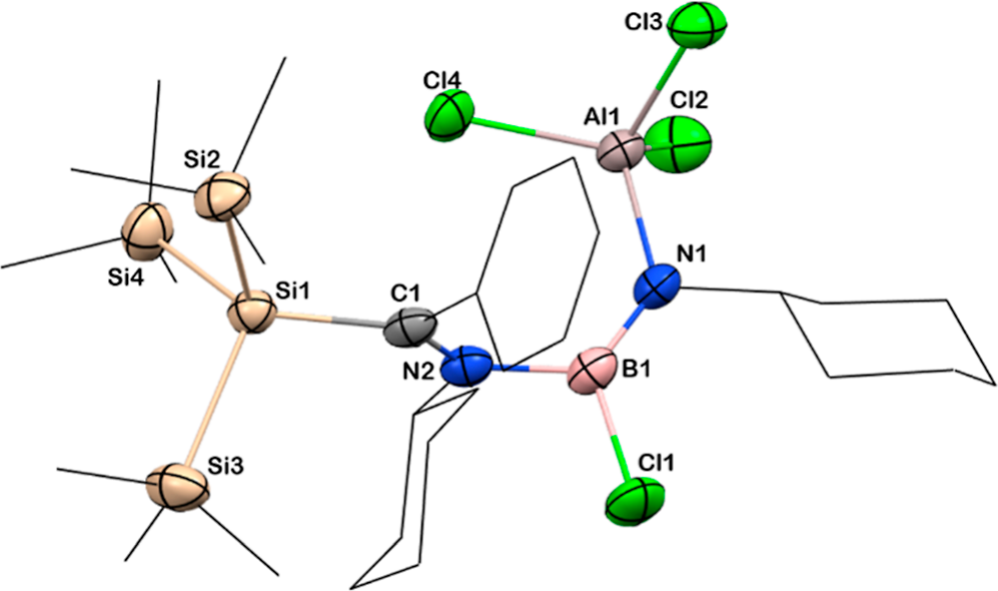
Solid-state structure of compound **11**. Anisotropic displacement parameters are depicted at the 50% probability level. B is shown in pink, C in gray, N in blue, Cl in green, Si in beige, and Al in rosy brown. *Cy* and Si*Me*_3_ groups have been drawn as wireframes, and H atoms are omitted for clarity. Selected bond lengths [Å] and angles [deg]: B1–Cl1 1.785(2), C1–Si1 1.962(2), N1–Al1 1.868(1), B1–N1 1.361(3), B1–N2 1.546(2), C1–N2 1.296(3), Al1–Cl2 2.1364(9); N1–B1–N2 122.7(2), N2–C1–Si1 130.2(1), B1–N1–Al1 130.4(1).

The desire to form a stable boron compound with unusual structure, bonding, and reactivity has led to the synthesis of several low-valent and low coordinated boron compounds^50^ and has challenged the predominance of transition metals in catalysis,^51^ small molecule and bond activation,^52,53^ and has furthered the development of functional materials.^54^ Low-coordinate cationic boron centers are attractive and reactive Lewis acidic entities. Nöth and co-workers introduced the nomenclature of boronium, borenium, and borinium cations for the four-, three-, and two-coordinate boron-centered complexes, respectively.^55^ These highly Lewis acidic boron complexes are important in the field of bond activation and catalysis.^56^ Subsequently, we attempted to prepare the corresponding boron cations from complexes **2** and **3** by abstracting the chloride ligand. The treatment of **2** and **3** with chloride abstracting agents such as AgBF_4_, AgSbF_6_, AgNO_*x*_ (*X* = 2 or 3), AgOTf, GaCl_3_, and K[B(C_6_F_5_)_4_] failed to abstract the chloride, even in the presence of carbenes, phosphines, DMAP (4-dimethylaminopyridine), and other Lewis bases that can stabilize the resulting boron cations.^57−59^ However, the addition of 1 equiv of AlCl_3_ to a benzene solution of **3** in the absence of a Lewis base led to the cleavage of the C–N bond of the amidinate backbone and binding of N_amidinate_ to AlCl_3_, leading to zwitterionic **11** in 73% yield (Scheme 6). The reaction is expected to be driven by the formation of a tetra-coordinated anionic aluminum fragment through N → Al bond formation. A possible reaction mechanism is proposed in the Supporting Information (Scheme S1) for the formation of **11**. Colorless single crystals of **11** suitable for X-ray diffraction studies were grown from the saturated benzene solution at −4 °C in a freezer; the molecular structure of **11** validates our spectroscopic evidence. Compound **11** crystallizes in the triclinic space group, *P*1̅. The B–Cl bond length of **11** is 1.785(2) Å, slightly longer than the saturated chloroborane **3** [1.772(3) Å], and the new N_amidinate_–Al bond is observed at a distance of 1.868(1) Å (Figure 7). The aluminum center adopts a tetrahedral geometry with bond angles of 109.04(6), 108.99(5), 107.3(1), and 114.76(6)°. The only partial solubility of adduct **11** in most organic solvents did not allow us to record ^13^C, ^29^Si, or ^27^Al NMR spectra even after several attempts, as with time some decomposed solid compounds precipitated out. Gradual decomposition of **11** was also observed in ^1^H NMR spectroscopy with the appearance of undesired peaks (Figure S26 in the Supporting Information), whereas the ^11^B NMR spectrum showed a downfield resonance at δ = 28.7 ppm due to the cationic charge distribution over the fragment.

**Figure 8:**
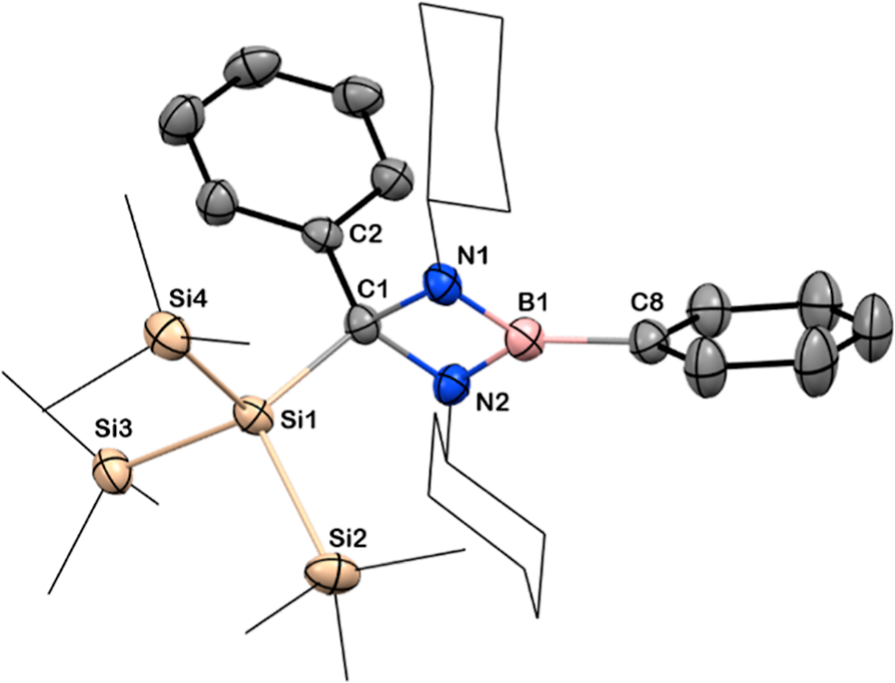
Solid-state structure of **12**. Anisotropic displacement parameters are depicted at the 50% probability level. B is shown in pink, C in gray, N in blue, and Si in beige. *Cy* and Si*Me*_3_ groups have been drawn as wireframes, and H atoms are omitted for clarity. Selected bond lengths [Å] and angles [deg]: B1–C8 1.570(3), B1–N1 1.430(3), B1–N21 1.423(3), N1–C1 1.504(3), N2–C1 1.491(2), C1–Si1 1.974(2), C1–C2 1.530(2); N1–B1–N2 92.7(2), N1–C1–N2 87.2(1), C2–C1–Si1 116.5(1), N1–B1–C8 134.6(2), N2–B1–C8 132.5(2).

Cleavage of the stable B–Cl bond of saturated chloroborane **3** was unsuccessful under harsh reaction conditions, such as using Li-naphthalide or KC_8_ as a reducing agent, except in the presence of phenyllithium. The toluene solution of **3** produced nucleophilic-substituted product **12** when treated with phenyllithium, exhibiting a B–C_Ph_ bond, with removal of LiCl over a prolonged reaction time more than 36 h (Scheme 6). Compound **12** crystallizes in triclinic symmetry with a *P*1̅ space group. The boron center adopts a trigonal-planar geometry (Figure 8) with a B–C_Ph_ bond length of 1.570(3) Å, which is slightly shorter than the phenyl-substituted tetra-coordinated B–C_Ph_ bond [1.612(3) Å] of compound **4**. The ^1^H NMR spectrum displays a singlet at δ = 0.46 ppm for the three Si(Si*Me*_3_) groups, and the ^29^Si NMR spectrum exhibits resonances at δ = −73.29 [*Si*(SiMe_3_)] and −13.42 [Si(*Si*Me_3_)] ppm for both the Si centers, while the ^11^B NMR spectroscopic signal is observed at δ = 32.8 ppm.

**Figure 9:**
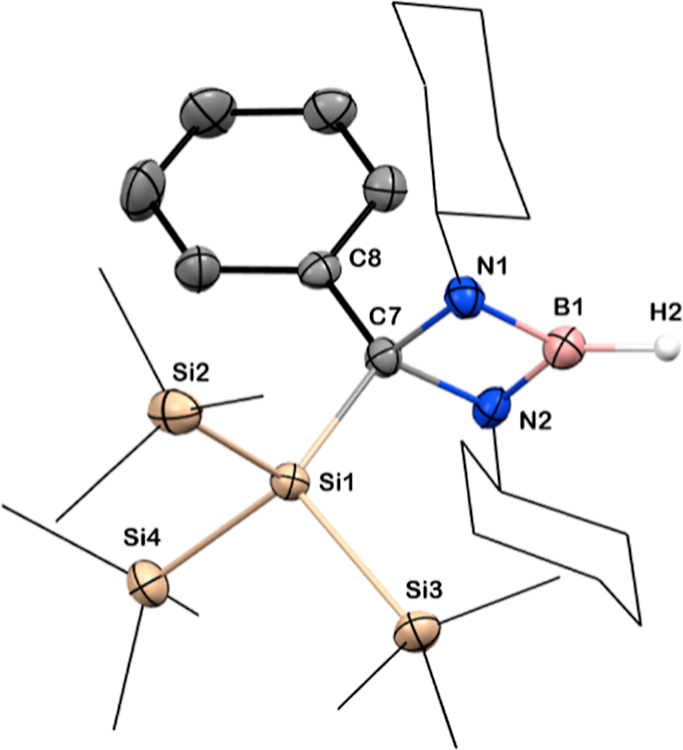
Solid-state structure of **13**. Anisotropic displacement parameters are depicted at the 50% probability level. Hydrogen atoms (except B–H) are omitted for clarity. B is shown in pink, C in gray, N in blue, Si in beige, H in white. *Cy* and Si*Me*_3_ groups have been drawn as wireframes for clarity. Selected bond lengths [Å] and angles [deg]: B1–N1 1.432(6), B1–N21 1.422(7), N1–C7 1.501(6), N2–C7 1.498(5), C7–Si1 1.967(3), C7–C8 1.527(6); N1–B1–N2 92.9(4), N1–C7–N2 87.2(3), C8–C7–Si1 118.5(3).

Boranes containing at least one B–H bond have appeared as powerful synthetic tools for hydroboration reactions. Among the many hydroborating borane catalysts, secondary boranes are very effective for the transformation, moreover, Piers’ borane [HB(C_6_F_5_)_2_] has emerged as a highly effective catalyst for hydroboration and acts as an efficient reagent in various other organic transformations due to its high Lewis acidity.^60^ Electrophilic secondary boranes are typically preferred and stable as monomer–dimer equilibria in solution,^61,62^ and isolable monomeric secondary boranes are extremely rare without sufficient steric provision.^63^ Recently, the research group of Martin reported a significantly high Lewis acidic monomeric secondary borane [tris(*ortho*-carboranyl)borane] containing two electron-withdrawing ortho carborane substituents and further utilized it as a potent hydroborating reagent.^64^ Inspired by Martin’s work, the addition of alane (AlH_3_·NMe_2_Et) to a toluene solution of **3** smoothly afforded the stable boron hydride **13** in 90% yield (Scheme 6). Surprisingly, compound **13** is monomeric in both solid and solution state with the absence of any steric protection, which was confirmed by NMR spectroscopy, mass spectrometry, and infrared spectroscopy. Variable temperature ^1^H NMR spectroscopic studies of a toluene-d_8_ solution of **13** displayed a resonance of the B–*H* at δ = 4.62 ppm (Figures S33 and S34 in the Supporting Information) along with a singlet for the Si(Si*Me*_3_)_3_ group, even at −60 °C. The ^11^B NMR spectroscopic resonance was observed at δ = 26.61 ppm for the tricoordinated boron center. The distinguishable IR stretching frequency with one sharp band at 2557 cm^–1^ confirmed the monomeric nature of *B*–*H* bond (Figure S38 in the Supporting Information), which is in good agreement with the monomeric secondary boron hydride reported by Martin and co-workers.^64^ Thermal characterization by differential scanning calorimetry (DSC) shows an endothermic melting event at 150.4 °C (Figure S40 in the Supporting Information), corroborating the analogous melting point measurement (∼162–164 °C). In addition, **13** shows thermal stability up to 250 °C, and no exothermic crystallization point could be observed in the cooling curve, suggesting that the crystallization of the sample takes place at temperatures below 30 °C (Figure S39 in the Supporting Information). Colorless crystals of **13** were grown from cooling a concentrated toluene solution. **13** crystallizes in the triclinic space group *P*1̅ adopting a distorted trigonal planar geometry (Figure 9). The hydride was detected in the difference Fourier map and refined isotropically. Due to the stability of the hydride compound, the traditional hydroboration reaction using **13** as an effective catalyst did not provide any significant results compared to the reported Piers’ borane [HB(C_6_F_5_)_2_] and/or tris(*ortho*-carboranyl)borane. Thus, we are currently exploring other organic transformations using monomeric boron hydride as well as catalytic studies with compounds **4** and **13**, which will be published in the future.

## Conclusions

The chemistry of the amidinate ligand in main-group chemistry is in its infancy compared to other ligand scaffolds. In this work, we have shown a unique β-carbon activation of the amidinate ligand with a hypersilyl group for dichloro-substituted amidinate–borane complexes with various electronic and steric C- and N-substituents (**3**, **6**, and **8**). The treatment with amidinate-supported phenylborane finally produced the expected hypersilyl-substituted phenylborane derivative (**4**) in high yields. Further attempts to cleave the exceptionally stable B–Cl bond of **3** led to the formation of ionic compound **11** upon cleavage of the ligand backbone and nucleophilic-substituted phenylborane **12** in the presence of AlCl_3_ and PhLi, respectively. Moreover, the formation of a rare and thermally stable monomeric secondary borohydride **13** was achieved from compound **3** from the reaction with alane, without significant steric or electronic support to compound **13**.

## Data Availability

Information about the data that underpins the results presented in this article can be found in the Cardiff University data catalogue at 10.17035/d.2024.0315834338.

## Supporting Information Available

General experimental information, structural description of **2**–**13**, details of synthesis, representative NMR spectra, thermal characterization data for compound **13**, tentative mechanism for the formation of compound **11**, crystallographic data for the structural analysis of compounds **2**–**13**, crystal structure of compounds **2**–**13** with thermal ellipsoids, solid-state phase transformation details of compound **3**, and computational details (PDF)
