# Supplementary material for: Diverse Reactivity of Amidinate-Supported Boron Centers with the Hypersilyl Anion and Access to a Monomeric Secondary Boron Hydride
Source: Inorg Chem. 2024 Apr 23;63(18):8302–11. doi: 10.1021/acs.inorgchem.4c00612 (PMC11080068; doi:10.1021/acs.inorgchem.4c00612)
Supplement: Supplementary file 1 — ic4c00612_si_001.pdf [file ic4c00612_si_001.pdf]

Supporting Information

for

Diverse Reactivity of Amidinate-Supported Boron Centers with the Hypersilyl Anion and Access to A Monomeric Secondary Boron Hydride

Sanjukta Pahar,[a] Yara van Ingen,[a] Rasool Babaahmadi,[a] Benson M. Kariuki,[b] Thomas Wirth,[b] Emma Richards,*[a,b] and Rebecca L. Melen*[a]

[a]*Cardiff Catalysis Institute, School of Chemistry, Cardiff University, Translational Research Hub, Maindy Road, Cathays, Cardiff, CF24 4HQ, Cymru/Wales, United Kingdom.*

[b]*School of Chemistry, Cardiff University, Main Building, Park Place, Cardiff, CF10 3AT, Cymru/Wales, United Kingdom.*

Authors to whom correspondence should be addressed:

E-mail: RichardsE10@cardiff.ac.uk

E-mail: MelenR@cardiff.ac.uk

Table of Contents

1. General experimental information.
2. Synthesis and characterization of compounds **2**–**4**, **6**–**9**, **11**–**13**.
3. Thermal characterization data for compound **13**.
4. Tentative mechanism for the formation of compound **11**.
5. Crystallographic data for the structural analysis of compounds **2**–**4**, **6**–**9**, **11**–**13**.
6. Solid-state phase transformation details of compound **3**.
7. Computational Details.
8. References.

1. General experimental information.

All reactions and manipulations including NMR spectroscopic measurements were carried out under an atmosphere of dry, O_2-free nitrogen using standard double-manifold techniques with a rotary oil pump applying standard Schlenk techniques. A nitrogen-filled glove box (MBraun) was used for product isolation and sample preparation for analysis. All solvents were dried by employing a Grubbs-type column system (Innovative Technology) or a solvent purification system MB SPS-800 and stored under a nitrogen atmosphere. Deuterated solvents were distilled and/or dried over molecular sieves before use. Chemicals were purchased from commercial suppliers and used as received without further purification. Compounds **1**, **5** and $\{(SiMe_3)_3Si\}K\cdot 2THF$ were prepared according to the literature procedure.[S1] ^{1}H, ^{13}C, ^{11}B, ^{27}Al, and ^{29}Si NMR spectra were recorded on Bruker Avance II 400 or Bruker Avance 500 spectrometer. Chemical shifts are expressed as parts per million (ppm, δ) downfield of tetramethylsilane (TMS) and are referenced to C_6D_6 (7.16/128.06 ppm) or $CDCl_3$ (7.26/77.16 ppm) as internal standard. The description of signals includes; s = singlet, d = doublet, t = triplet, q = quartet, and m = multiplet, br. = broad. All coupling constants are absolute values and are expressed in Hertz (Hz). Multinuclear NMR spectra were measured as ^{1}H decoupled. Yields are given as isolated yields. Mass spectra were measured on a Waters LCT Premier/XE or a Waters GCT Premier spectrometer. The molecular ion peak values are quoted for molecular ion plus hydrogen $[M+H]^+$. Thermal characterization data was measured using a double furnace PerkinElmer DSC 8000 coupled with PerkinElmer Intracooler II for temperature control.

2. Synthesis and characterization of compounds 2–4, 6–9, 11–13.

Synthesis of 2. PhLi (2.8 mL, 5.33 mmol, 1.9 M in diethyl ether) was added dropwise to a solution of *Cy*N=C=N*Cy* (1.0 g, 4.85 mmol) in toluene (12 mL) in a 100 mL Schlenk flask at –30 °C. The solution was warmed to ambient temperature and stirred for 4 h. Then, $PhBCl_2$ (5.09 mL, 5.09 mmol, 1.0 M in hexane) was added dropwise to the reaction mixture at –30 °C. The reaction mixture was warmed to room temperature and stirred for 16 h. The white precipitate of the reaction mixture was filtered through Celite. The resultant toluene filtrate was removed under reduced pressure and concentrated to 10–12 mL, stored at –20 °C in a freezer, which afforded colorless crystals of **2** suitable for single crystal X-ray diffraction analysis within one day. Yield: 1.9 g (96%). Mp: 182–184 °C.

2: ^{1}H NMR (400 MHz, C_6D_6, 25 °C): δ 8.22 (dd, J = 8.0, 1.3 Hz, 2H), 7.48–7.40 (m, 2H), 7.35–7.28 (m, 1H), 7.24–7.18 (m, 2H), 7.07–6.98 (m, 3H), 3.40–3.28 (m, 2H), 1.97 (d, J = 13.1 Hz, 2H), 1.84 (ddd, J = 25.5, 12.6, 3.7 Hz, 2H), 1.59 (d, J = 11.1 Hz, 2H), 1.50–1.44 (m, 2H), 1.29 (d, J = 9.4 Hz, 4H), 1.21 (s, 2H), 0.75 (dd, J = 14.9, 5.7 Hz, 4H) ppm. ^{13}C{^{1}H} NMR (101 MHz, C_6D_6, 25 °C): δ 171.5 (s), 133.3 (s), 131.2 (s), 129.2 (s), 127.9 (s), 127.4 (s), 54.6 (s), 34.1 (s), 33.9 (s), 25.6 (d, J = 6.3 Hz), 25.3 (s) ppm. ^{11}B{^{1}H} NMR (128 MHz, C_6D_6, 25 °C): δ 8.7 (s, 1B, *B*(Ph)Cl). ESI-HRMS [ligand] Calcd for $[\text{ligand+H}]^+$ $C_{19}H_{29}N_2$: 285.2331, found: 285.2331. Elemental Analysis (%) Calculated for $C_{34}H_{59}BN_2Si_4$: C = 73.81; H = 7.93; N = 6.89. Found 1: C = 73.93; H = 7.70; N = 6.34. Found 2: C = 73.99; H = 7.82; N = 6.38.

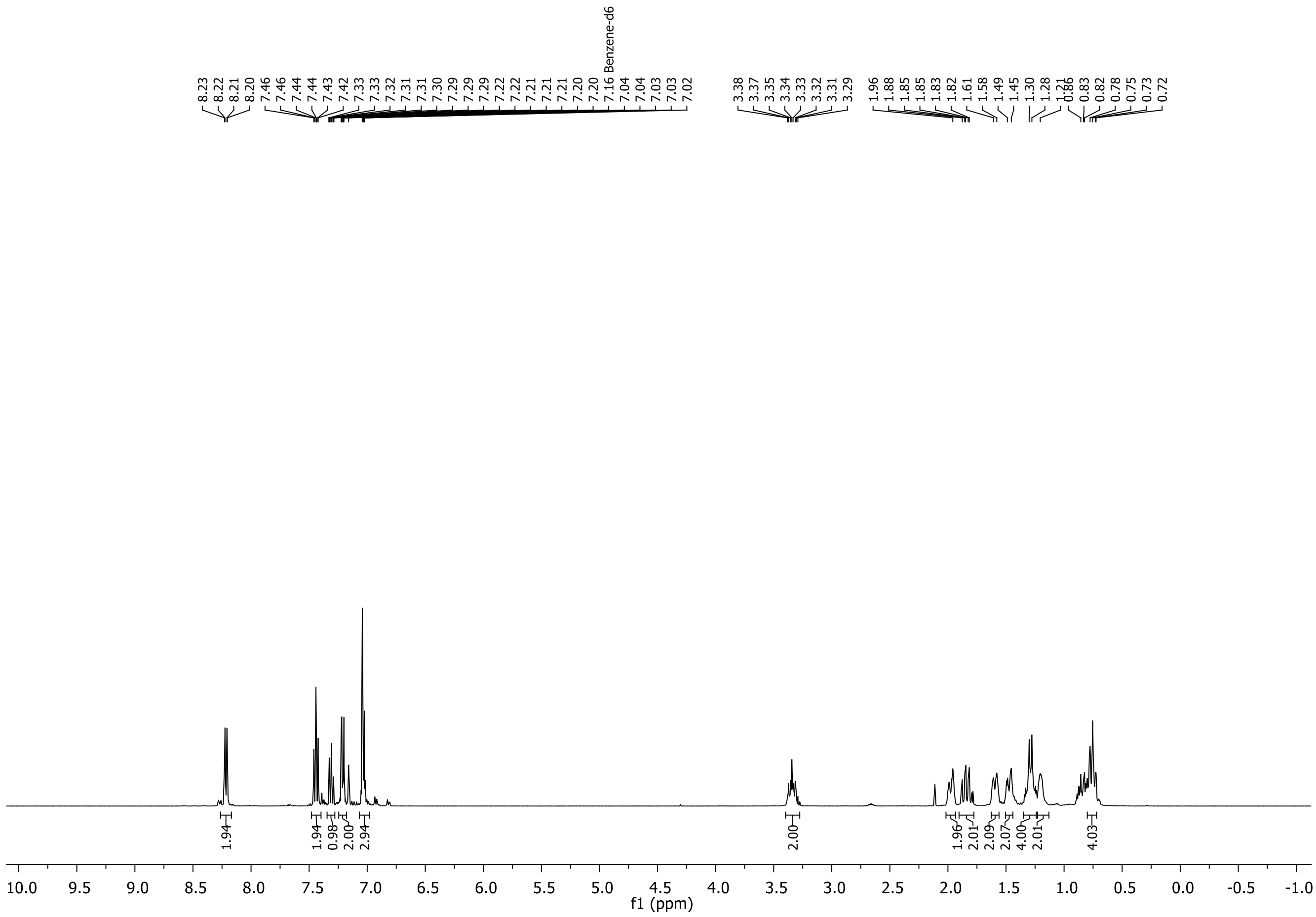

Figure S1. ^{1}H NMR spectrum of **2** (400 MHz, C_6D_6, 25 °C)

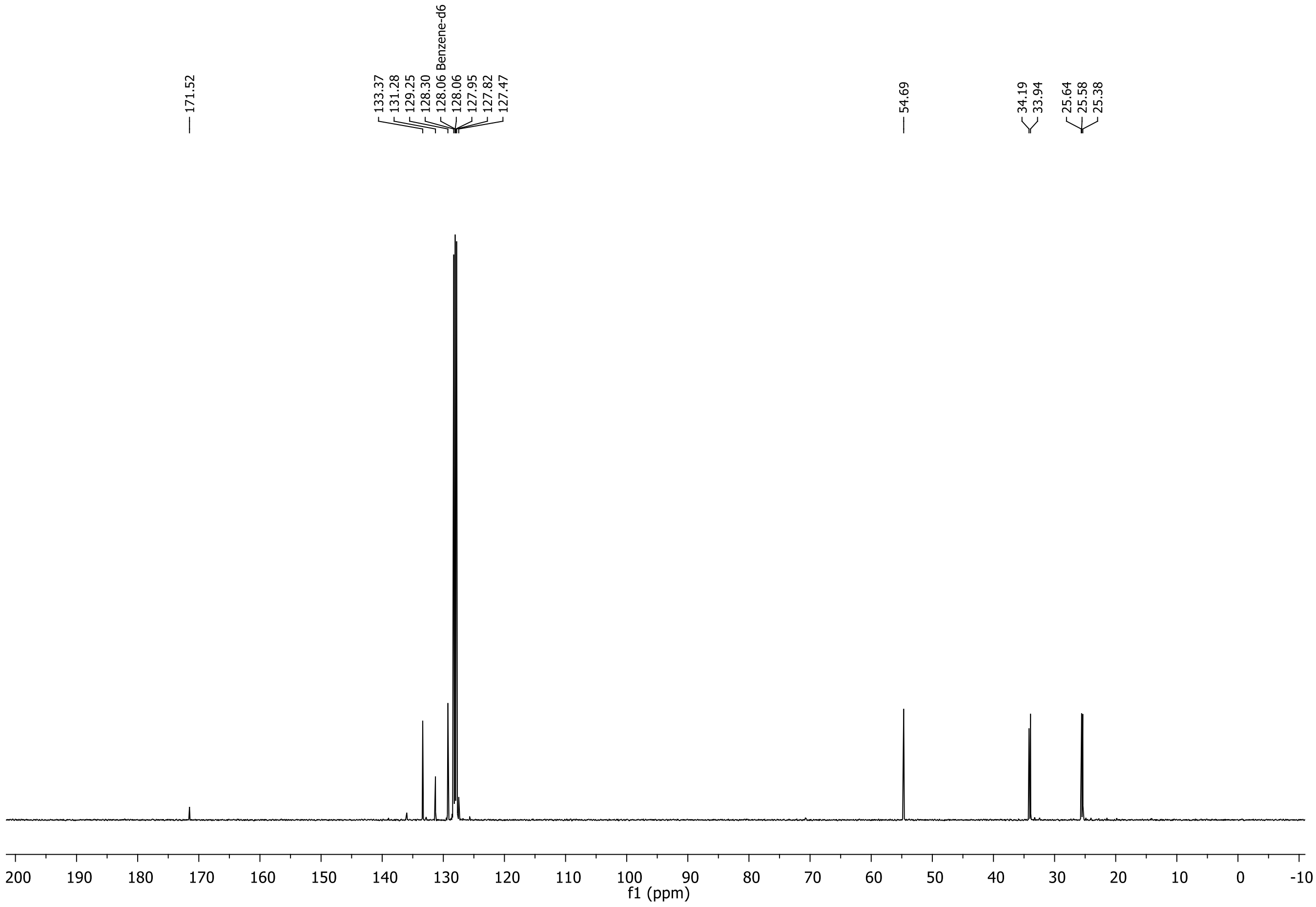

Figure S2. ^{13}C NMR spectrum of **2** (101 MHz, C_6D_6, 25 °C)

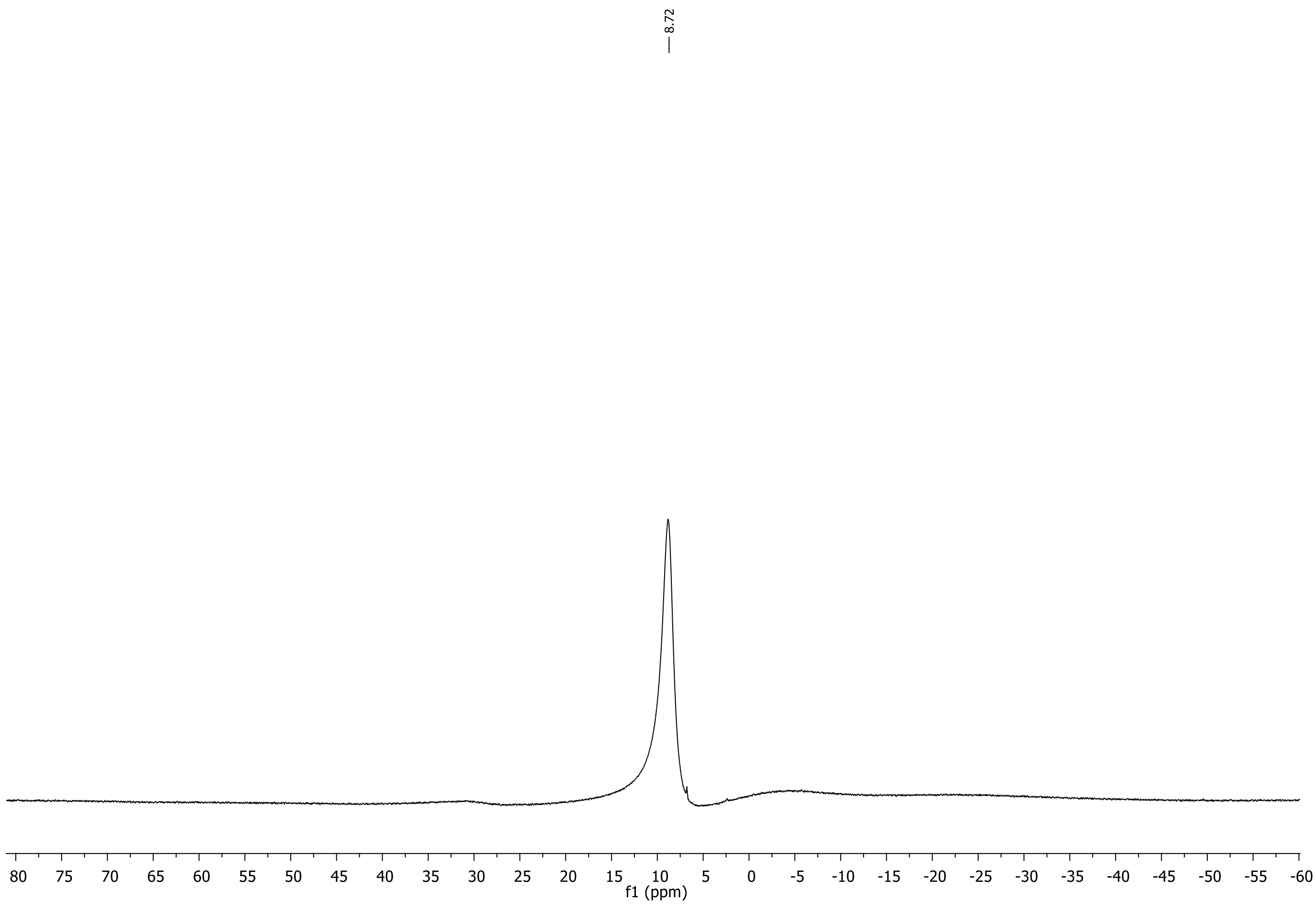

Figure S3. ^{11}B NMR spectrum of **2** (128 MHz, C_6D_6, 25 °C)

Synthesis of 3. A solution of potassium tris(trimethylsilyl)silane (372 mg, 0.865 mmol) in toluene (10 mL) was added to a solution of compound **1** (300 mg, 0.824 mmol) in toluene (8 mL) at –30 °C over a period of 15 min, and a slow color change from pale yellow to deep red was observed. Then, the reaction mixture was allowed to warm to room temperature and further stirred for 12 h. Subsequently, all volatiles were removed in vacuo and the residue was extracted into toluene (15 mL). The resultant toluene solution was concentrated to 5–8 mL and stored at –20 °C in a freezer, which afforded colorless crystals of **3** suitable for single crystal X-ray diffraction analysis within two days. Yield: 425 mg (90%). Mp: 198–200 °C.

3: ^{1}H NMR (400 MHz, C_6D_6, 25 °C): δ 7.74–7.69 (m, 2H), 7.10–7.05 (m, 2H), 7.04–6.99 (m, 1H), 3.08 (tt, J = 11.2, 3.3, 2H), 2.16 (d, J = 13.1, 2H), 1.98 (ddd, J = 16.7, 14.2, 3.6, 2H), 1.72 (d, J = 12.7, 2H), 1.40 (d, J = 12.6, 2H), 1.34–1.17 (m, 6H), 0.96 (ddd, J = 28.2, 25.4, 12.6, 6H), 0.40 (s, 27H) ppm. ^{13}C{^{1}H} NMR (101 MHz, C_6D_6, 25 °C): δ 145.1 (s), 129.6 (s), 128.7 (s), 127.1 (s), 89.4 (s), 54.5 (s), 37.0 (s), 34.7 (s), 26.5 (s), 26.0 (s), 25.5 (s), 3.6 (s), 2.8 (s) ppm. ^{11}B{^{1}H} NMR (128 MHz, C_6D_6, 25 °C): δ 21.8 (s, 1B, BCl) ppm. ^{29}Si{^{1}H} NMR (99.36 MHz, C_6D_6, 25 °C): δ –70.28 (*Si*(SiMe$_3$)), –13.58 (Si(*Si*Me$_3$)) ppm. ESI-HRMS: Calcd for $[M+H]^+$ $C_{28}H_{55}BClN_2Si_4$: 577.3224, found: 577.3232. Elemental Analysis was unsuccessful.

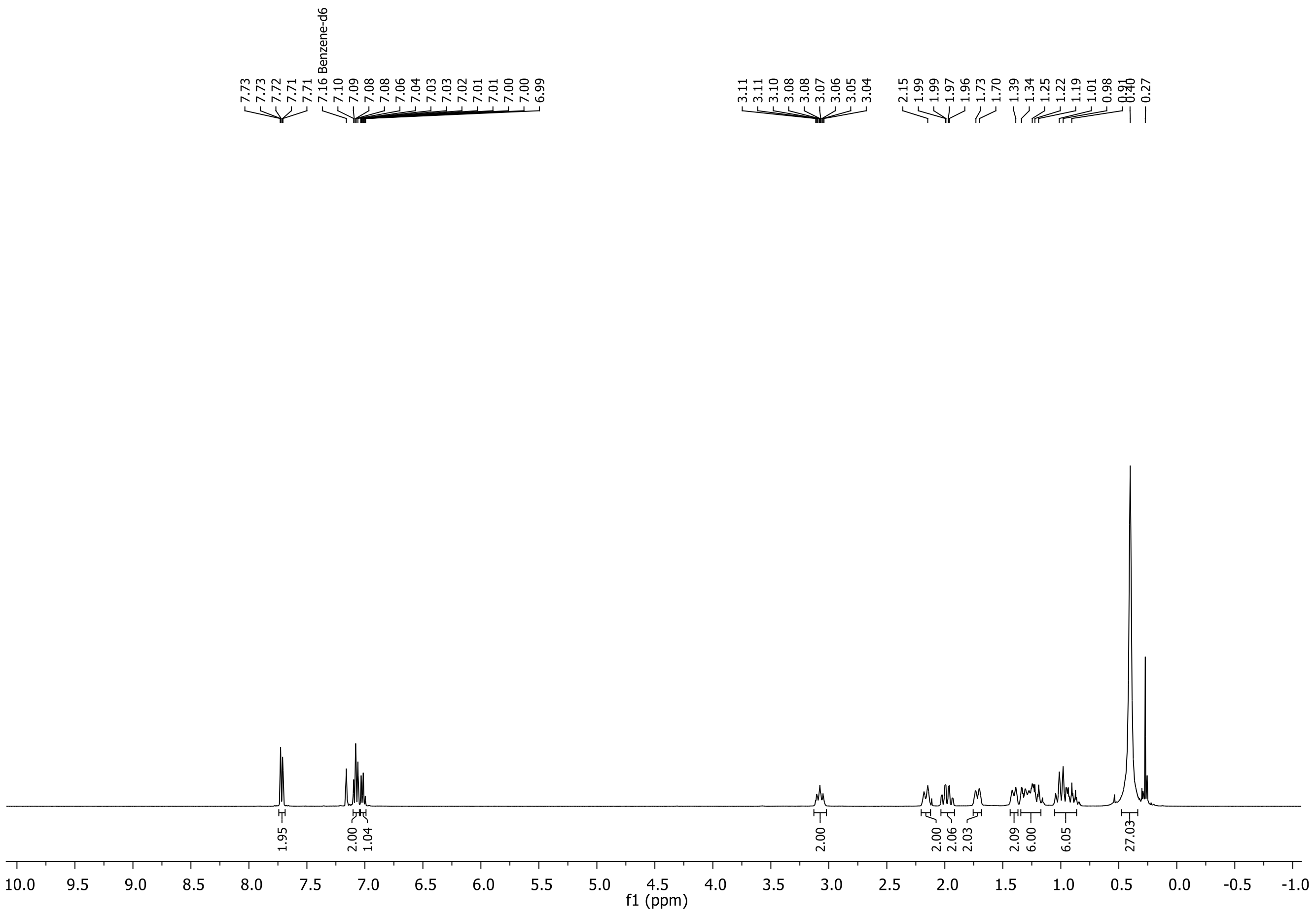

Figure S4. ^{1}H NMR spectrum of **3** (400 MHz, C_6D_6, 25 °C)

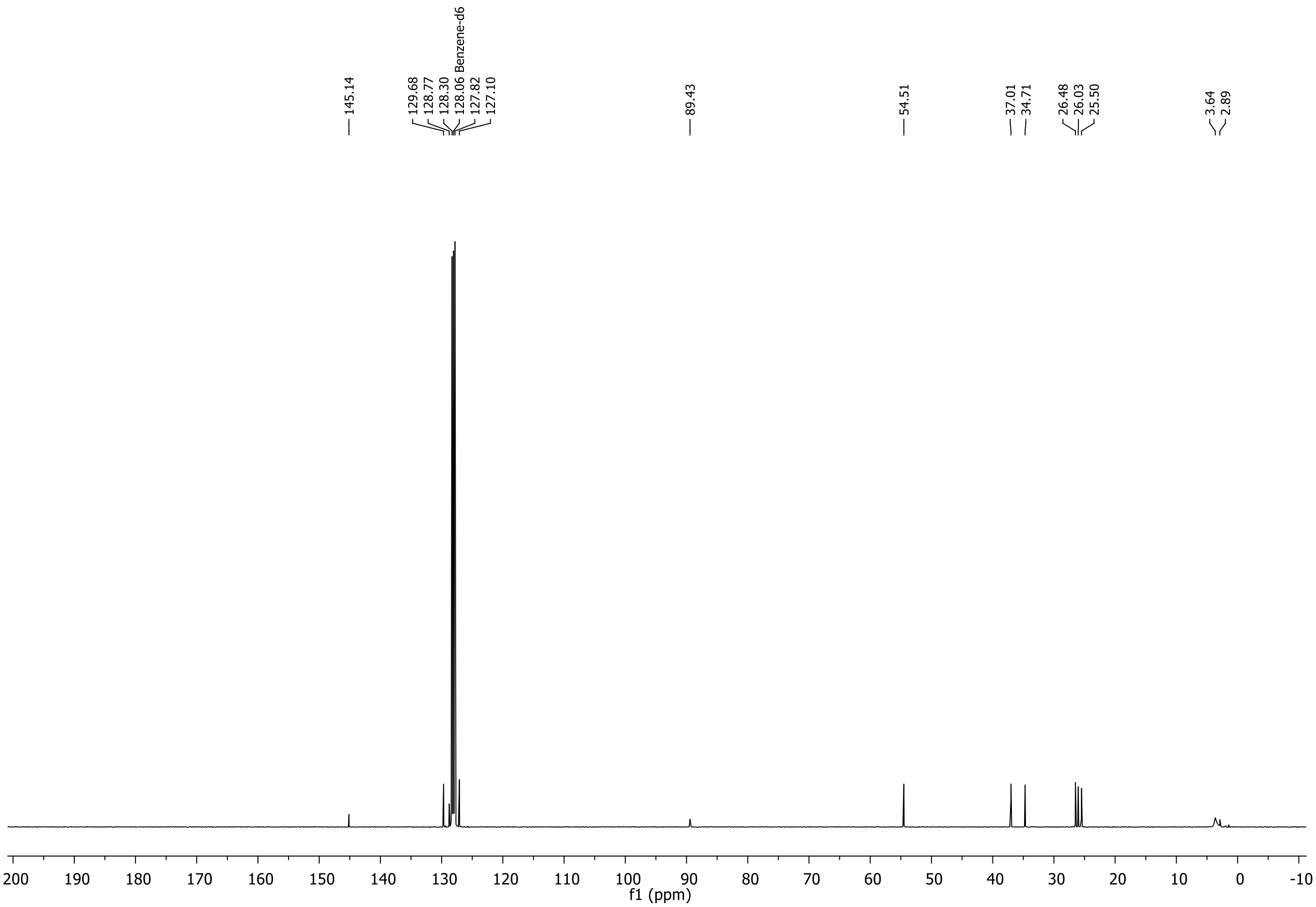

Figure S5.^{13}C NMR spectrum of **3** (101 MHz, C_6D_6, 25 °C)

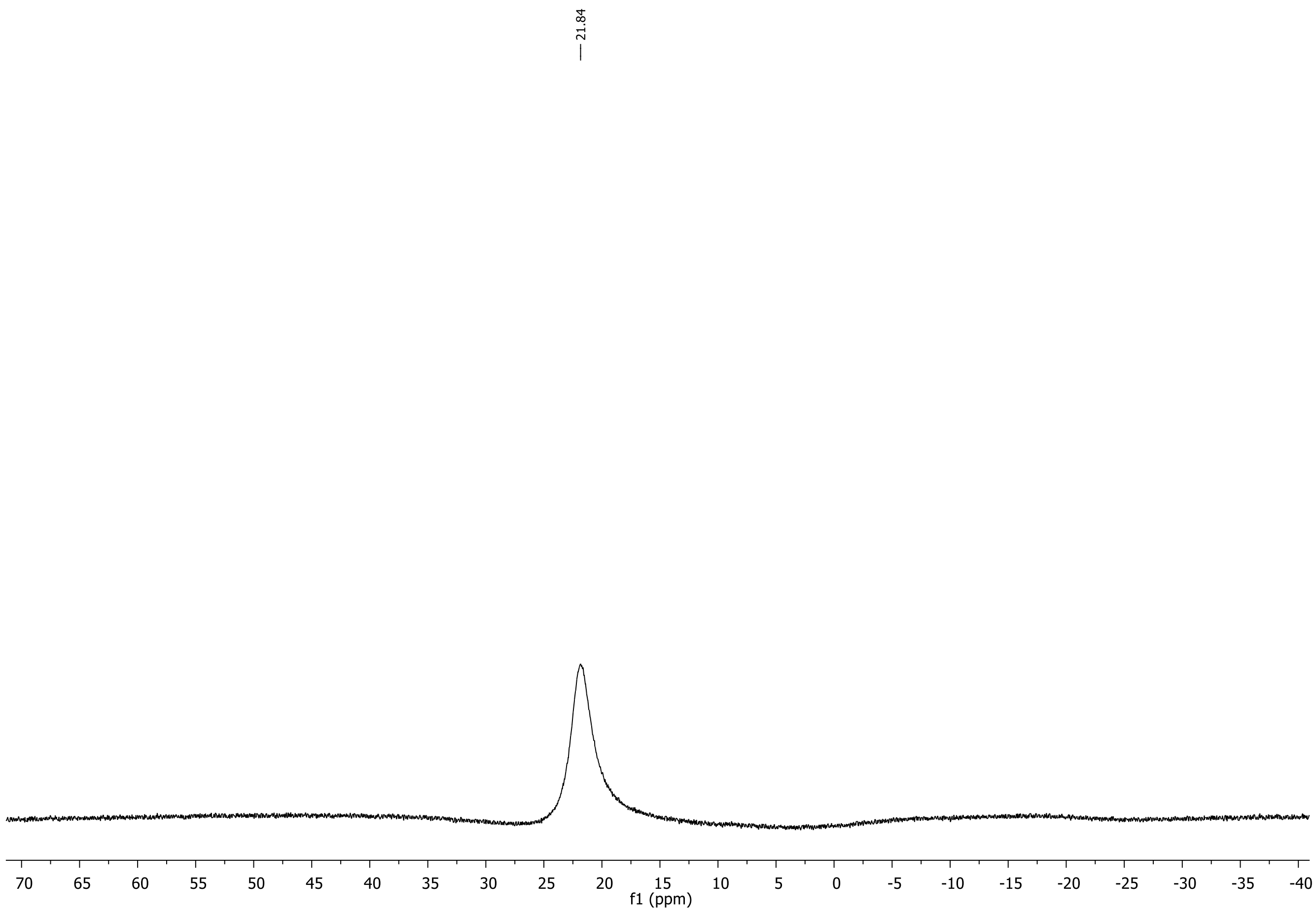

Figure S6. ^{11}B NMR spectrum of **3** (128 MHz, C_6D_6, 25 °C)

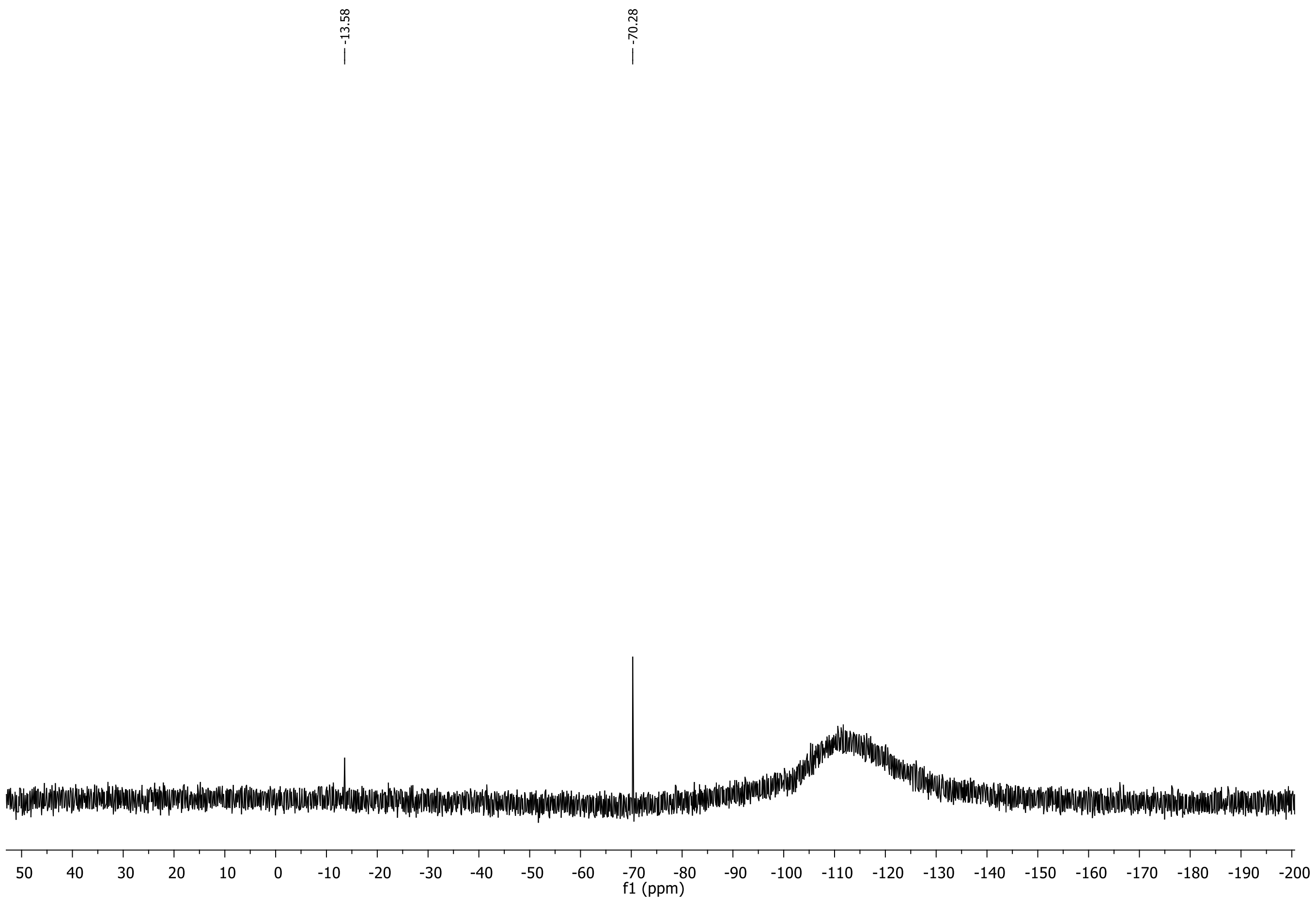

Figure S7. ^{29}Si NMR spectrum of **3** (99.36 MHz, C_6D_6, 25 °C)

Synthesis of 4. A solution of potassium tris(trimethylsilyl)silane (334 mg, 0.775 mmol) in toluene (10 mL) was added to a solution of compound **2** (300 mg, 0.738 mmol) in toluene (8 mL) at –30 °C over a period of 15 min and the color changed slowly from pale yellow to deep red. Then the reaction mixture was allowed to warm up to room temperature and was further stirred for 12 h. Subsequently, all volatiles were removed in vacuo and the residue was extracted into toluene (15 mL). The resultant toluene solvent was concentrated to 5–8 mL and stored at –20 °C in a freezer, which afforded colorless crystals of **4** suitable for single crystal X-ray diffraction analysis within two days. Yield: 430 mg (94%). Mp: 170–172 °C.

4: ^{1}H NMR (400 MHz, C_6D_6, 25 °C): *δ* 7.88 (d, *J* = 6.7, 2H), 7.47 (dd, *J* = 7.1, 2.5, 2H), 7.41 (t, *J* = 7.6, 2H), 7.24 (t, *J* = 7.3, 1H), 7.06–6.98 (m, 3H), 3.33 (dd, *J* = 10.0, 6.6, 2H), 1.92 (d, *J* = 12.5, 2H), 1.75 (d, *J* = 12.2, 2H), 1.38 (d, *J* = 12.9, 4H), 1.22 (dd, *J* = 21.3, 10.7, 4H), 1.09–0.91 (m, 6H), 0.69 (d, *J* = 13.2, 2H), 0.53 (s, 27H) ppm. ^{13}C{^{1}H} NMR (101 MHz, C_6D_6, 25 °C): *δ* 164.6 (s), 134.3 (s), 132.5 (s), 130.3 (s), 128.9 (s), 128.5 (s), 127.1 (s), 127.0 (s), 56.1 (s), 36.2 (s), 34.5 (s), 26.3 (s), 25.9 (s), 25.7 (s), 4.4 (s) ppm. ^{11}B{^{1}H} NMR (128 MHz, C_6D_6, 25 °C): *δ* 9.1 (s, 1B, *B*(Ph)Si($SiMe_3$)$_3$) ppm. ^{29}Si{^{1}H} NMR (99.36 MHz, C_6D_6, 25 °C): *δ* –10.77 (*Si*($SiMe_3$)), –11.30 (Si(*Si*Me_3)) ppm. ESI-HRMS: Calcd for $[M + H]^+$ $C_{34}H_{60}BN_2Si_4$: 619.3927, found: 619.3937. Elemental Analysis was unsuccessful.

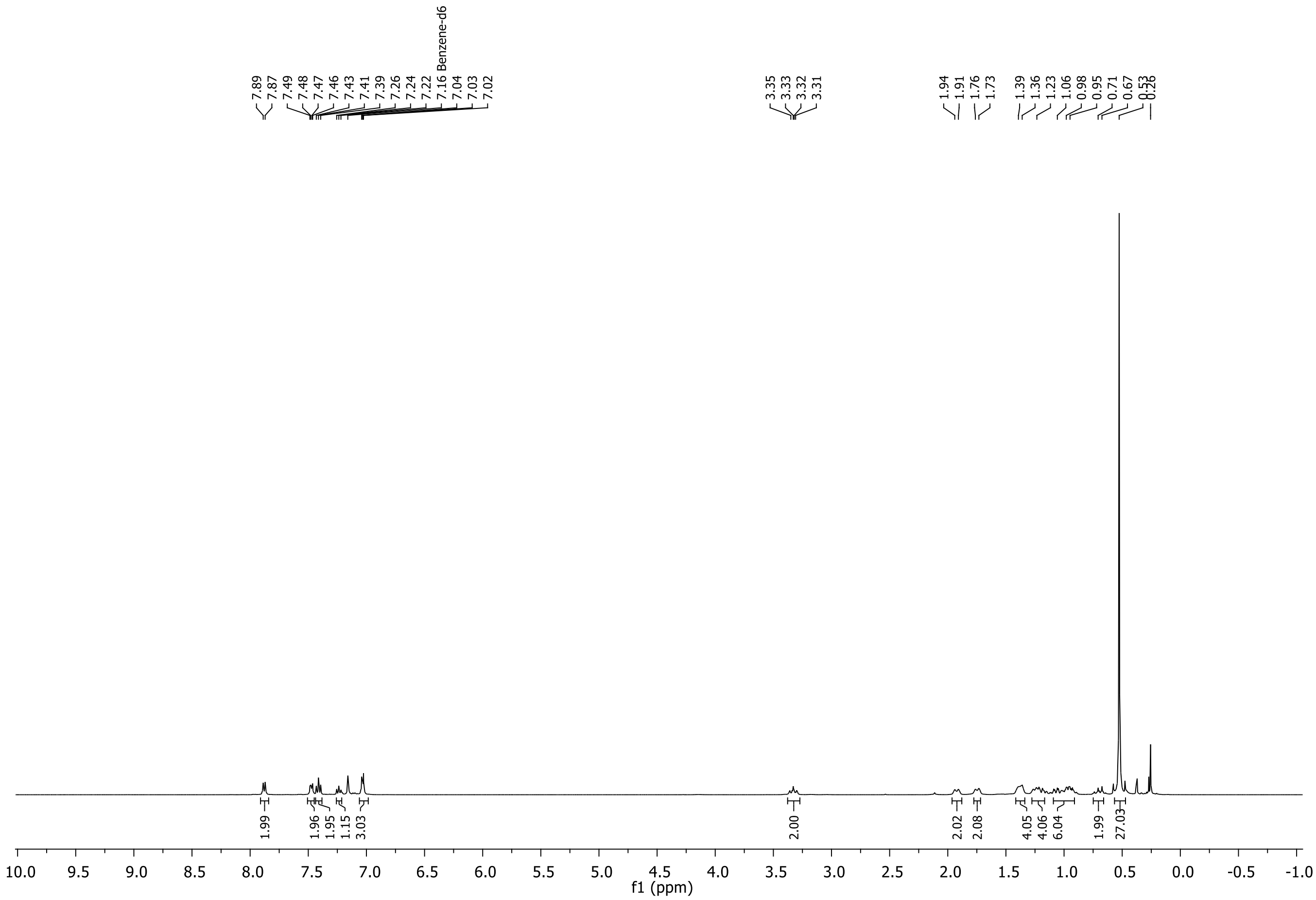

Figure S8. ^{1}H NMR spectrum of **4** (400 MHz, C_6D_6, 25 °C)

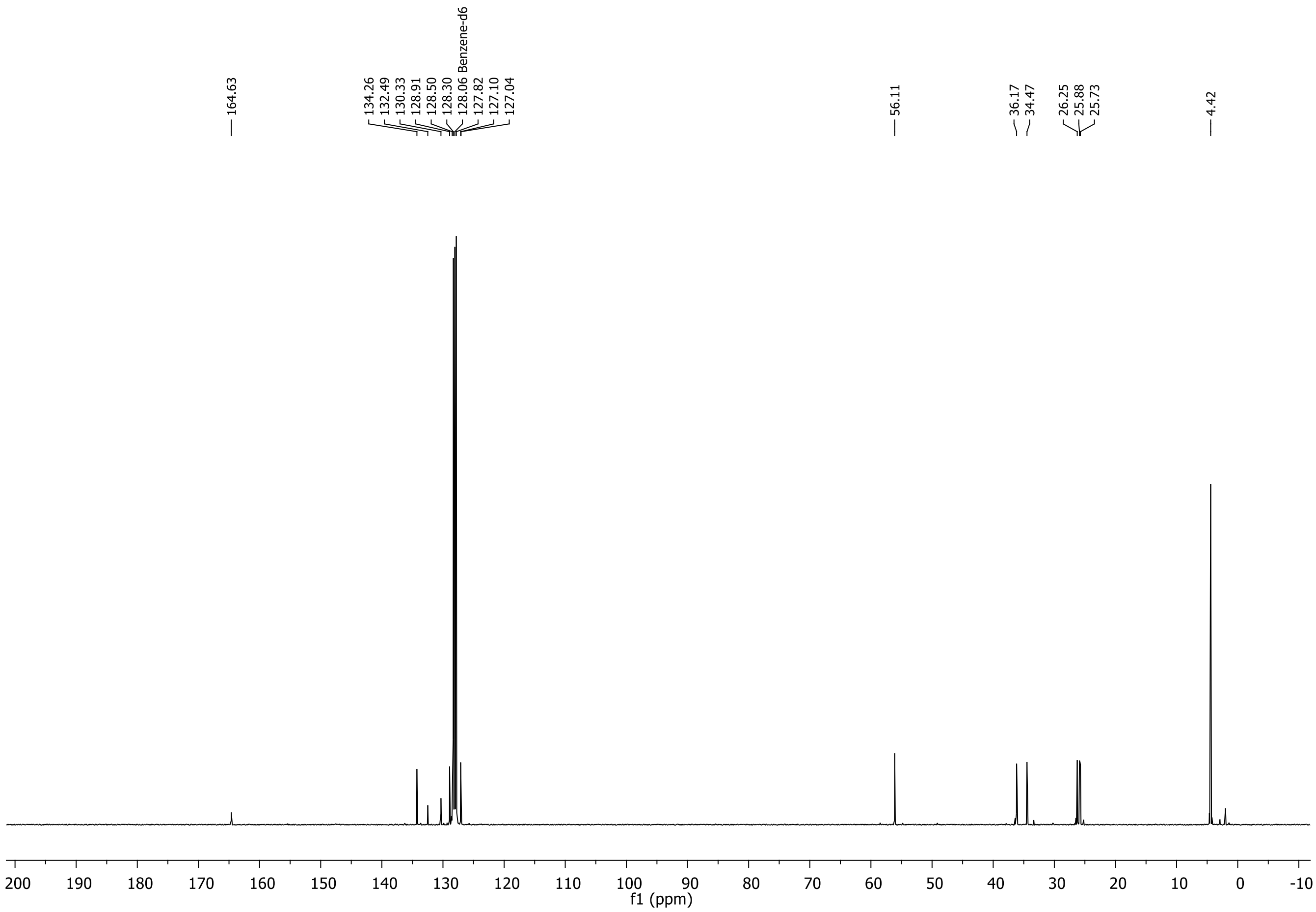

Figure S9. ^{13}C NMR spectrum of **4** (101 MHz, C_6D_6, 25 °C)

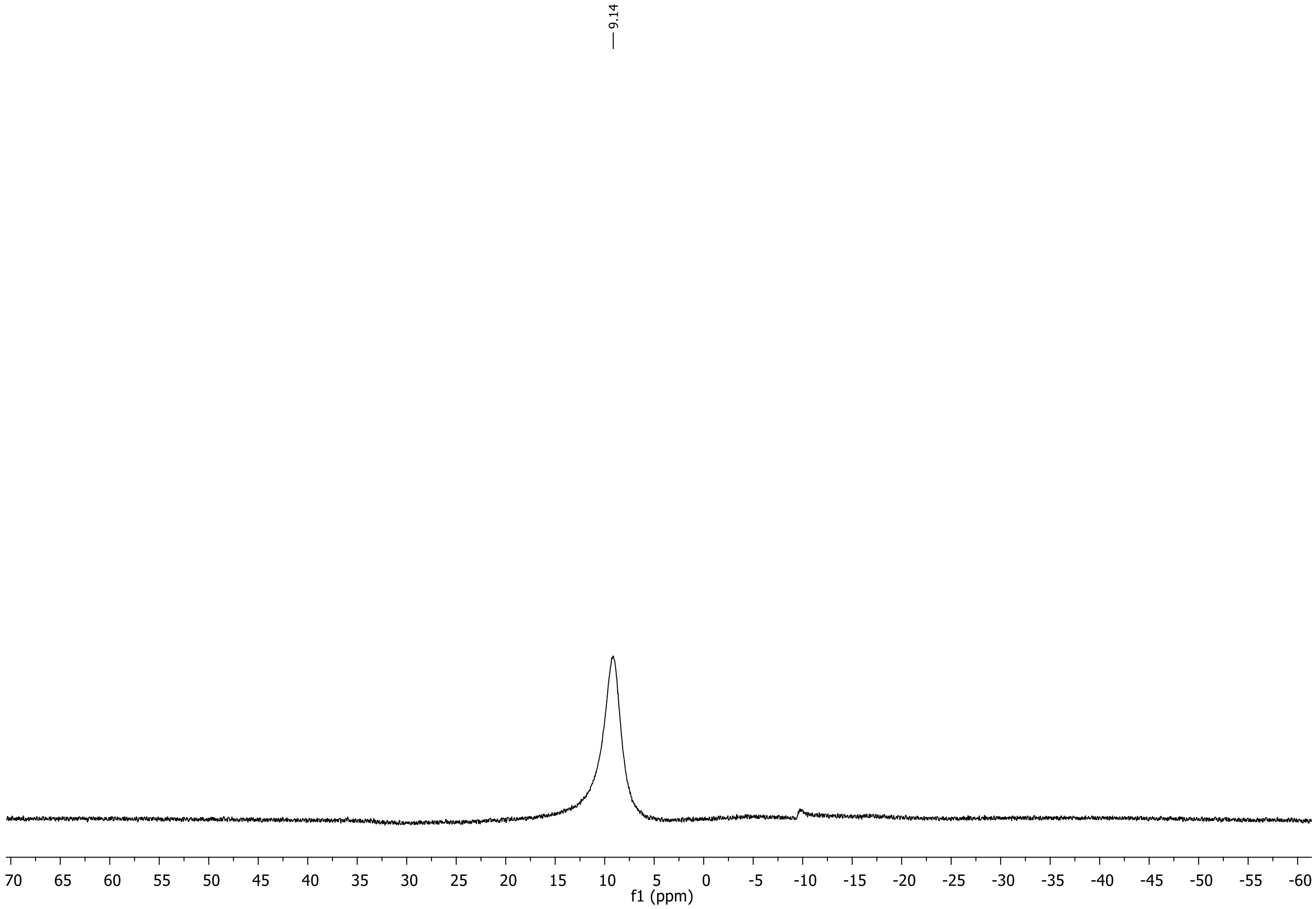

Figure S10. ^{11}B NMR spectrum of **4** (128 MHz, C_6D_6, 25 °C)

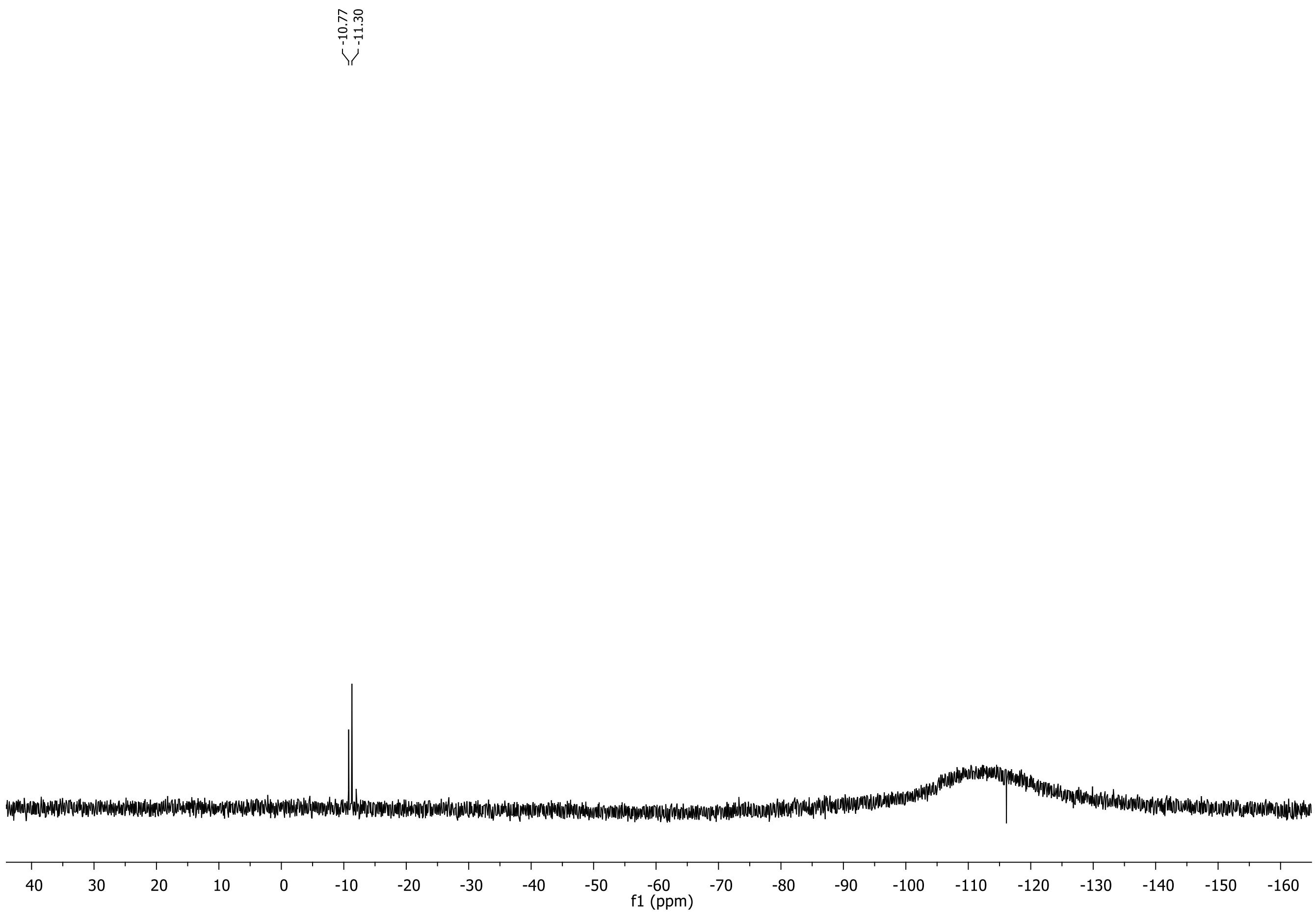

Figure S11. ^{29}Si NMR spectrum of **4** (99.36 MHz, C_6D_6, 25 °C)

Synthesis of 6. A solution of potassium tris(trimethylsilyl)silane (478 mg, 1.1 mmol in toluene 10 mL) was added to a solution of compound **5** (300 mg, 1.05 mmol in toluene 10 mL) at –30 °C over a period of 15 min and the color changed slowly from pale yellow to deep red. Then the reaction mixture was allowed to warm up to room temperature and further stirred for 12 h. Subsequently, all volatiles were removed in vacuo and the residue was extracted into toluene (15 mL). The resultant toluene solvent was concentrated to 5–8 mL and stored at –20 °C in a freezer, which afforded colorless crystals of **6** suitable for single crystal X-ray diffraction analysis within two days. Yield: 493 mg (94%). Mp: 180–182 °C.

6: ^{1}H NMR (400 MHz, C_6D_6, 25 °C): δ 7.67–7.61 (m, 2H), 7.09–6.99 (m, 3H), 3.44–3.34 (m, 2H), 1.42 (d, J = 6.7 Hz, 6H), 0.67 (d, J = 6.2 Hz, 6H), 0.36 (s, 27H) ppm. $^{13}C\{^{1}H\}$ NMR (101 MHz, C_6D_6, 25 °C): δ 144.10 (s), 129.77 (s), 128.69 (s), 127.22 (s), 89.69 (s), 46.42 (s), 25.37 (d, J = 91.7 Hz), 3.60 (s) ppm. $^{11}B\{^{1}H\}$ NMR (128 MHz, C_6D_6, 25 °C): δ 22.1 (s, 1B, BCl) ppm. $^{29}Si\{^{1}H\}$ NMR (99.36 MHz, C_6D_6, 25 °C): δ –69.73 (*Si*(SiMe$_3$)), –13.52 (Si(*Si*Me$_3$)) ppm. ESI-HRMS: Calcd for $[M + H]^+$ $C_{22}H_{47}BClN_2Si_4$: 497.2598, found: 497.2604. Elemental Analysis (%) Calculated for $C_{22}H_{46}BClN_2Si_4$: C = 53.14; H = 9.33; N = 5.63. Found 1: C = 53.40; H = 8.38; N = 4.96. Found 2: C = 53.37; H = 8.23; N = 4.67.

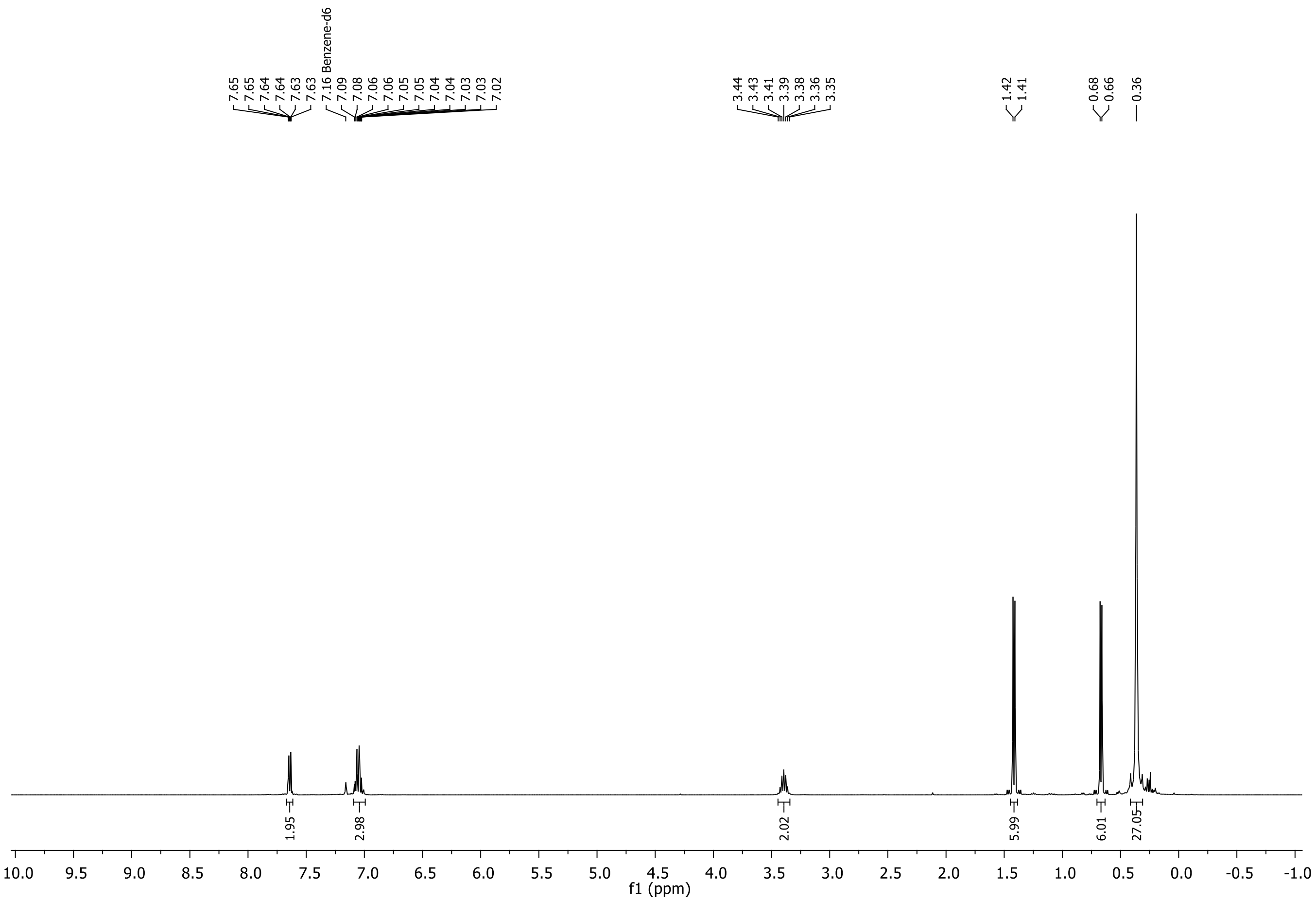

Figure S12. ^{1}H NMR spectrum of **6** (400 MHz, C_6D_6, 25 °C)

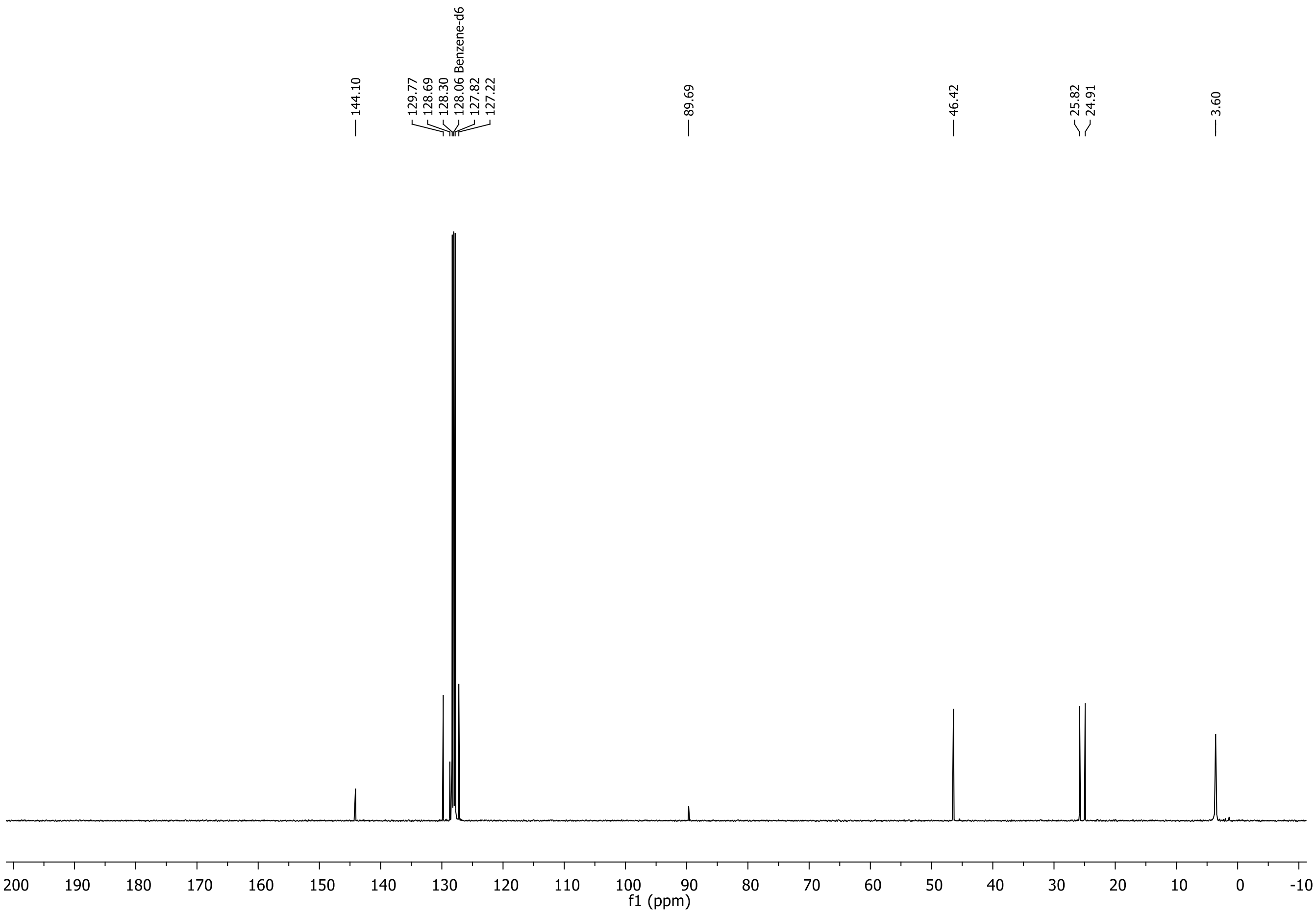

Figure S13. ^{13}C NMR spectrum of **6** (101 MHz, C_6D_6, 25 °C)

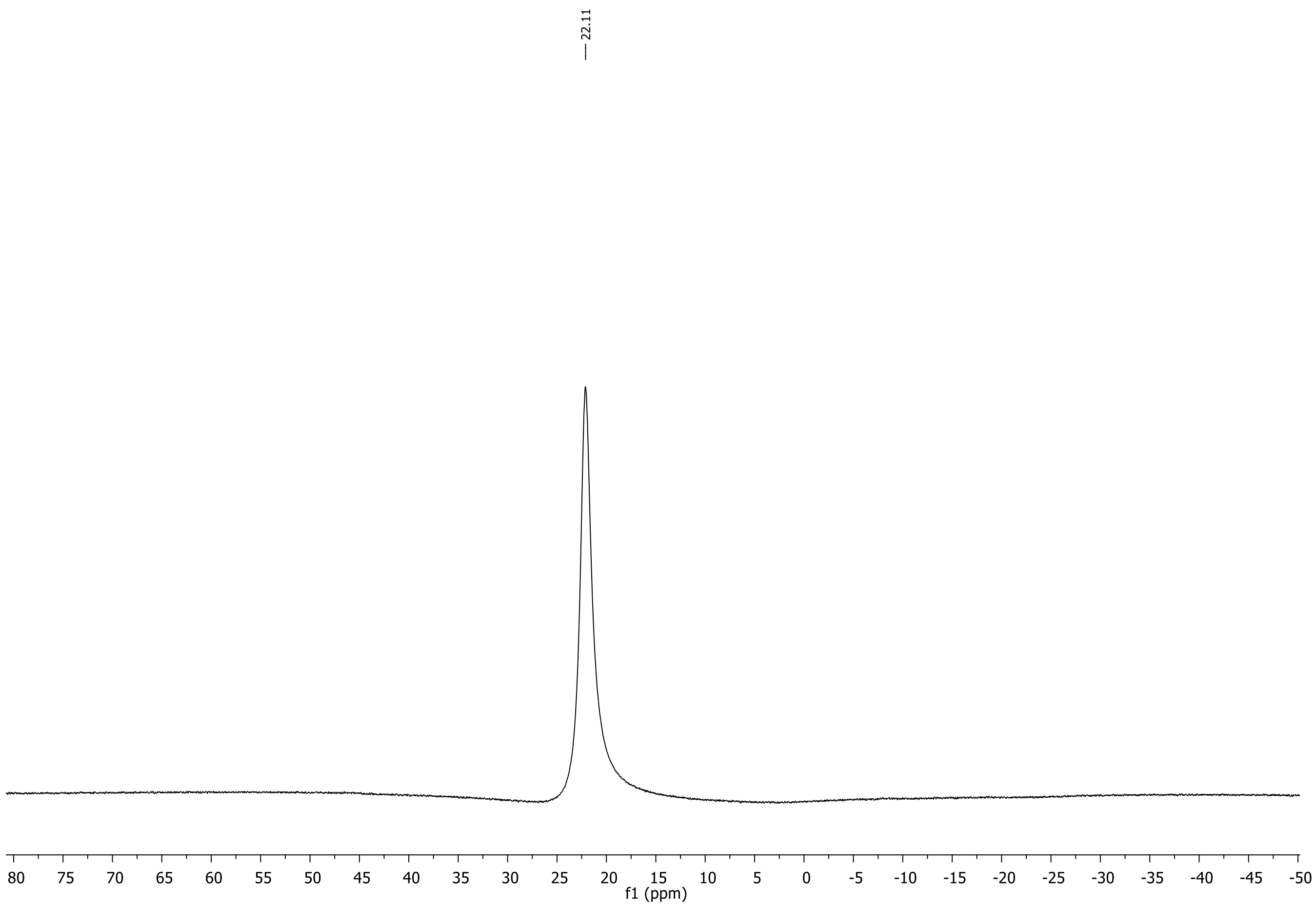

Figure S14. ^{11}B NMR spectrum of **6** (128 MHz, C_6D_6, 25 °C)

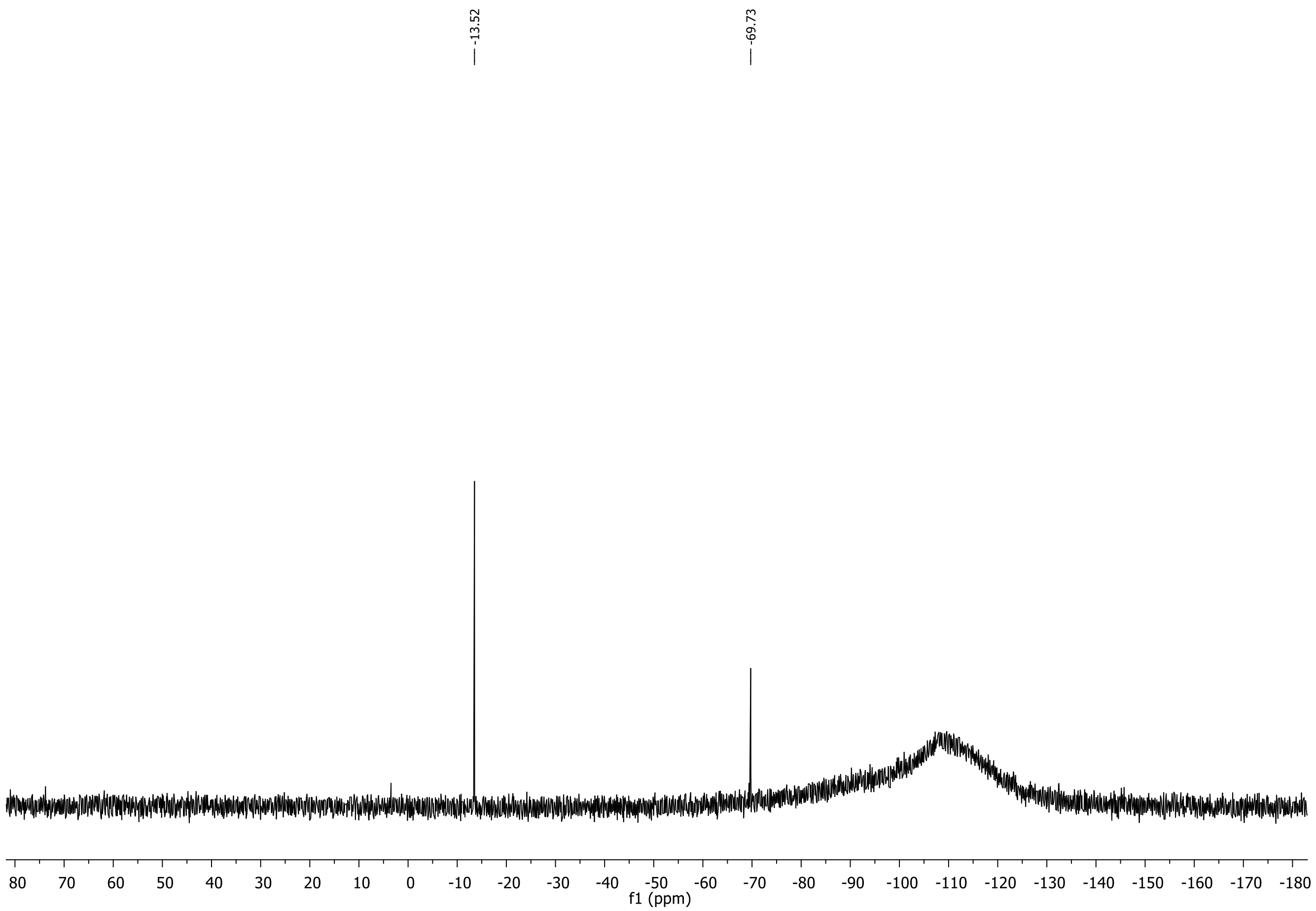

Figure S15. ^{29}Si NMR spectrum of **6** (99.36 MHz, C_6D_6, 25 °C)

Synthesis of 7. ^{t}BuLi (3.15 mL, 5.33 mmol, 1.7 M in diethyl ether) was added dropwise to a solution of *Cy*N=C=N*Cy* (1.0 g, 4.85 mmol) in toluene (50 mL) in a 100 mL Schlenk flask at –78 °C. The solution was warmed to ambient temperature and stirred for 4 h. Then BCl_3 (5.09 mL, 5.09 mmol, 1.0 M in hexane) was added dropwise to the reaction mixture at –30 °C. The reaction mixture was warmed to room temperature and stirred for another 18 h. The white precipitate of the reaction mixture was filtered through Celite. The resultant toluene solvent was removed under reduced pressure and concentrated to 10–12 mL, stored at –20 °C in a freezer,

which afforded colorless crystals of **7** suitable for single crystal X-ray diffraction analysis within one day. Yield: 1.6 g (96%). Mp: 157–159 °C.

7: ^{1}H NMR (400 MHz, C_6D_6, 25 °C): δ 3.49 (d, J = 7.3 Hz, 2H), 2.20–2.11 (m, 4H), 1.98 (d, J = 12.4 Hz, 4H), 1.64 (d, J = 7.5 Hz, 4H), 1.43 (d, J = 8.1 Hz, 2H), 1.03 (d, J = 7.9 Hz, 4H), 0.93 (s, 9H) ppm. ^{13}C{^{1}H} NMR (101 MHz, C_6D_6, 25 °C): δ 179.8 (s), 56.1 (s), 33.8 (s), 27.5 (s), 26.1 (s), 25.5 (s) ppm. ^{11}B{^{1}H} NMR (128 MHz, C_6D_6, 25 °C): δ 5.1 (s, 1B, *B*Cl_2) ppm. ESI-HRMS: Calcd for $[M + H]^+$ $C_{17}H_{32}BCl_2N_2$: 345.2036, found: 345.2042. Elemental Analysis (%) Calculated for $C_{17}H_{31}BCl_2N_2$: C = 59.16; H = 9.05; N = 8.12. Found 1: C = 58.29; H = 8.67; N = 8.10. Found 2: C = 58.85; H = 8.90; N = 7.72.

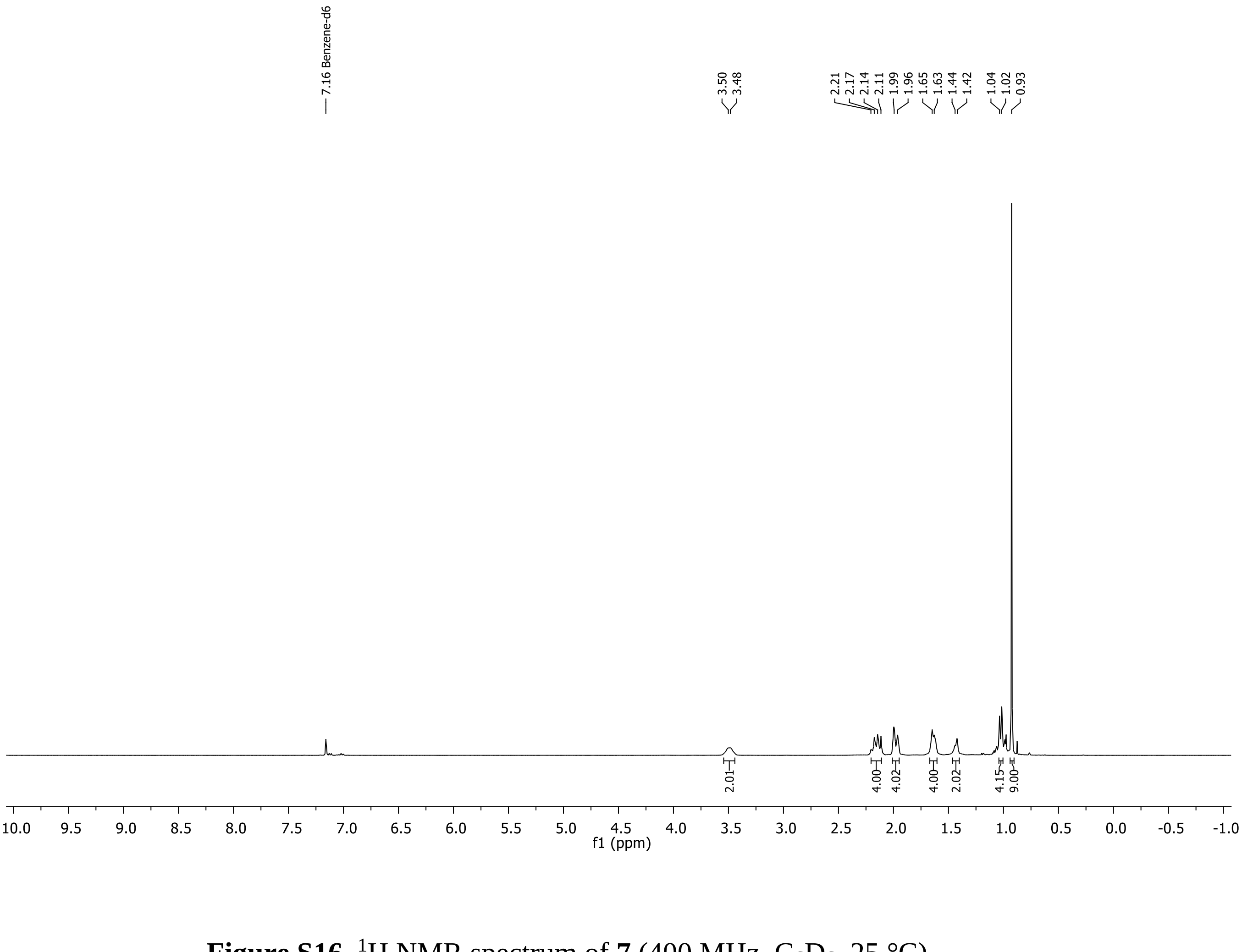

Figure S16. ^{1}H NMR spectrum of **7** (400 MHz, C_6D_6, 25 °C)

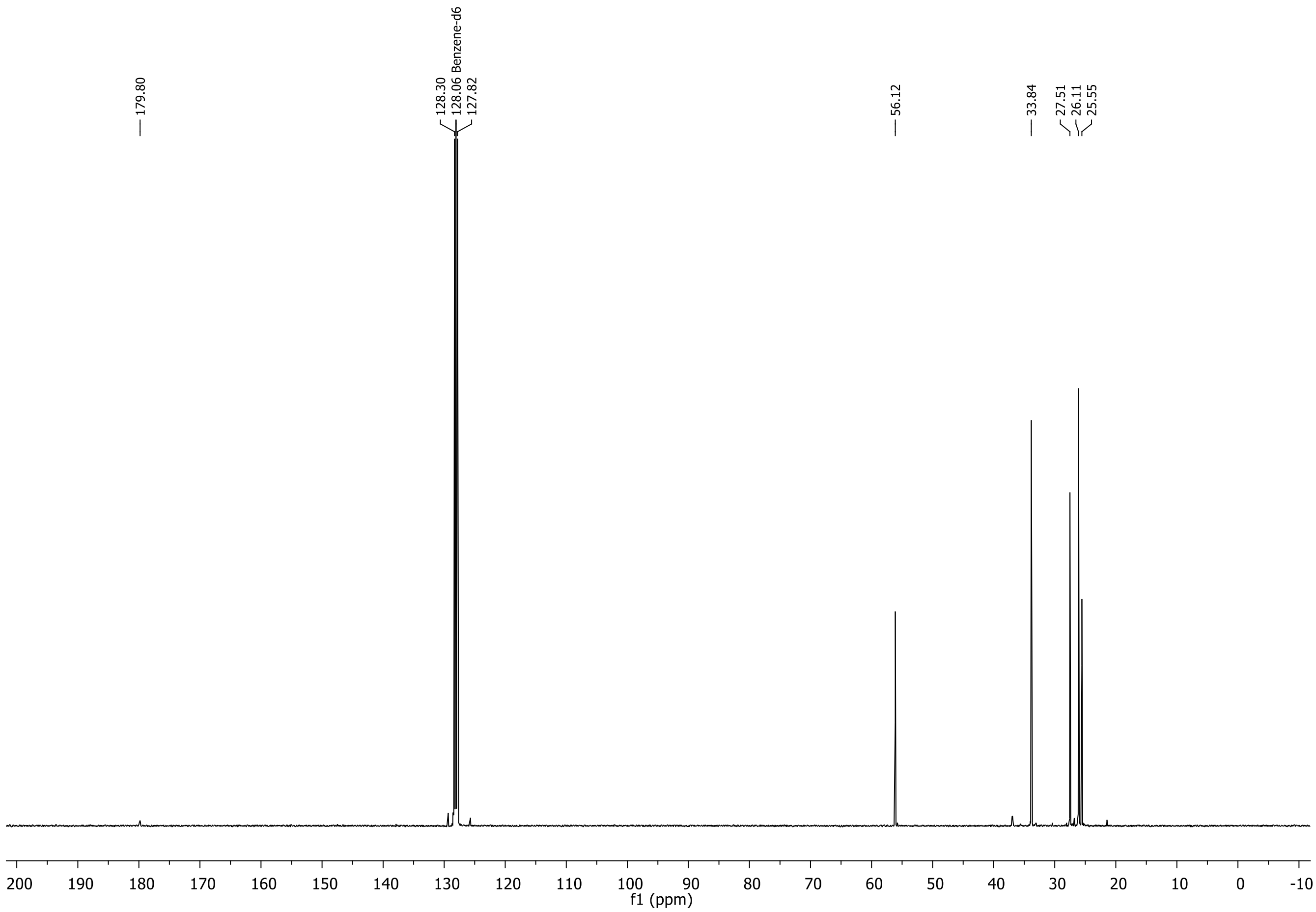

Figure S17. ^{13}C NMR spectrum of **7** (101 MHz, C_6D_6, 25 °C)

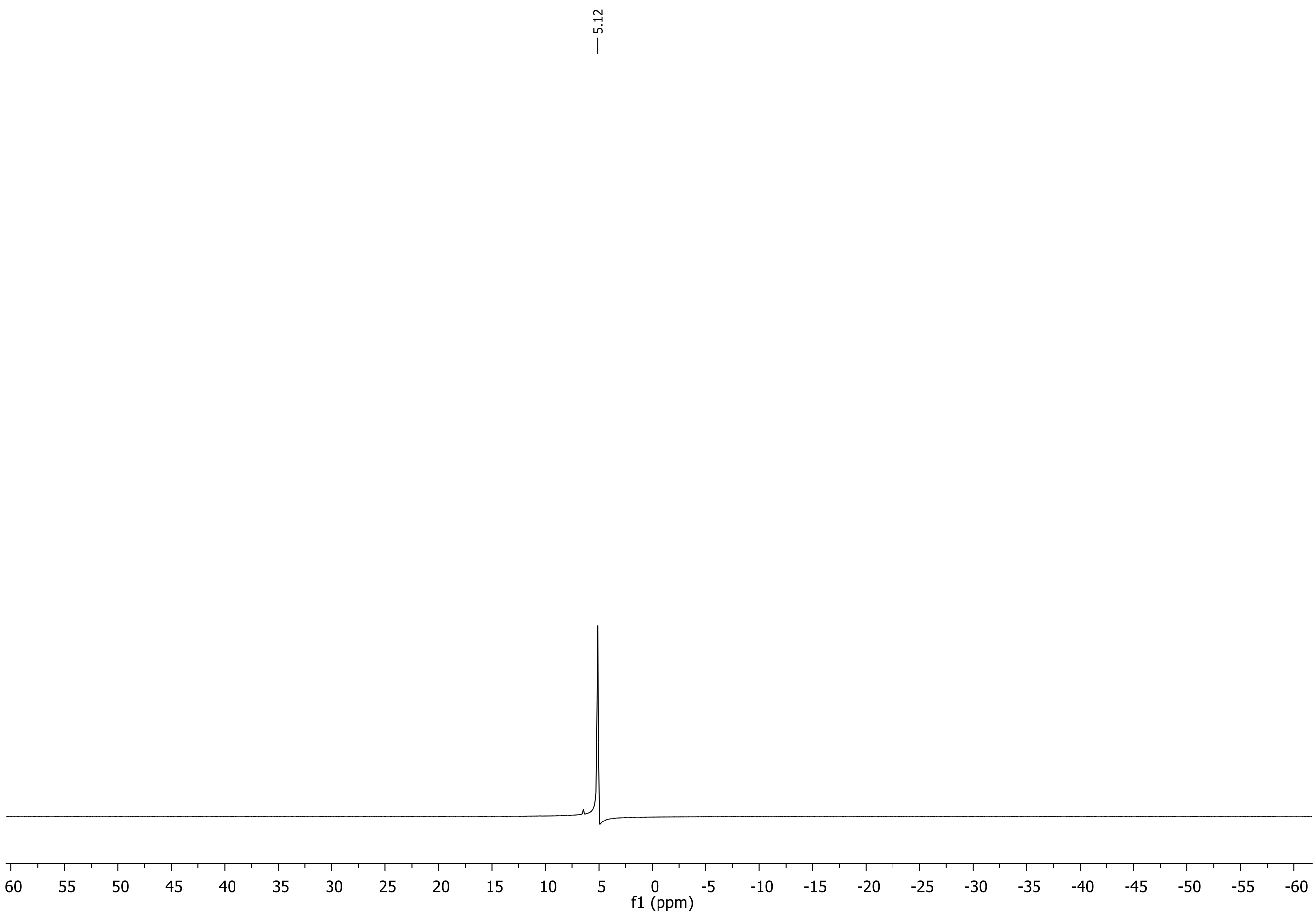

Figure S18. ^{11}B NMR spectrum of **7** (128 MHz, C_6D_6, 25 °C)

Synthesis of 8. A solution of potassium tris(trimethylsilyl)silane (395 mg, 0.915 mmol in toluene 10 mL) was added to a solution of compound **5** (300 mg, 0.871 mmol in toluene 15 mL) at –30 °C over a period of 15 min and the color changed slowly from pale yellow to deep red. Then the reaction mixture was allowed to warm up to room temperature and further stirred for 12 h. Subsequently, all volatiles were removed in vacuo and the residue was extracted into toluene (15 mL). The resultant toluene solvent was concentrated to 5–8 mL and stored at –20 °C in a freezer, which afforded colorless crystals of **8** suitable for single crystal X-ray diffraction analysis within two days. Yield: 456 mg (94%). Mp: 188–190 °C.

8: ^{1}H NMR (400 MHz, C_6D_6, 25 °C): δ 7.12 (d, J = 7.5 Hz, 1H, toluene), 7.03 (dd, J = 16.3, 7.0 Hz, 2H, toluene), 2.95 (t, J = 14.5 Hz, 2H), 2.33 (s, 2H), 1.74–1.61 (m, 4H), 1.13 (s, 9H), 1.07 (d, J = 11.4 Hz, 4H), 0.49 (s, 12H), 0.42 (s, 27H), 0.37 (s, 4H) ppm. ^{13}C{^{1}H} NMR (101 MHz, C_6D_6, 25 °C): δ 137.8 (s), 129.3 (s), 128.5 (s), 101.0 (s), 54.2 (s), 37.4 (s), 36.6 (s), 27.0 (s), 26.6 (s), 25.9 (s), 7.3 (s), 5.5 (s), 4.6 (s), 2.9 (s), 1.9 (s) ppm. ^{11}B{^{1}H} NMR (128 MHz, C_6D_6, 25 °C): δ 20.8 (s, 1B, *B*Cl) ppm. ^{29}Si{^{1}H} NMR (99.36 MHz, C_6D_6, 25 °C): δ –71.62 (*Si*(SiMe$_3$)), –12.05 (Si(*Si*Me$_3$)) ppm. ESI-HRMS: Calcd for $[M + H]^+$ $C_{26}H_{59}BClN_2Si_4$: 557.3537, found: 557.3547. Elemental Analysis was unsuccessful.

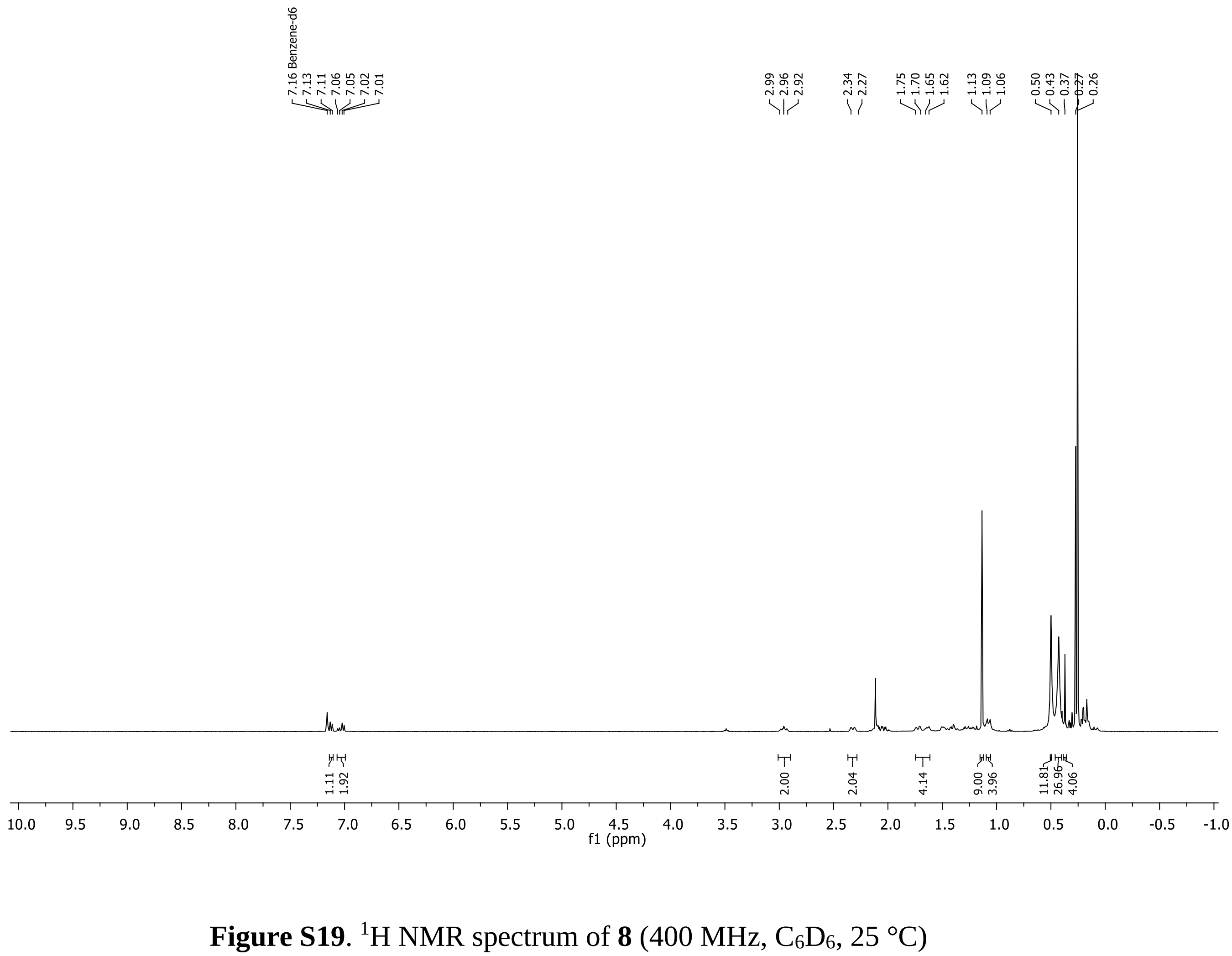

Figure S19. ^{1}H NMR spectrum of **8** (400 MHz, C_6D_6, 25 °C)

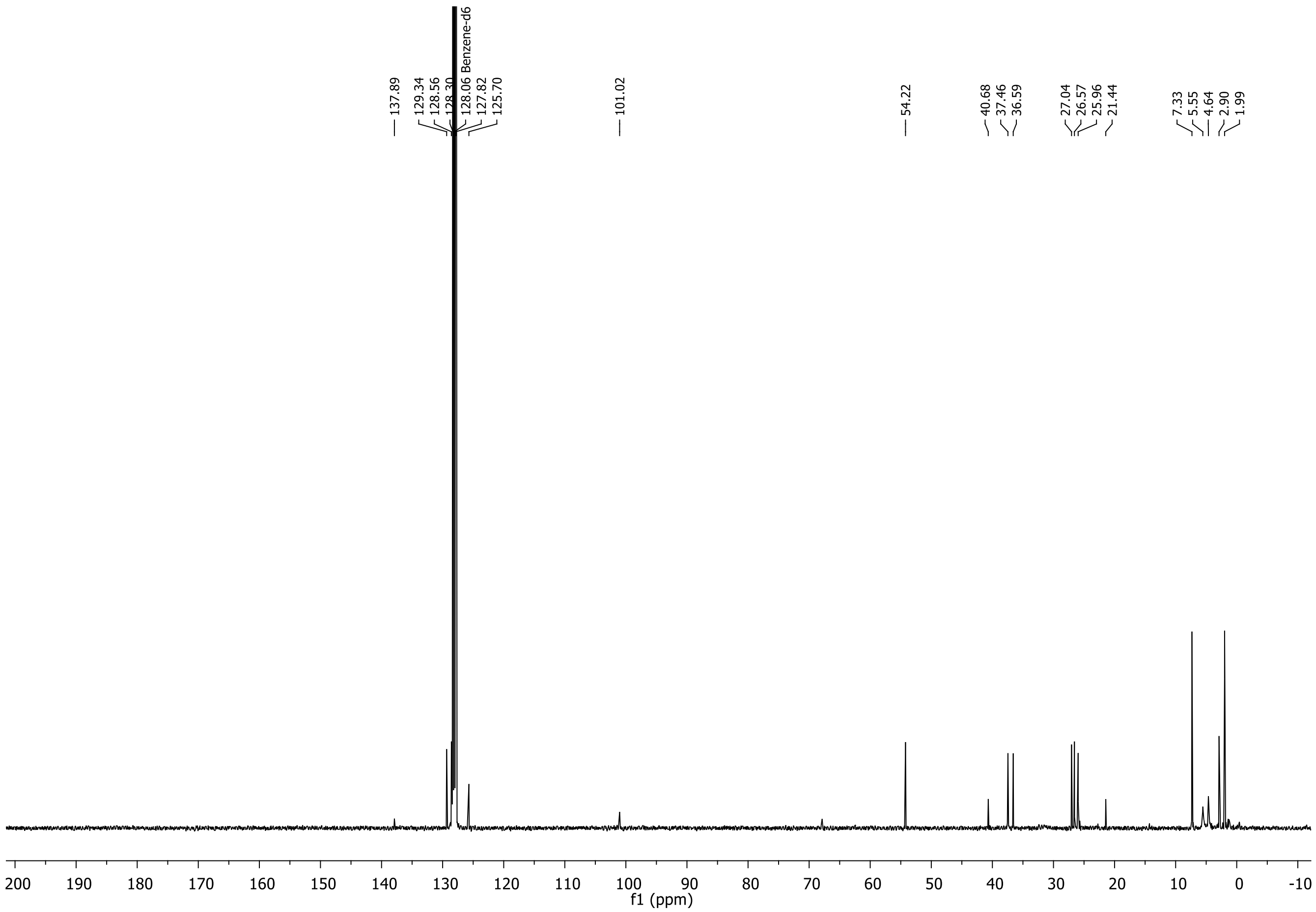

Figure S20. ^{13}C NMR spectrum of **8** (101 MHz, C_6D_6, 25 °C)

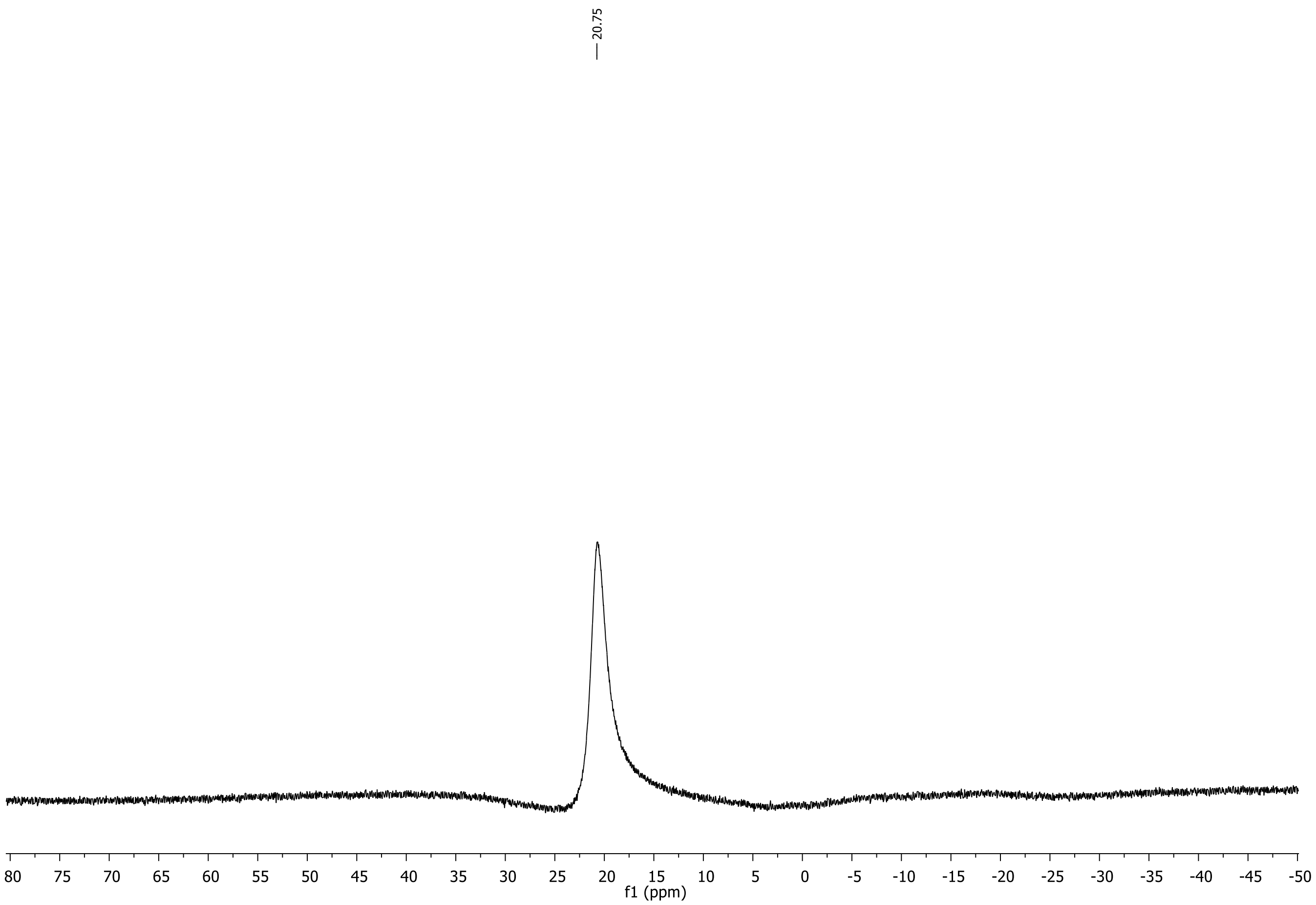

Figure S21. ^{11}B NMR spectrum of **8** (128 MHz, C_6D_6, 25 °C)

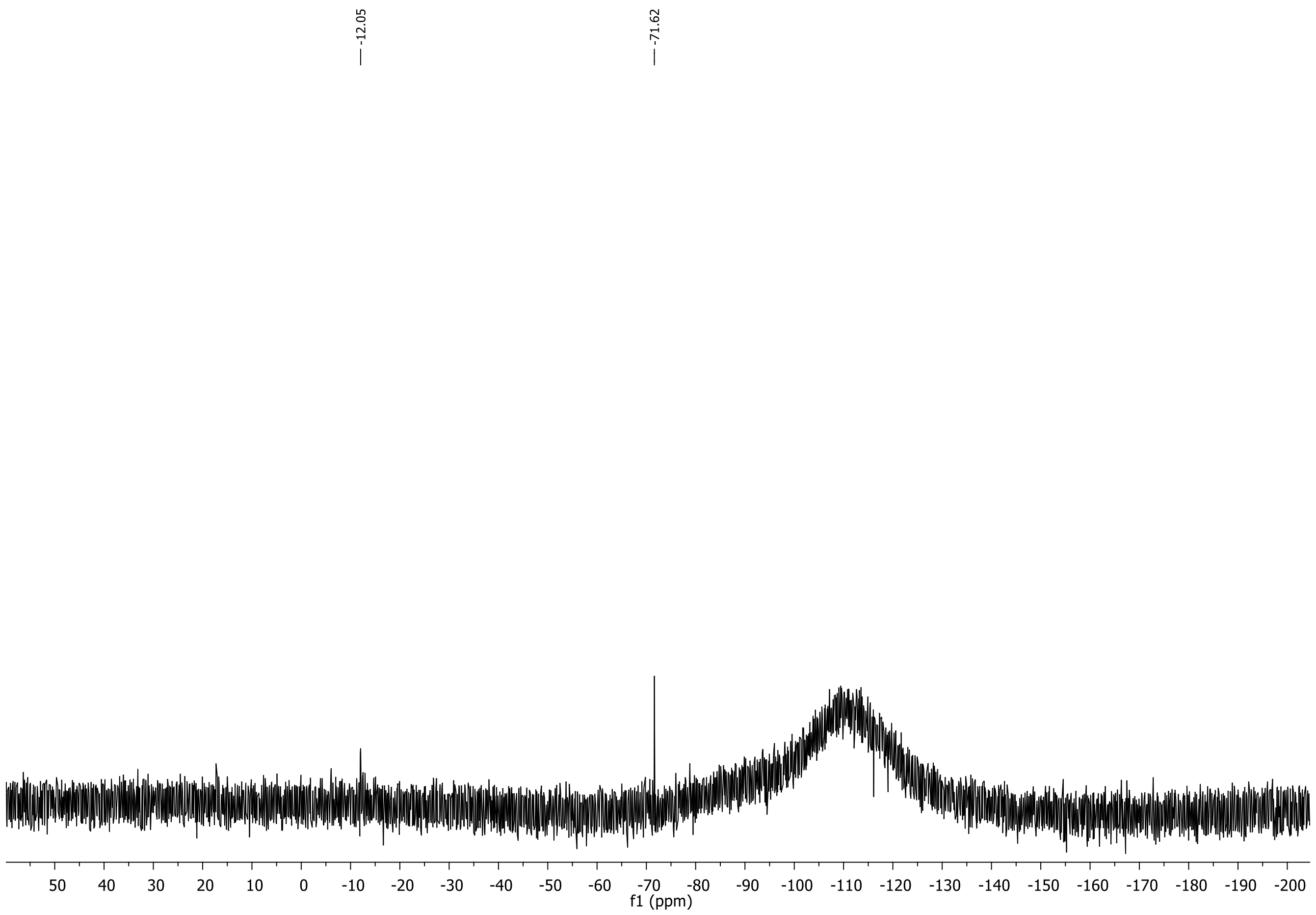

Figure S22. ^{29}Si NMR spectrum of **8** (99.36 MHz, C_6D_6, 25 °C)

Synthesis of 9. A solution of ^{n}BuLi (1.75 mL, 4.35 mmol, 2.5 M in diethyl ether) was added dropwise to a solution 1-bromo-2,6-diisopropylbenzene (Dipp-Br) (1.0 g, 4.15 mmol) in THF (15 mL) in a 100 mL Schlenk flask at –30 °C. The solution was warmed to room temperature and stirred for 4 h. The resulting Dipp-Li solution was again cooled to –30 °C and an equimolar amount of *Cy*N=C=N*Cy* (856 mg, 4.15 mmol) in toluene (15 mL) was added and stirred for another 4 h. Then, BCl_3 (4.35 mL, 4.35 mmol, 1.0 M in hexane) was added dropwise to the reaction mixture at –30 °C. The reaction mixture was warmed to room temperature and stirred

for 18 h. The reaction mixture was dried and extracted with toluene. The white precipitate of the reaction mixture was filtered through Celite. The resultant toluene filtrate was removed under reduced pressure and concentrated to 10–12 mL, stored at –20 °C in a freezer, which afforded colorless crystals of **9** suitable for single crystal X-ray diffraction analysis within one day. Yield: 1.6 g (86%). Mp: 110–112 °C.

9: ^{1}H NMR (400 MHz, C_6D_6, 25 °C): δ 7.15–7.10 (m, 1H), 6.96 (d, *J* = 7.8 Hz, 2H), 3.17–3.08 (m, 2H), 3.05 (dd, *J* = 12.0, 5.6 Hz, 2H), 2.00 (ddd, *J* = 27.0, 12.6, 3.1 Hz, 7H), 1.61–1.52 (m, 5H), 1.37–1.23 (m, 4H), 1.17–1.13 (d, *J* = 7.8 Hz, 12H), 1.13 (s, 2H) ppm. ^{13}C{^{1}H} NMR (101 MHz, C_6D_6, 25 °C): δ172.7 (s), 148.0 (s), 132.0 (s), 124.6 (s), 124.0 (s), 54.9 (s), 34.0 (s), 31.0 (s), 25.4 (d, *J* = 18.3 Hz), 24.7 (s), 23.1 (s) ppm. ^{11}B{^{1}H} NMR (128 MHz, C_6D_6, 25 °C): δ 6.35 (s, 1B, *B*Cl_2) ppm. ESI-HRMS: Calcd for $[M + H]^+$ $C_{25}H_{40}BCl_2N_2$: 449.2662, found: 449.2665. Elemental Analysis was unsuccessful.

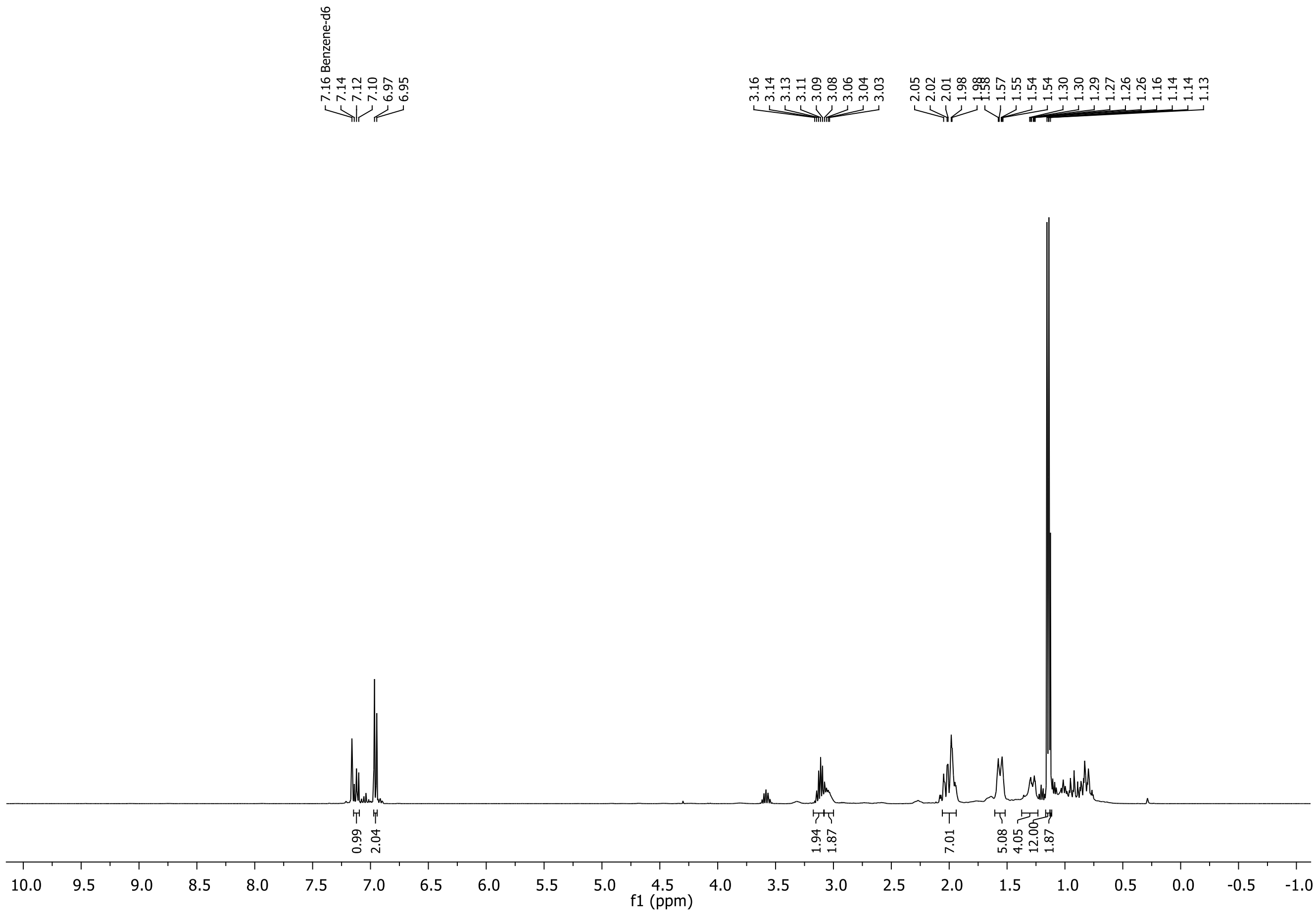

Figure S23. ^{1}H NMR spectrum of **9** (400 MHz, C_6D_6, 25 °C)

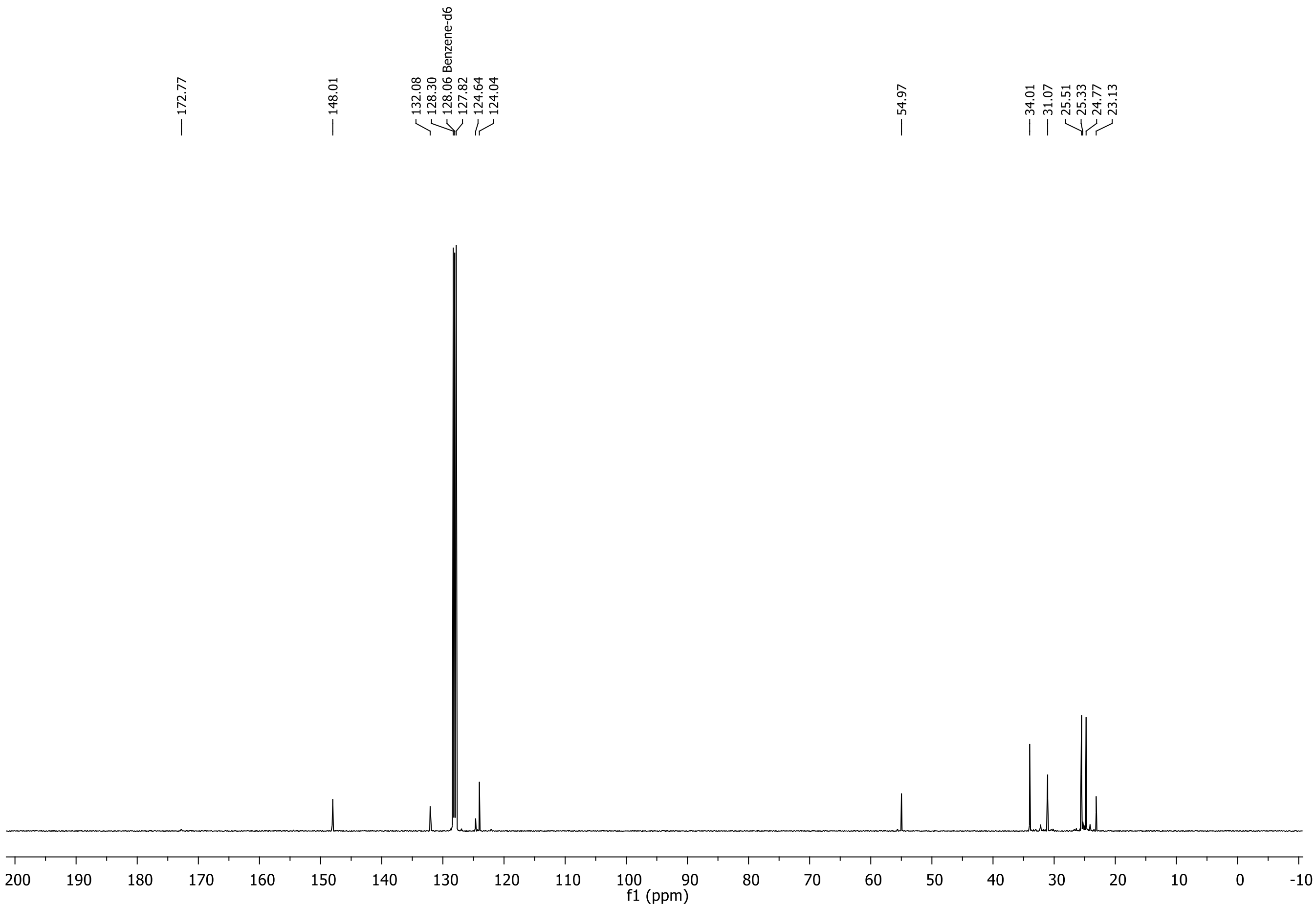

Figure S24. ^{13}C NMR spectrum of **9** (101 MHz, C_6D_6, 25 °C)

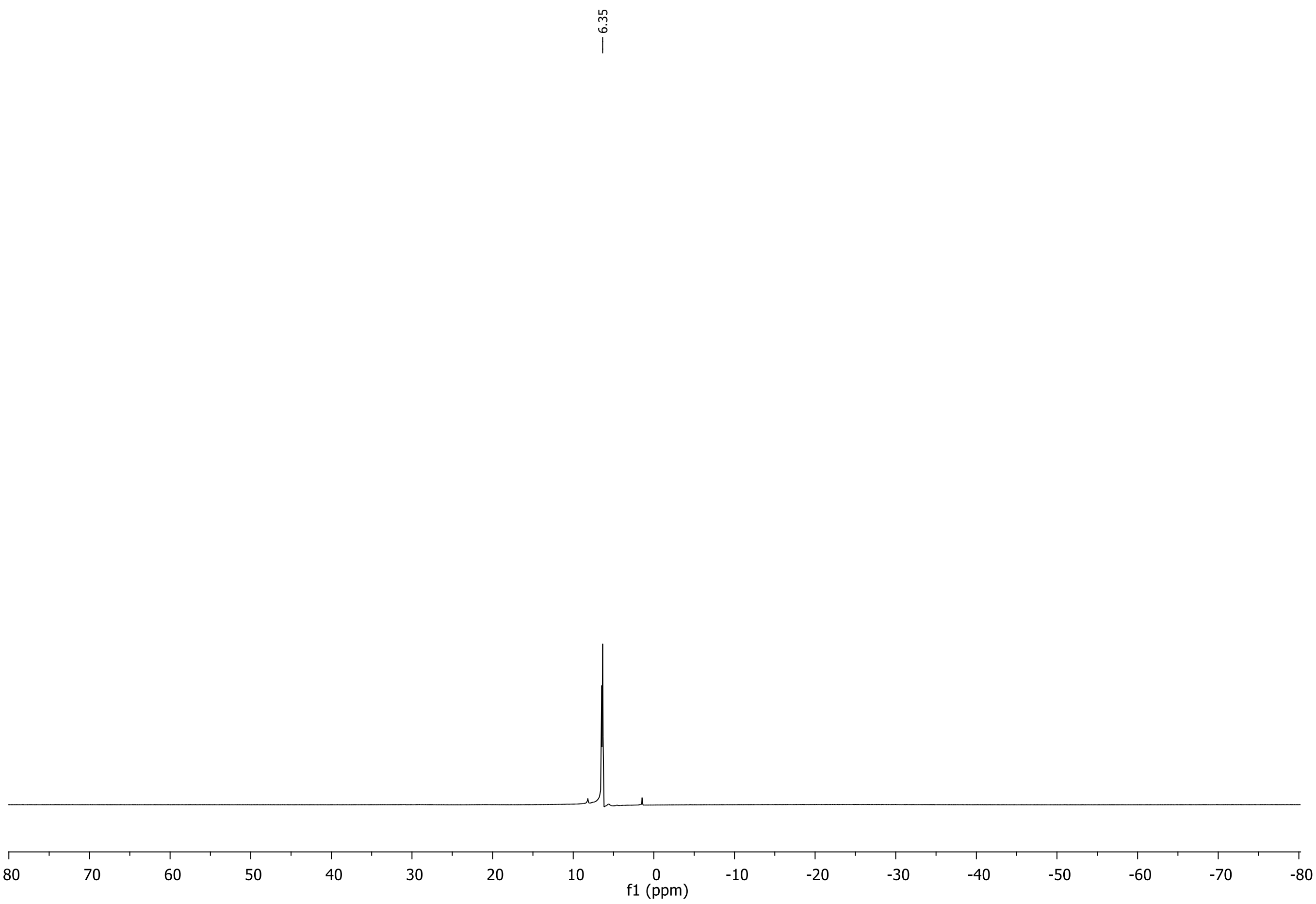

Figure S25. ^{11}B NMR spectrum of **9** (128 MHz, C_6D_6, 25 °C)

Synthesis of 11. $AlCl_3$ (23.1 mg, 0.173 mmol) was added directly to a solution of compound **3** (100 mg, 0.173 mmol in 1 of mL C_6D_6) and was stirred for 15 h inside a glove box. The reaction mixture was filtered and stored at –4 °C in a freezer, which afforded colorless crystals of **11** suitable for single crystal X-ray diffraction within two days. Yield: 90 mg (73%). Mp: 262–264 °C.

11: ^1H NMR (400 MHz, C_6D_6, 25 °C): δ 7.71 (d, J = 8.2 Hz, 2H), 7.08 (t, J = 7.5 Hz, 2H), 7.02 (d, J = 7.2 Hz, 1H), 3.07 (td, J = 11.1, 3.4 Hz, 2H), 2.16 (d, J = 13.1 Hz, 2H), 1.98 (dt, J = 13.1, 9.4 Hz, 2H), 1.72 (d, J = 14.9 Hz, 2H), 1.44–1.30 (m, 6H), 1.20 (d, J = 10.3 Hz, 2H), 1.03–0.90

(m, 6H), 0.40 (s, 27H) ppm. $^{11}B\{^{1}H\}$ NMR (128 MHz, C_6D_6, 25 °C): δ 28.7 (s, 1B, *B*(NCy)Cl) ppm. Due to negligible solubility in common deuterated solvents once crystallized, so no meaningful solution state spectroscopic data of ^{13}C, ^{27}Al and ^{29}Si NMR spectrum could be acquired. Attempts were made to obtain solution state spectroscopic data before crystallization, but NMR data show unidentified product mixtures along with 11. Elemental Analysis was unsuccessful.

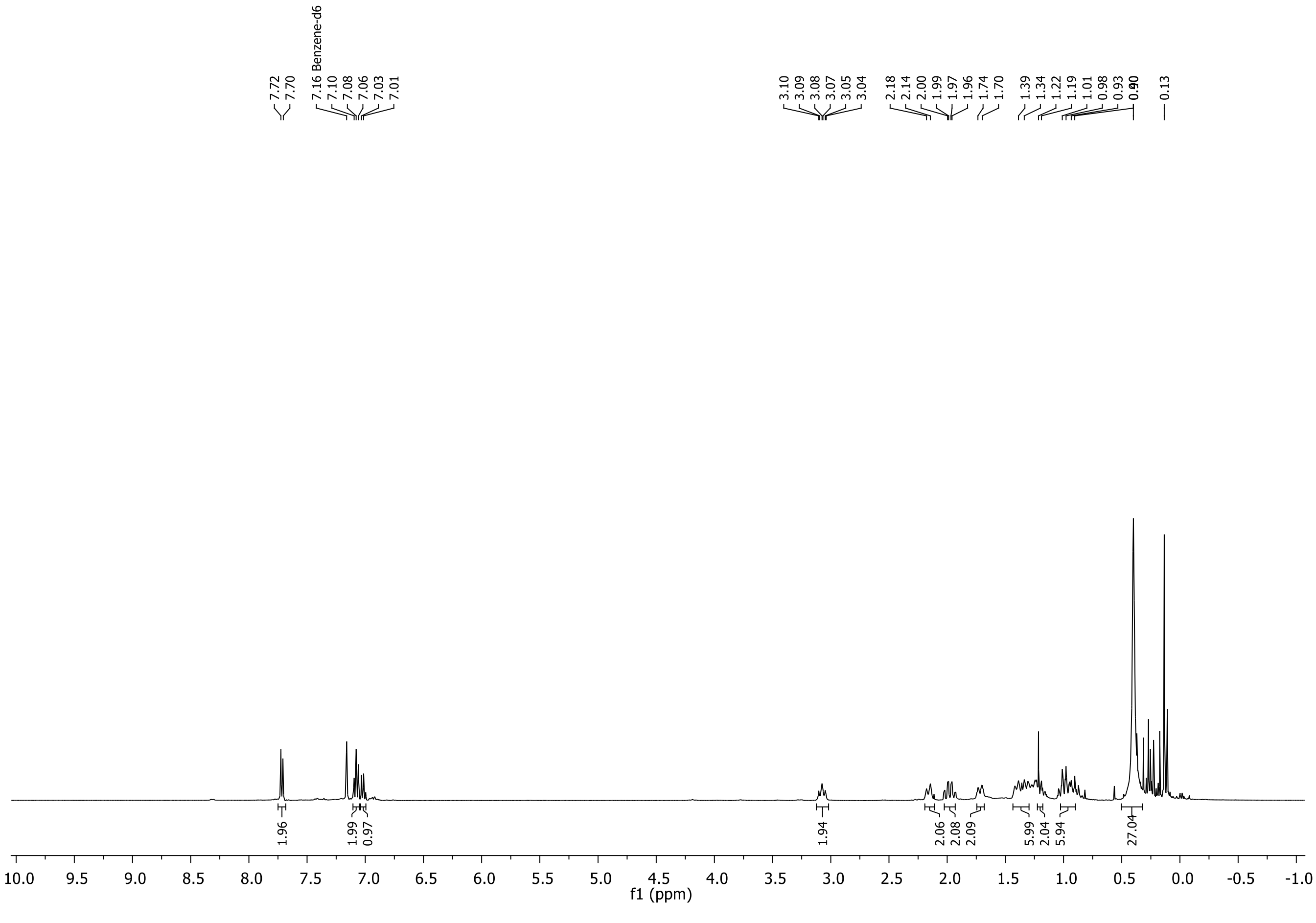

Figure S26. ^{1}H NMR spectrum of **11** (400 MHz, C_6D_6, 25 °C)

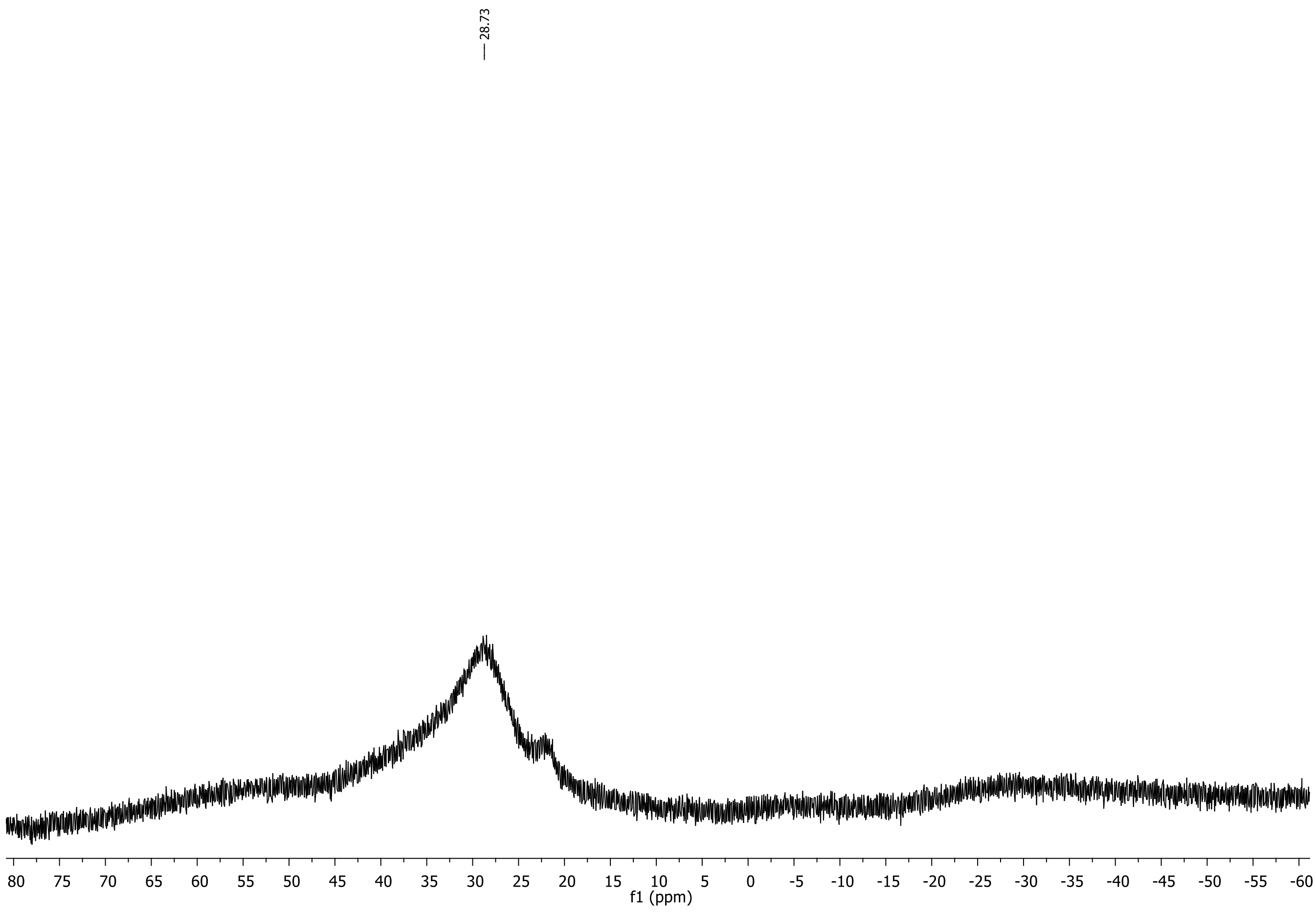

Figure S27. ^{11}B NMR spectrum of **11** (128 MHz, C_6D_6, 25 °C)

Synthesis of 12. A solution of phenyl lithium (0.19 mL, 0.364 mmol, 1.9 M in diethyl ether) was added dropwise to a solution of compound **3** (200 mg, 0.347 mmol in toluene 10 mL) at –40 °C over a period of 8–10 min. The reaction mixture was allowed to warm up to room temperature and further stirred for 36 h. Subsequently, all volatiles were removed in vacuo and the residue was extracted into toluene (15 mL). The resultant toluene filtrate was concentrated to 6–8 mL and stored at –30 °C in a freezer, which afforded colorless crystals of **12** suitable for single crystal X-ray diffraction analysis within two days. Yield: 200 mg (93%). Mp: 177–179 °C.

12: ^{1}H NMR (400 MHz, C_6D_6, 25 °C): δ 7.99–7.94 (m, 2H), 7.89 (dd, J = 8.0, 1.3 Hz, 2H), 7.32 (t, J = 7.3 Hz, 2H), 7.23–7.19 (m, 2H), 7.09 (t, J = 7.3 Hz, 2H), 3.04 (t, J = 10.8 Hz, 2H), 1.96 (d, J = 12.7 Hz, 2H), 1.50 (t, J = 10.9 Hz, 4H), 1.37 (d, J = 9.5 Hz, 4H), 1.31 (d, J = 9.4 Hz, 6H), 0.94 (d, J = 12.8 Hz, 4H), 0.46 (s, 27H) ppm. $^{13}C\{^{1}H\}$ NMR (101 MHz, C_6D_6, 25 °C): δ 146.8 (s), 133.6 (s), 129.8 (s), 128.4 (s), 127.1 (s), 54.8 (s), 37.8 (s), 36.1 (s), 30.2 (s), 26.6 (s), 25.9 (s), 25.7 (s), 3.7 (s) ppm. $^{11}B\{^{1}H\}$ NMR (128 MHz, C_6D_6, 25 °C): δ 32.8 (s, 1B, *B*(Ph)) ppm. $^{29}Si\{^{1}H\}$ NMR (99.36 MHz, C_6D_6, 25 °C): δ –73.29 (*Si*($SiMe_3$)), –13.42 (Si(*Si*Me_3)) ppm. ESI-HRMS: Calcd for $[M + H]^+$ $C_{34}H_{60}BN_2Si_4$: 619.3927, found: 619.3935. Elemental Analysis was unsuccessful.

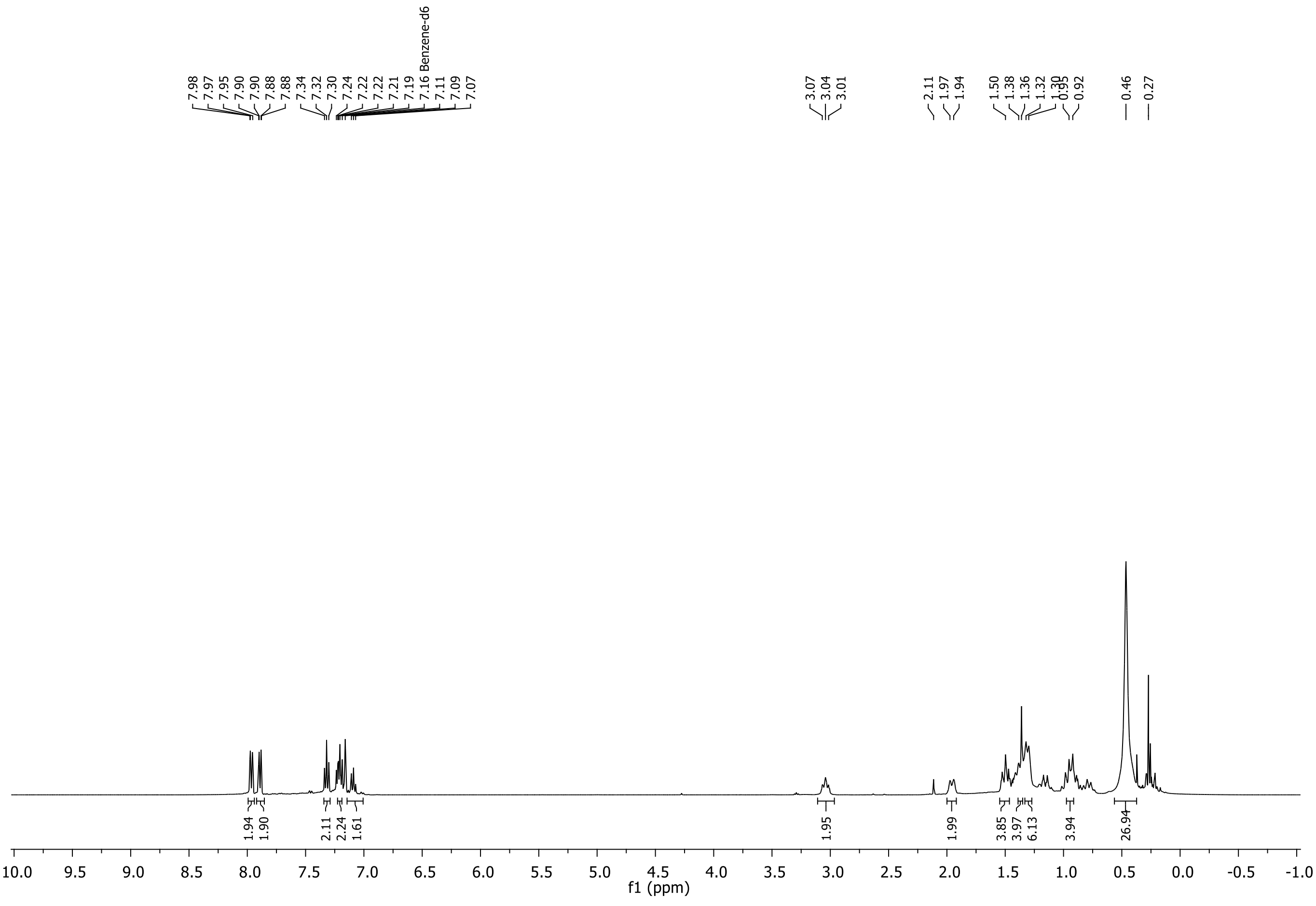

Figure S28. ^{1}H NMR spectrum of **12** (400 MHz, C_6D_6, 25 °C)

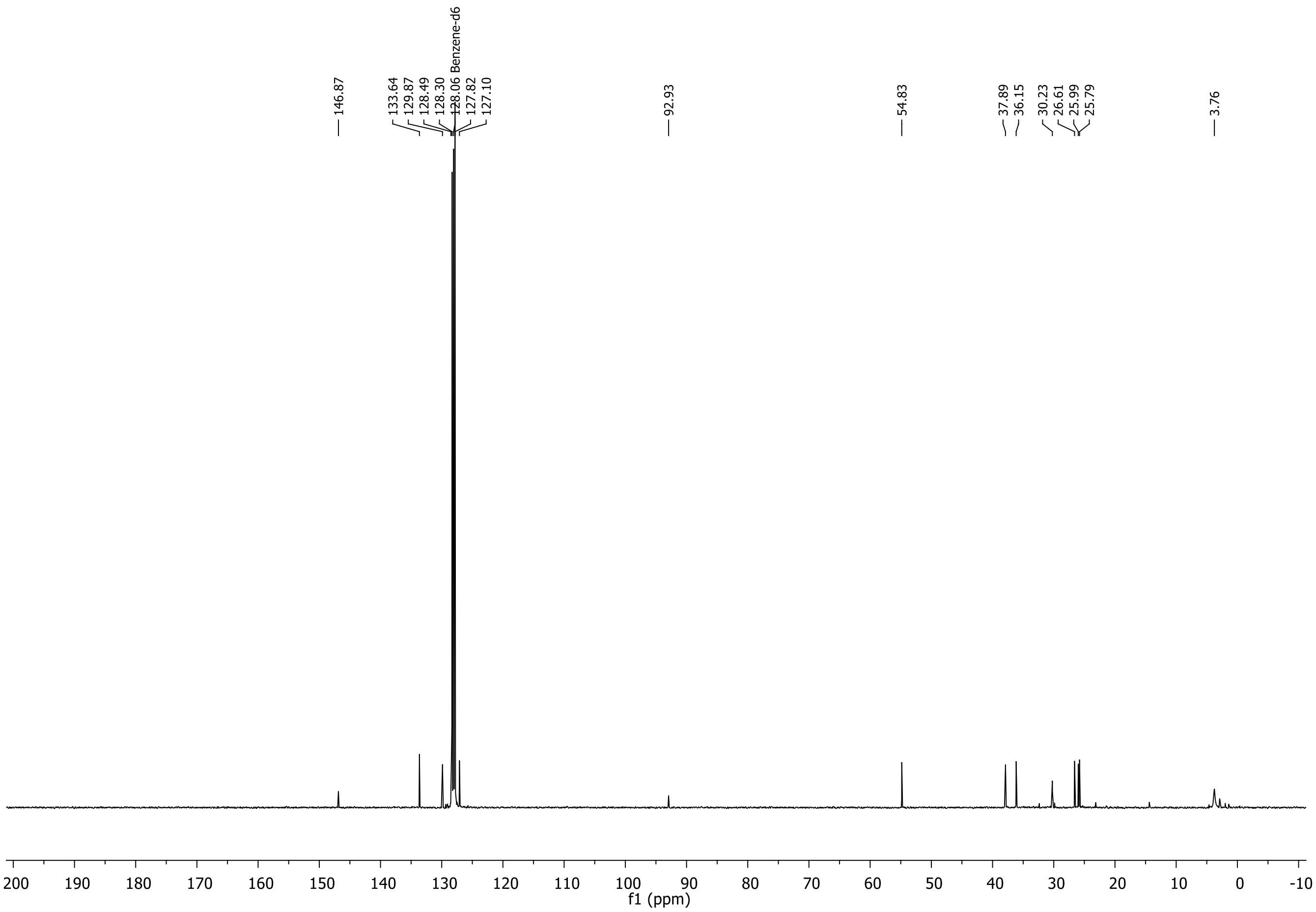

Figure S29. ^{13}C NMR spectrum of **12** (101 MHz, C_6D_6, 25 °C)

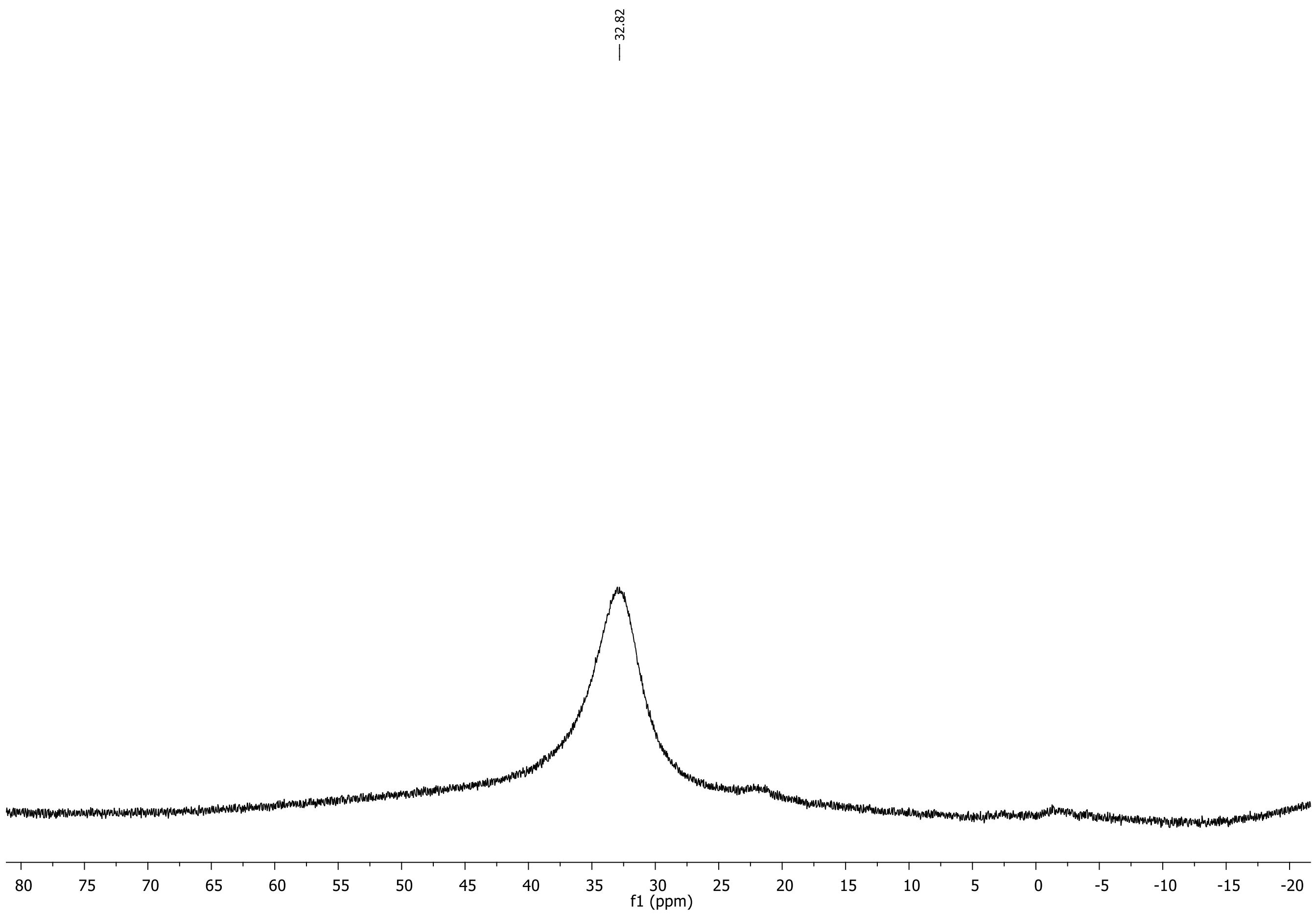

Figure S30. ^{11}B NMR spectrum of **12** (128 MHz, C_6D_6, 25 °C)

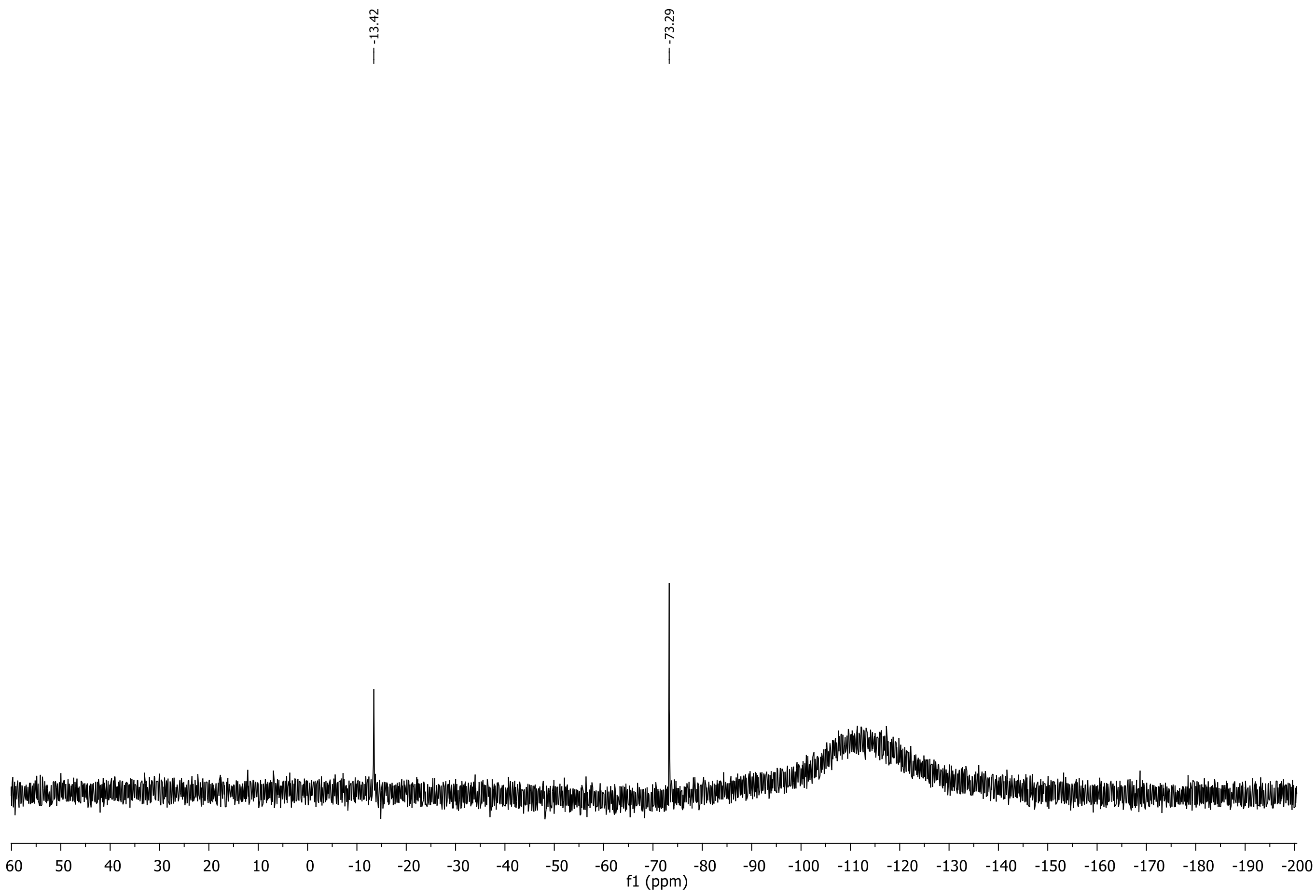

Figure S31. ^{29}Si NMR spectrum of **12** (99.36 MHz, C_6D_6, 25 °C)

Synthesis of 13. Alane *N*,*N*-dimethylethylamine complex solution in 0.5 M toluene (0.72 mL, 0.364 mmol) was added dropwise in the toluene solution of **3** (200 mg, 0.347 mmol in 10 mL of toluene) at –30 °C over a period of 10 min. Then, the reaction mixture was allowed to warm up to room temperature and stirred for 12 h. Subsequently, all volatiles were removed in vacuo and the residue was extracted into toluene (15 mL). The resultant toluene solution was concentrated to 5–8 mL and stored at –30 °C in a freezer, which afforded colorless crystals of **13** suitable for single crystal X-ray diffraction analysis within two days. Yield: 170 mg (90%). Mp: 162–164 °C.

13: ^{1}H NMR (400 MHz, C_6D_6, 25 °C): δ 7.88 (d, J = 6.7, 2H), 7.47 (dd, J = 7.1, 2.5, 2H), 7.41 (t, J = 7.6, 2H), 7.24 (t, J = 7.3, 1H), 7.06–6.98 (m, 3H), 3.33 (dd, J = 10.0, 6.6, 2H), 1.92 (d, J = 12.5, 2H), 1.75 (d, J = 12.2, 2H), 1.38 (d, J = 12.9, 4H), 1.22 (dd, J = 21.3, 10.7, 4H), 1.09–0.91 (m, 6H), 0.69 (d, J = 13.2, 2H), 0.53 (s, 27H) ppm. ^{13}C{^{1}H} NMR (101 MHz, C_6D_6, 25 °C): δ 164.6 (s), 134.2 (s), 132.4 (s), 130.3 (s), 128.9 (s), 128.5 (s), 127.1 (s), 127.0 (s), 56.1 (s), 36.1 (s), 34.4 (s), 26.2 (s), 25.8 (s), 25.7 (s), 4.4 (s) ppm. ^{11}B{^{1}H} NMR (128 MHz, C_6D_6, 25 °C): δ 26.6 (s, 1B, *B*(H) ppm. ^{29}Si{^{1}H} NMR (99.36 MHz, C_6D_6, 25 °C): δ –13.70 (*Si*(SiMe$_3$)), –70.16 (Si(*Si*Me$_3$)) ppm. ESI-HRMS: Calcd for $[M + H]^+$ $C_{28}H_{56}BN_2Si_4$: 543.3614, found: 543.3623. Elemental Analysis was unsuccessful.

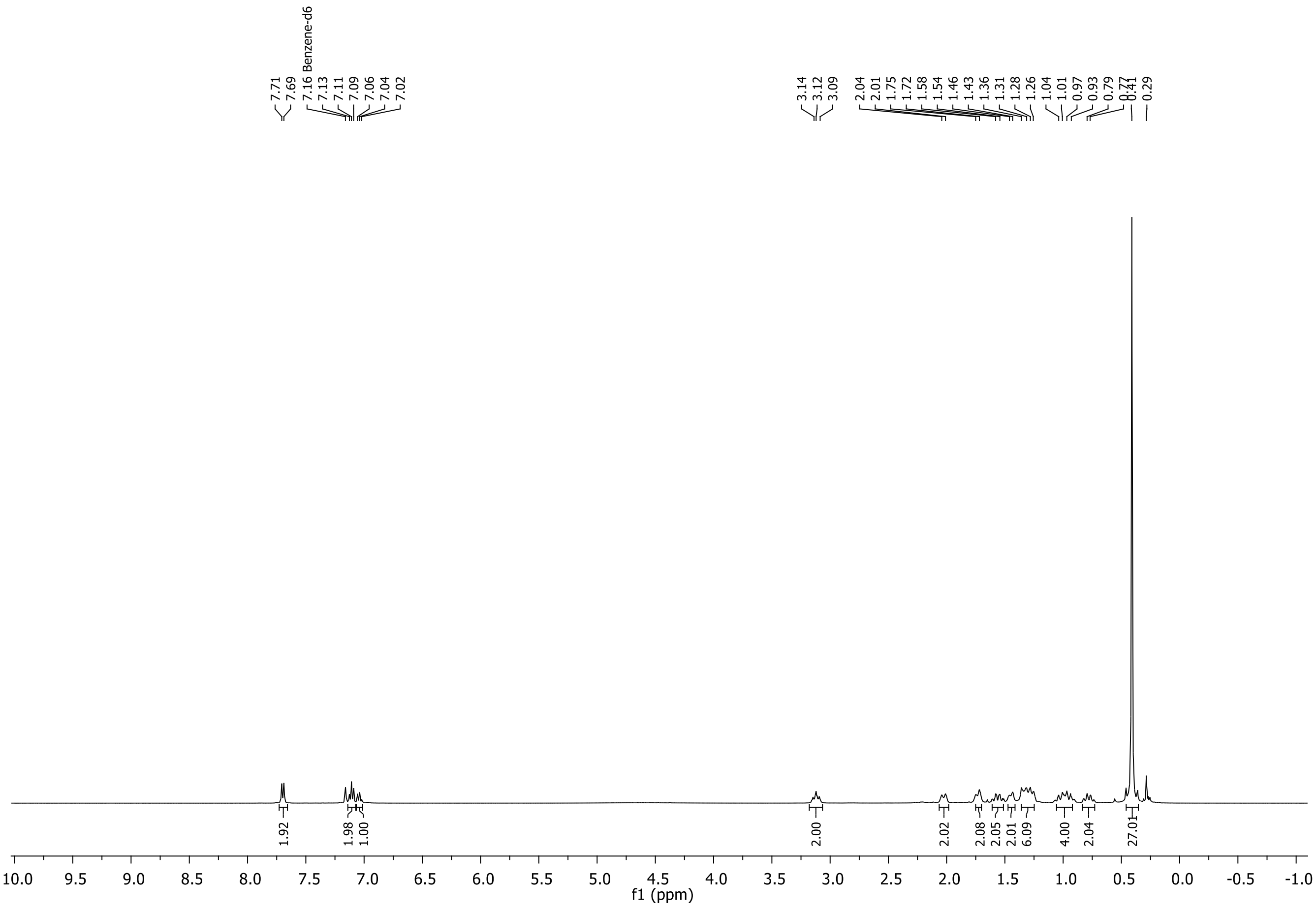

Figure S32. ^{1}H NMR spectrum of **13** (400 MHz, C_6D_6, 25 °C)

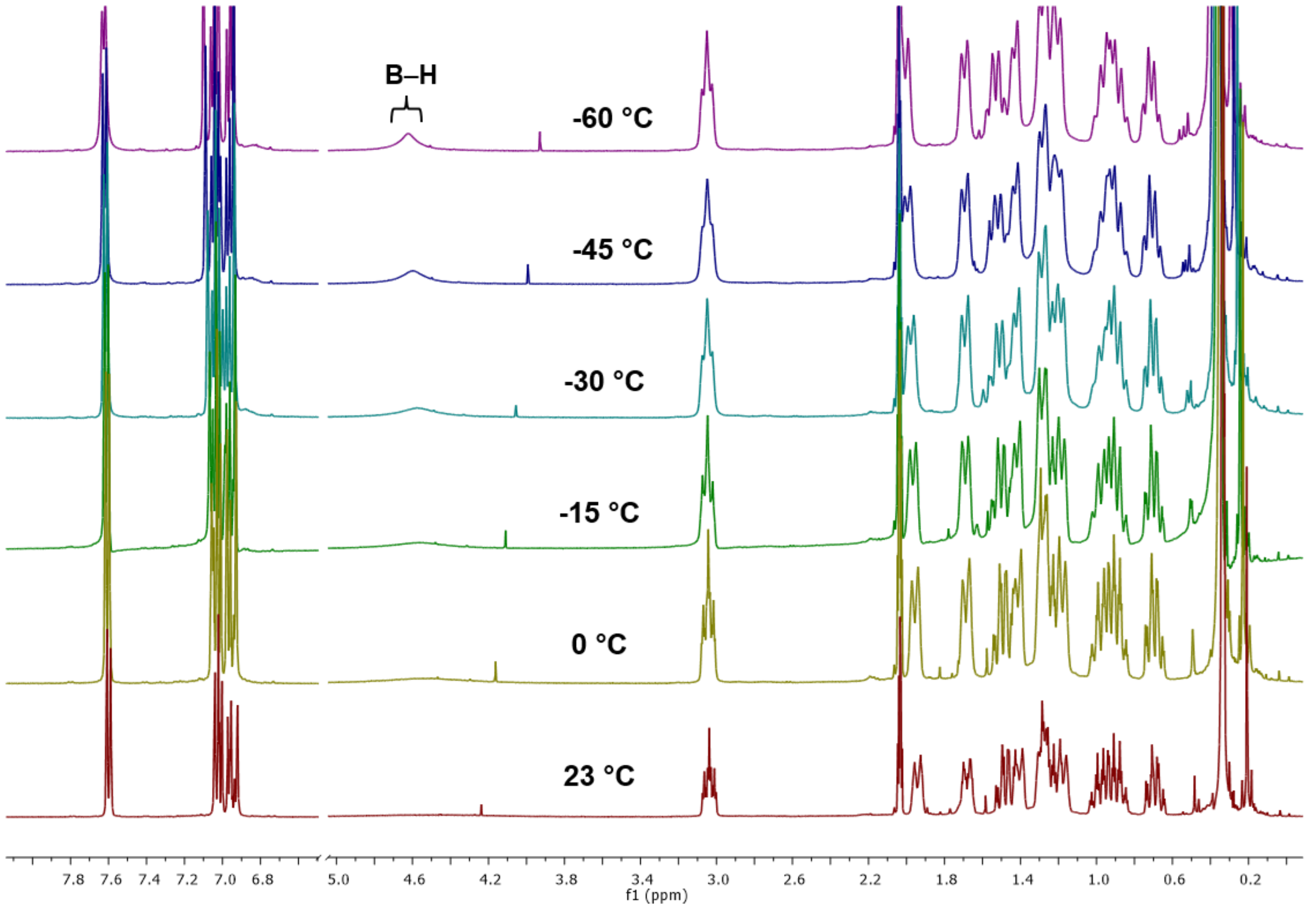

Figure S33. Variable temperature ^{1}H NMR spectra (400 MHz) of Ph(SiTMS$_3$)C(NCy)$_2$-BH (**13**) in toluene-d$_8$ (400 MHz, C_6D_6, 25 °C)

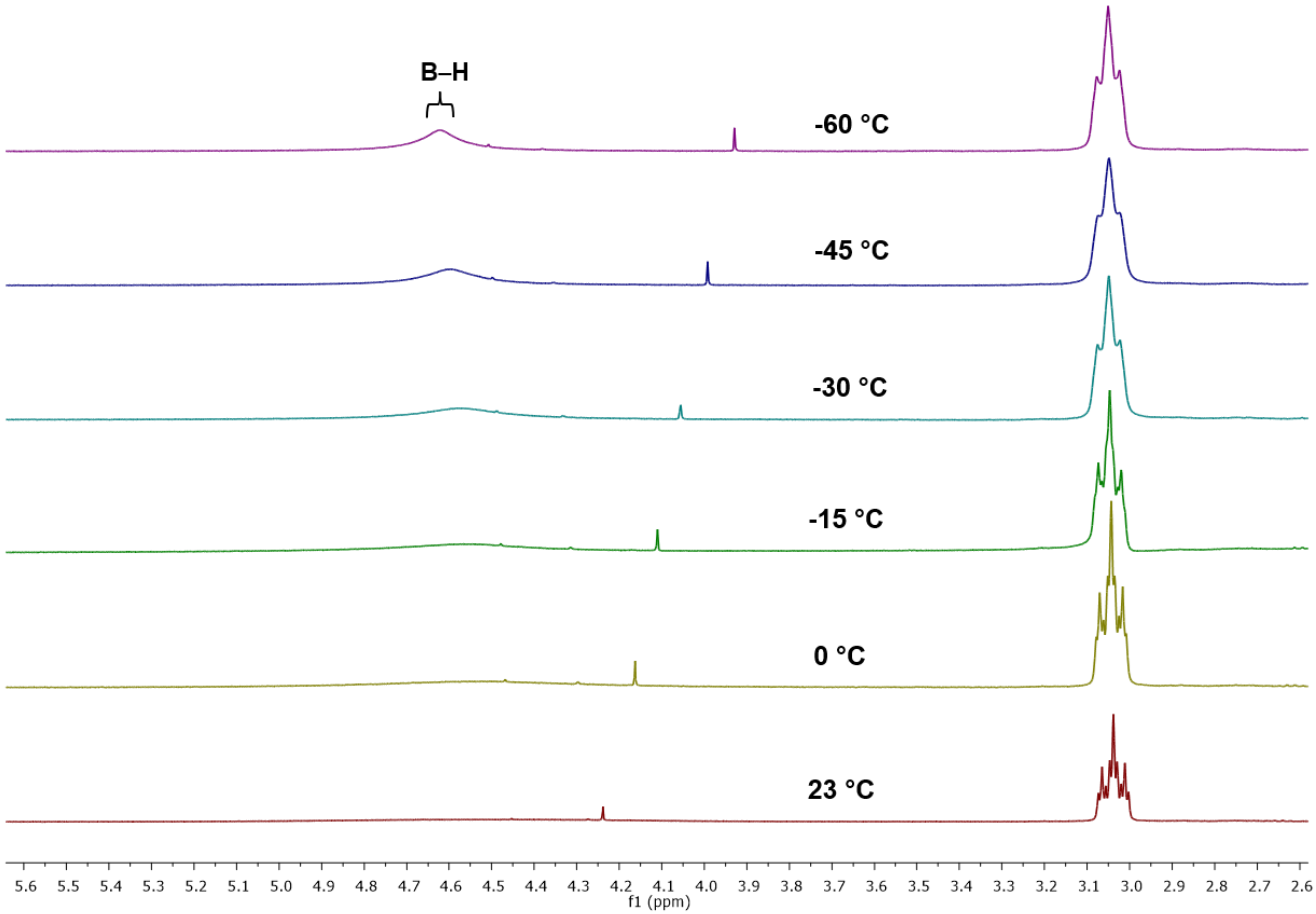

Figure S34. Variable temperature ^{1}H NMR spectra (400 MHz) of Ph(SiTMS$_3$)C(NCy)$_2$-BH (**13**) in toluene-d$_8$ (BH peak) (400 MHz, C_6D_6, 25 °C)

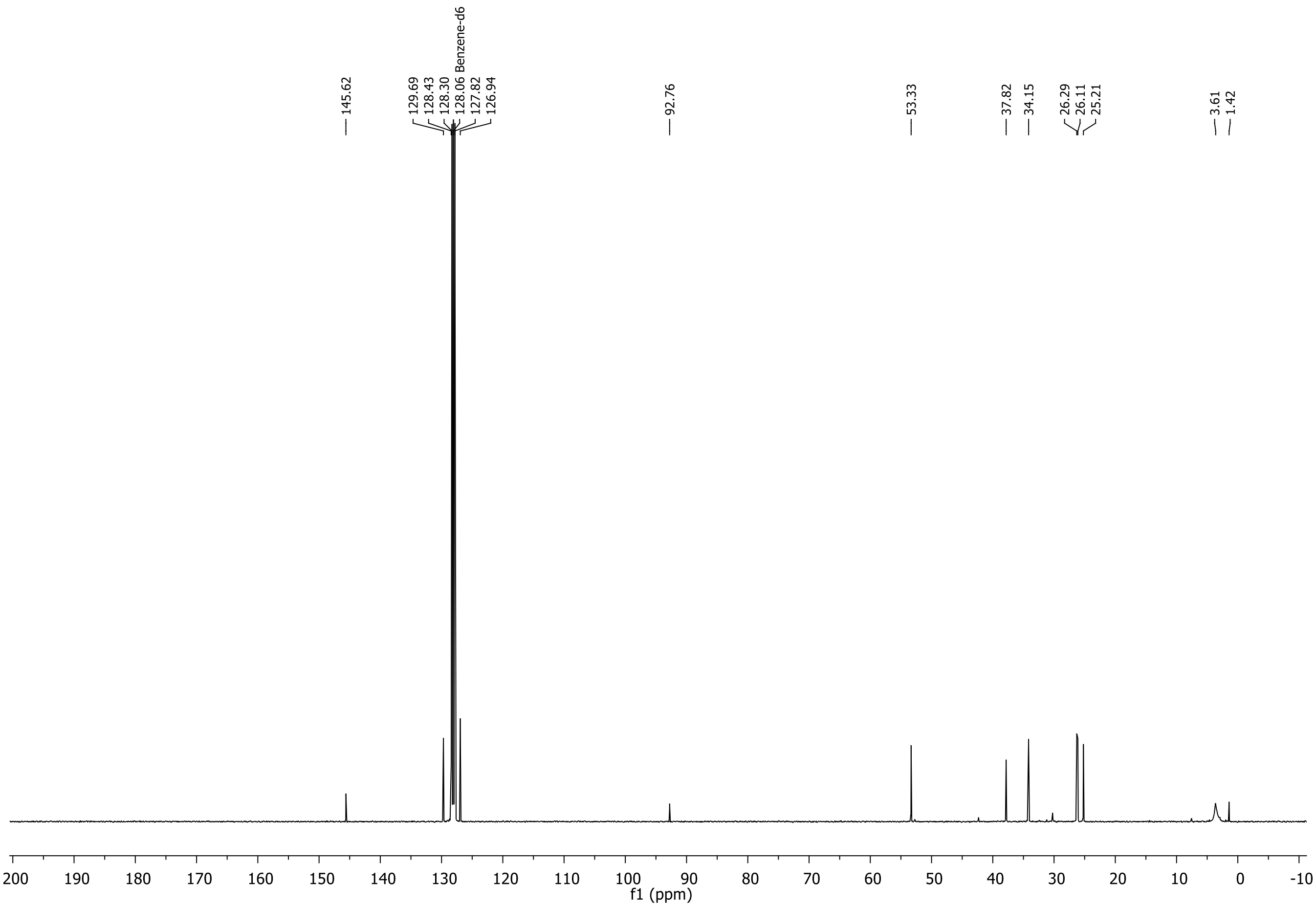

Figure S35.^{13}C NMR spectrum of **13** (101 MHz, C_6D_6, 25 °C)

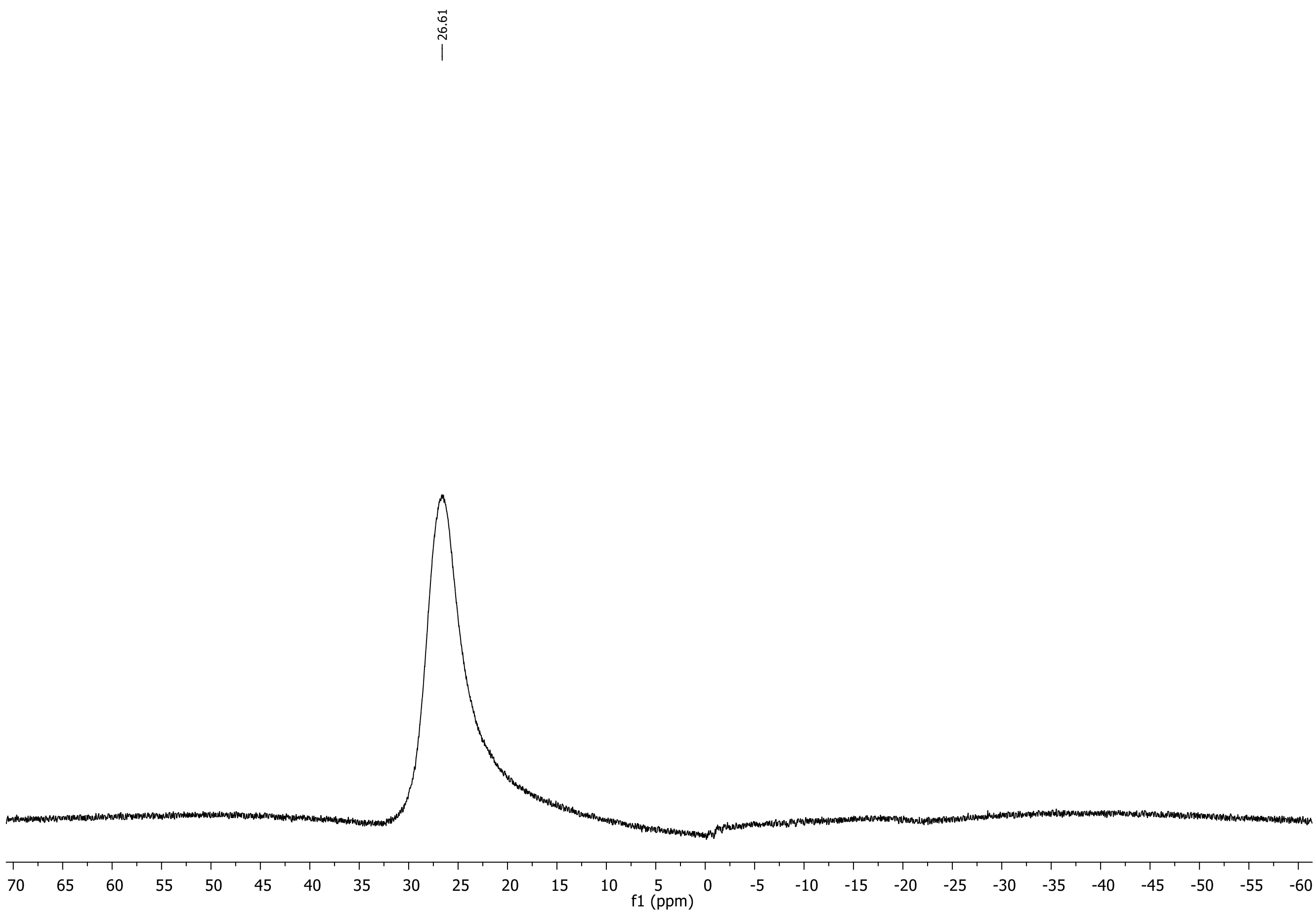

Figure S36. ^{11}B NMR spectrum of **13** (128 MHz, C_6D_6, 25 °C)

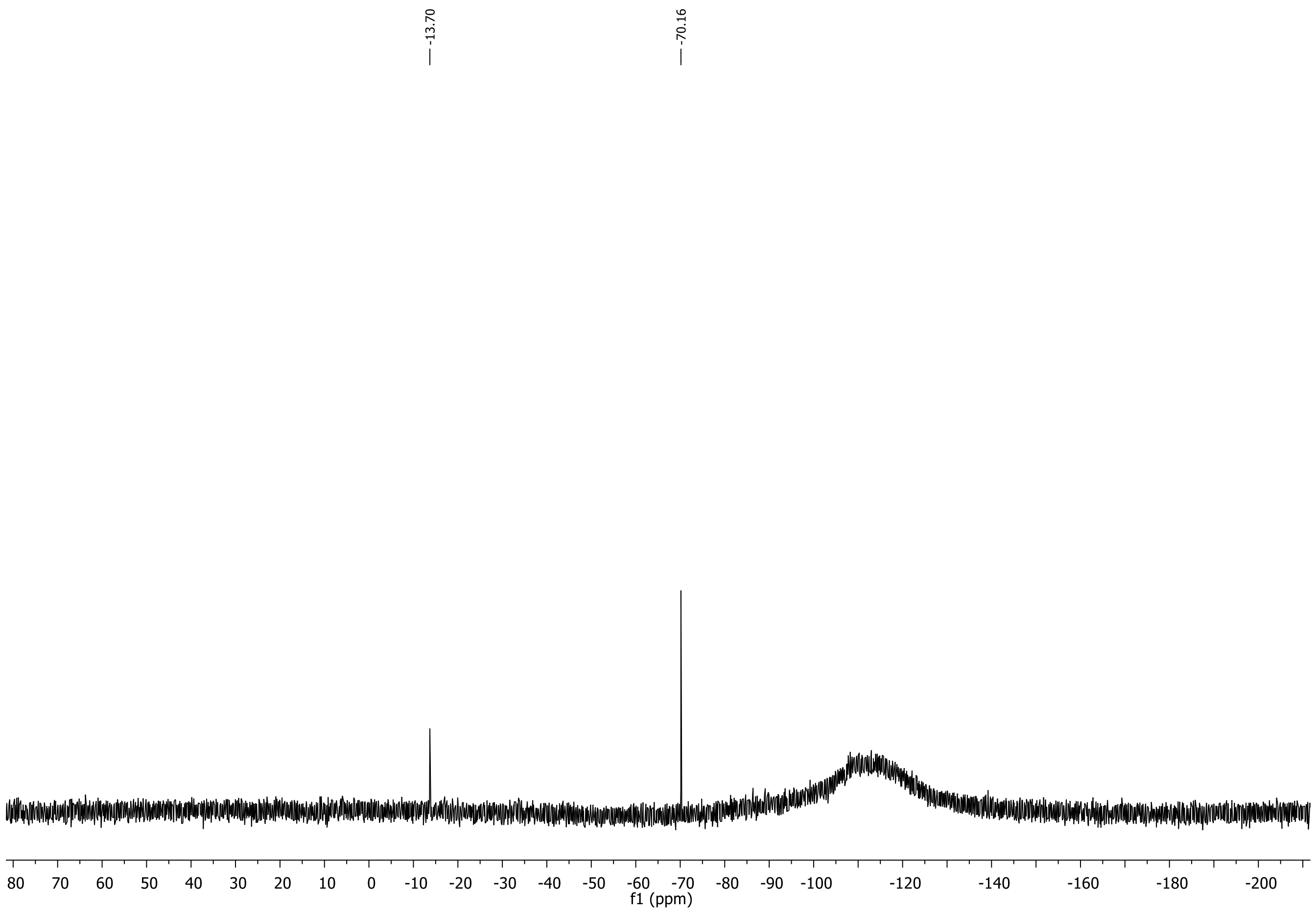

Figure S37. ^{29}Si NMR spectrum of **13** (99.36 MHz, C_6D_6, 25 °C)

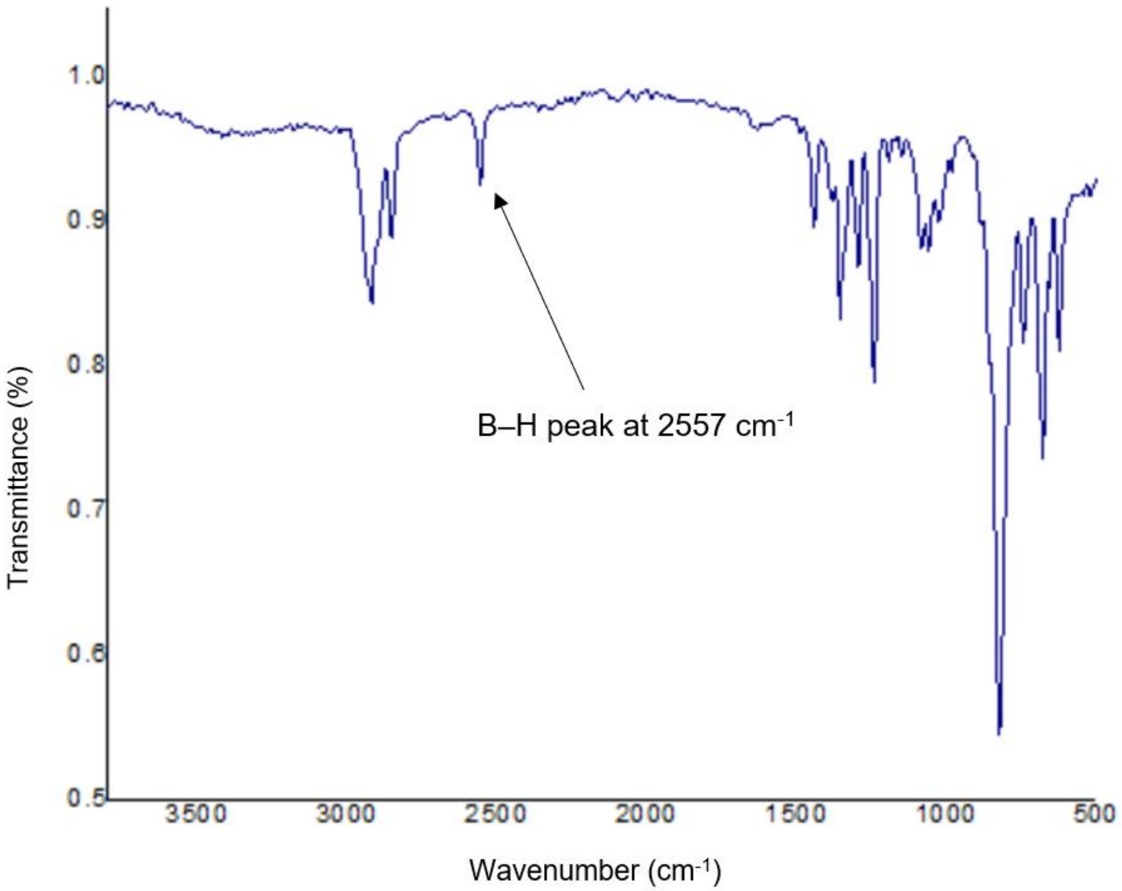

Figure S38. FT-IR spectrum of **13** (solid-state, 25 °C)

3. Thermal characterization data for compound 13

Thermal characterization data was measured using a double furnace PerkinElmer DSC 8000 coupled with PerkinElmer Intracooler II for temperature control. The system was calibrated with indium. Sample **13** were weighed and crimped in sample pans inside a glovebox. Hermetically sealable 50 μL aluminum sample pans were used, with a vented aluminum cover with a pierce hole of 0.05 mm diameter. The sample was heated from 30 °C to 250 °C, at a rate of 30 °C min^{-1}, under a nitrogen flow, followed by an isothermal hold at 250 °C for 1 min. The sample was then cooled to 30 °C at a rate of 30 °C min^{-1}, and kept at 30 °C for 1 min. DSC curves are shown with endotherm up.

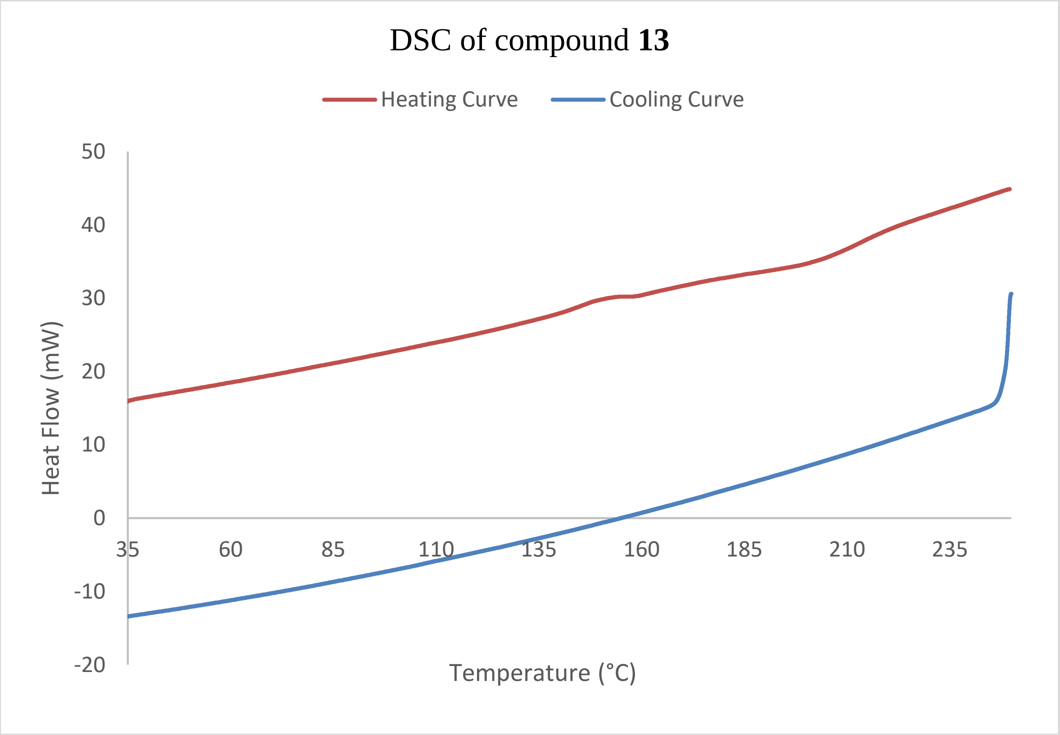

Figure S39. DSC heating and cooling curves for **13**, temperature range: 30 °C to 350 °C, sample mass: 2.4 mg

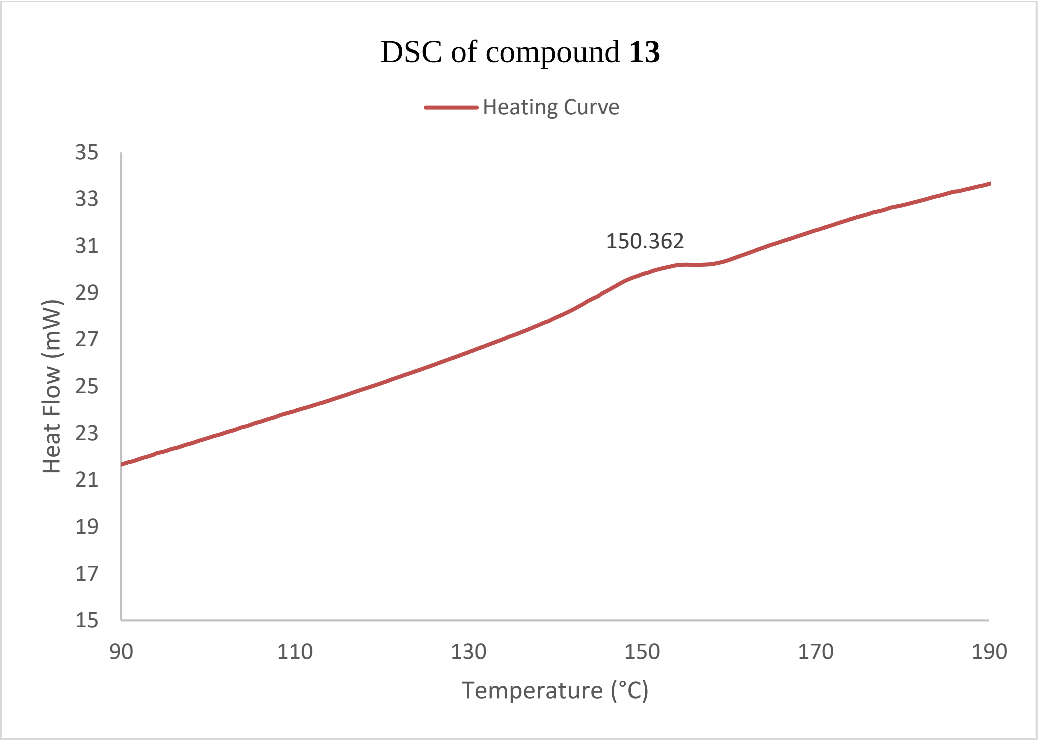

Figure S40. DSC heating curve for **13**, temperature data label for thermal event is shown above the curve in °C

4. Tentative mechanism for the formation of compound 11

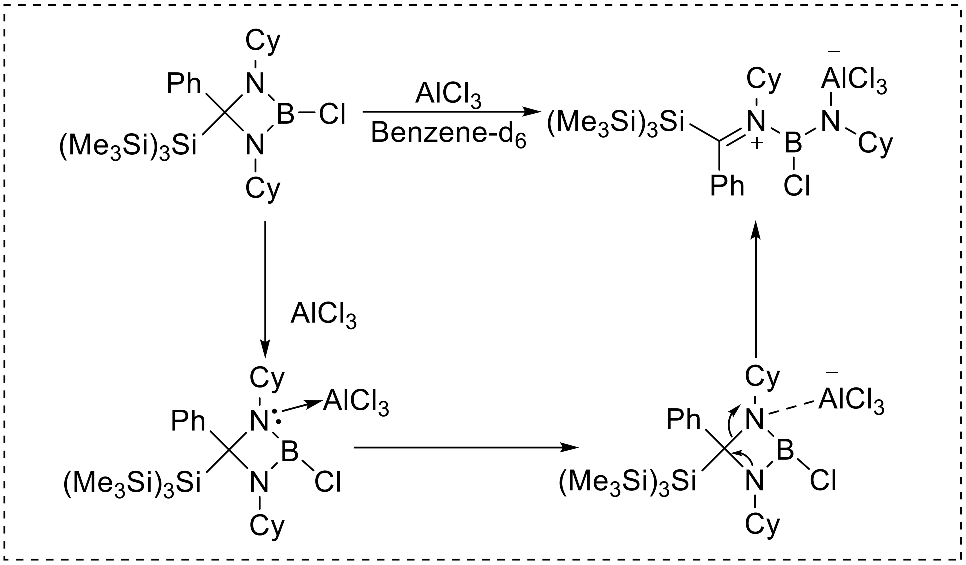

Scheme S1: Plausible reaction mechanism for the formation of compound **11**

5. Crystallographic data for the structural analysis of compounds **2–4**, **6–9**, **11–13**

Good quality single crystals of **2–4**, **6–9**, and **11–13** were hand-picked under polarized optical microscopy in Fomblin®Y and were undertaken on an Agilent SuperNova Dual Atlas four-circle diffractometer using Mo- or Cu-Kα radiation and a CCD detector. Measurements were taken at 200(2) K with temperatures maintained using an Oxford cryostream. Data were collected, integrated and corrected for absorption within CrysAlisPro.[S2] The absorption correction implemented a numerical absorption correction based on Gaussian integration over a multifaceted crystal model. The structures were solved by intrinsic phasing and refined against F^2 within SHELXL-2013.[S3] The structures have been deposited with the Cambridge Structural Database [CCDC accession numbers: 2313968–2313976, 2314978]. These can be obtained free of charge from the Cambridge Crystallographic Data Centre via www.ccdc.cam.ac.uk/data_request/cif. Crystals and refinement data are shown below.

**Identification code**	**2**	**3**	**4**	**6**	**7**
**Empirical formula**	$C_{25}H_{42}BClN_2$	$C_{28}H_{54}BN_2Si_4Cl$	$C_{34}H_{59}BN_2Si_4$	$C_{22}H_{46}BN_2Si_4Cl$	$C_{17}H_{31}BN_2Cl_2$
**Formula weight**	203.39	577.35	619.00	497.23	345.15
**Temperature/K**	200(2)	200(2)	200(2)	200(2)	200(2)
**Crystal system**	monoclinic	monoclinic	triclinic	triclinic	triclinic
**Space group**	$P2_1/c$	$P2_1/c$	*P*-1	*P*-1	*P*-1
**a/Å**	9.6768(4)	11.5068(2)	9.9541(4)	10.2419(3)	8.9706(6)
**b/Å**	12.0527(5)	16.3487(3)	11.0420(4)	15.5240(6)	9.8842(6)
**c/Å**	20.0278(10)	18.4759(3)	17.2595(7)	19.4256(6)	12.0879(8)
**α/°**	90	90	87.845(3)	88.752(3)	101.491(5)
**β/°**	101.724(4)	91.537(2)	81.933(3)	76.881(3)	92.790(5)
**γ/°**	90	90	85.737(3)	83.525(3)	114.859(6)
**Volume/Å^3**	2287.14(18)	3474.46(10)	1872.37(13)	2988.76(18)	942.37(11)
**Z**	8	4	2	4	2
**ρ_{calc}g/cm^3**	1.181	1.104	1.098	1.105	1.216
**μ/mm^{-1}**	0.180	2.425	1.641	0.301	3.060
**F(000)**	872	1256	676	1080	372
**Crystal size/mm^3**	0.46 × 0.27 × 0.22	0.31 × 0.24 × 0.17	0.302 × 0.207 × 0.051	0.407 × 0.341 × 0.164	0.308 × 0.196 × 0.161
**c**	SuperNova, Dual, Cu at home/near, Atlas				
**Absorption correction**	Gaussian				
**T_{min}, T_{max}**	0.732, 1.000	0.359, 1.000	0.424, 1.000	0.410, 1.000	0.506, 1.000
**No. of measured, independent and observed [I > 2σ(I)] reflections**	12763, 5481, 4247	13421, 6732, 5461	13185, 7228, 6078	27110, 14103, 11081	6532, 3635, 3062
**Reflections collected**	5481	6732	7228	14103	3635
R_{int}	0.0441	0.0538	0.0456	0.0404	0.0363
**No. of parameters**	262	334	379	567	202
**Goodness-of-fit on F^2**	1.018	1.035	1.056	1.062	1.036
**Final R indexes [I>=2σ (I)]**	R_1 = 0.0441, wR_2 = 0.0904	R_1 = 0.0538, wR_2 = 0.1397	R_1 = 0.0456, wR_2 = 0.1231	R_1 = 0.0404, wR_2 = 0.0890	R_1 = 0.0363, wR_2 = 0.0908
**Final R indexes [all data]**	R_1 = 0.0625, wR_2 = 0.1023	R_1 =0.0669, wR_2 = 0.1566	R_1 = 0.0549, wR_2 = 0.1313	R_1 = 0.0576, wR_2 = 0.1023	R_1 = 0.0452, wR_2 = 0.0987
**Largest diff. peak/hole / e Å^{-3}**	0.26/-0.23	0.52/-0.29	0.98/-0.33	0.31/-0.24	0.23/-0.26
**CCDC #**	2313968	2313972	2313976	2314978	2313973

Identification code	8	9	11	12	13
Empirical formula	$C_{26}H_{58}BN_2Si_4Cl$	$C_{25}H_{39}BCl_2N_2$	$C_{28}H_{54}BN_2AlSi_4Cl_4$	$C_{34}H_{59}BN_2Si_4$	$C_{28}H_{55}BN_2Si_4$
Formula weight	557.36	449.29	710.68	618.38	542.35
Temperature/K	200(2)	200(2)	200(2)	200(2)	200(2)
Crystal system	monoclinic	monoclinic	triclinic	triclinic	triclinic
Space group	$P2_1/c$	C*c*	*P*-1	*P*-1	*P*-1
a/Å	10.0545(2)	17.0795(17)	9.7525(4)	11.2265(2)	9.9837(4)
b/Å	19.6449(4)	10.1510(8)	11.7311(5)	19.8071(6)	10.4208(5)
c/Å	17.2449(4)	16.8516(18)	18.3546(7)	27.1109(6)	16.6171(9)
α/°	90	90	106.992(4)	82.355(2)	80.386(4)
β/°	95.799(2)	116.512(13)	99.806(3)	81.616(2)	89.385(4)
γ/°	90	90	93.705(3)	74.447(2)	79.760(4)
Volume/Å^3	3388.78(13)	2614.4(5)	1964.26(15)	5717.7(2)	1677.06(14)
Z	4	4	2	14	4
ρ_{calc}g/cm^3	1.092	1.141	1.202	1.079	1.075
μ/mm^{-1}	2.465	0.262	4.276	0.180	0.196
F(000)	1224	968	756	2028	596
Crystal size/mm^3	0.334 × 0.174 × 0.13	0.611 × 0.327 × 0.235	0.281 × 0.141 × 0.048	0.523 × 0.492 × 0.258	0.774 × 0.303 × 0.221
Diffractometer	SuperNova, Dual, Cu at home/near, Atlas				
Absorption correction	Gaussian				
T_{min}, T_{max}	0.620, 1.000	0.483, 1.000	0.600, 1.000	0.490, 1.000	0.519, 1.000
No. of measured, independent and observed [I > 2σ(I)] reflections	14320, 6605, 5349	6934, 4580, 3969	14342, 7605, 6641	49991, 26739, 19084	15235, 7948, 6564
Reflections collected	6605	4580	7605	26739	7948
R_{int}	0.0479	0.0960	0.0370	0.0539	0.0940
No. of parameters	319	275	370	1135	328
Goodness-of-fit on F^2	1.037	1.052	1.036	1.070	1.137
Final R indexes [I>=2σ (I)]	R_1 = 0.0479, wR_2 = 0.1239	R_1 = 0.0960, wR_2 = 0.2678	R_1 = 0.0370, wR_2 = 0.0951	R_1 = 0.0539, wR_2 = 0.1182	R_1 = 0.0940, wR_2 = 0.2311
Final R indexes [all data]	R_1 = 0.0607, wR_2 = 0.1354	R_1 =0.1029, wR_2 = 0.2798	R_1 = 0.0434, wR_2 = 0.1011	R_1 = 0.0825, wR_2 = 0.1347	R_1 = 0.1082, wR_2 = 0.2383
Largest diff. peak/hole / e Å^{-3}	0.46/-0.54	1.37/-0.37	0.49/-0.47	0.45/-0.66	0.97/-0.38
CCDC No	2313975	2313969	2313971	2313974	2313970

Crystal structure of compounds 2–4, 6–9 and 11–13 with thermal ellipsoids

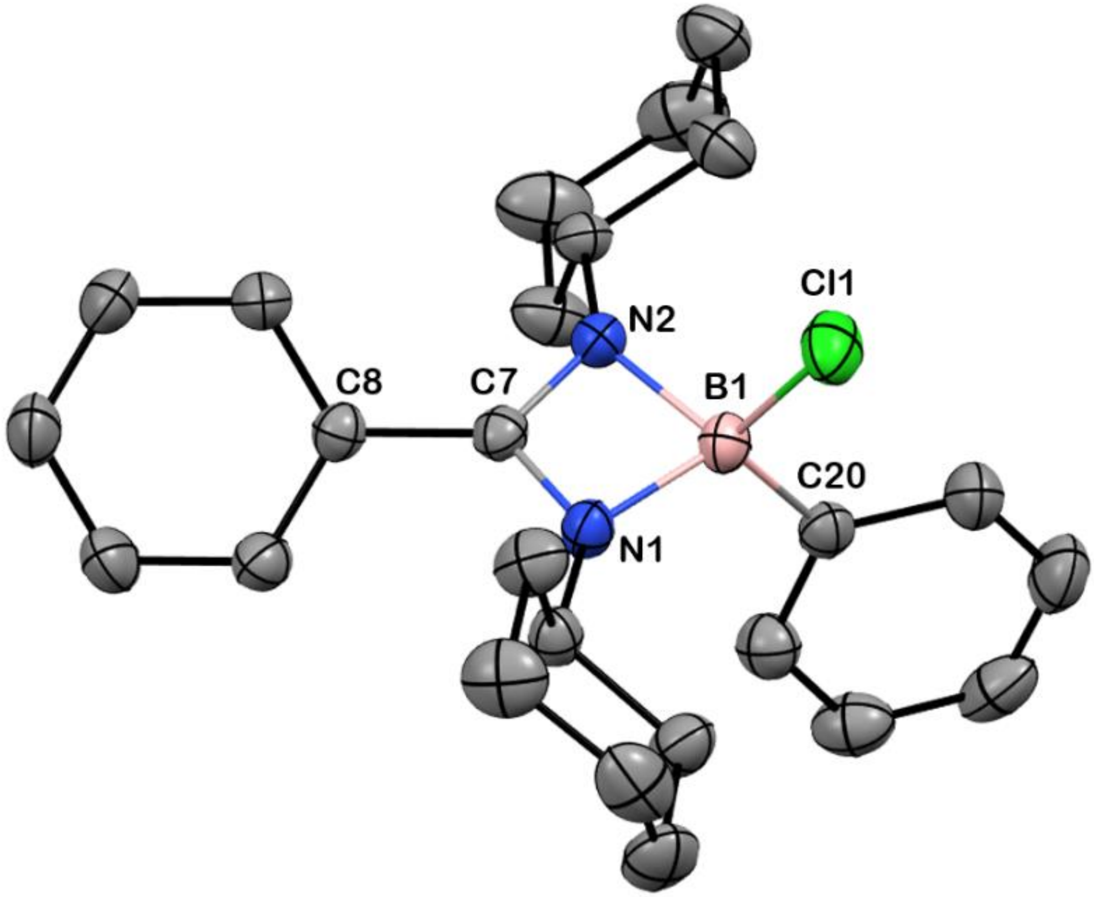

Figure S41. Solid-state structure of **2**. Anisotropic displacement parameters are drawn at 50% probability. B is shown in pink, C in grey, N in blue, Cl in green; H atoms are omitted for clarity.

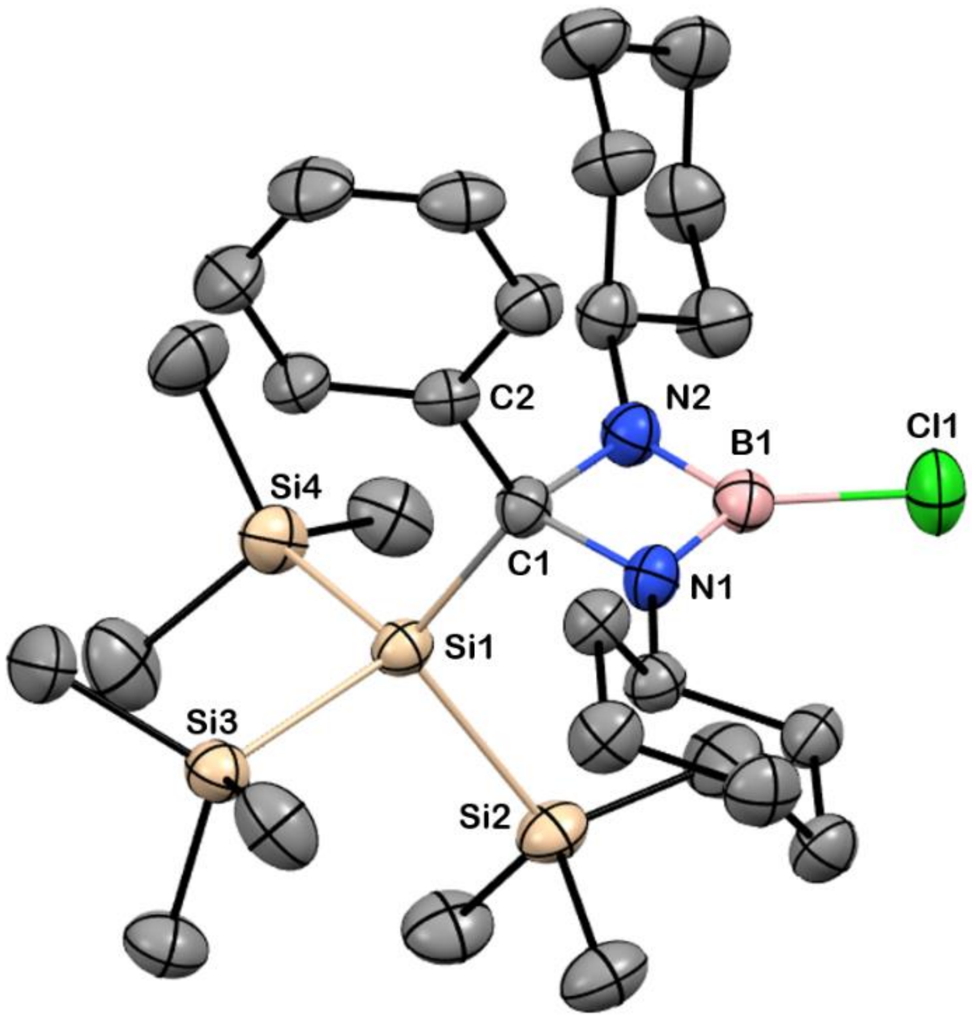

Figure S42. Solid-state structures of **3**. Anisotropic displacement parameters are depicted at the 50% probability level. B is shown in pink, C in grey, N in blue, Cl in green, Si in beige; H atoms are omitted for clarity.

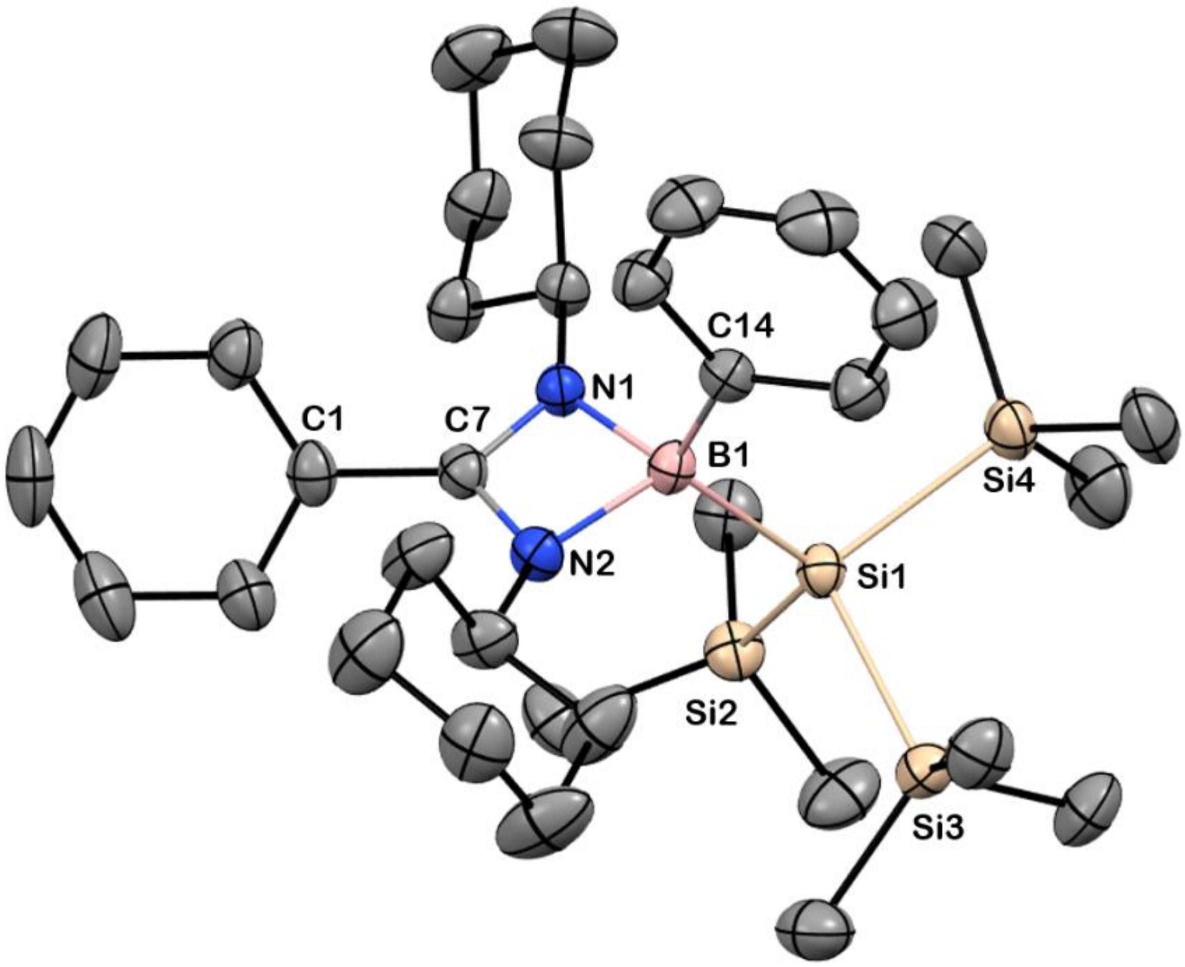

Figure S43. Solid-state structures of **4**. Anisotropic displacement parameters are depicted at the 50% probability level. B is shown in pink, C in grey, N in blue, Si in beige; H atoms are omitted for clarity.

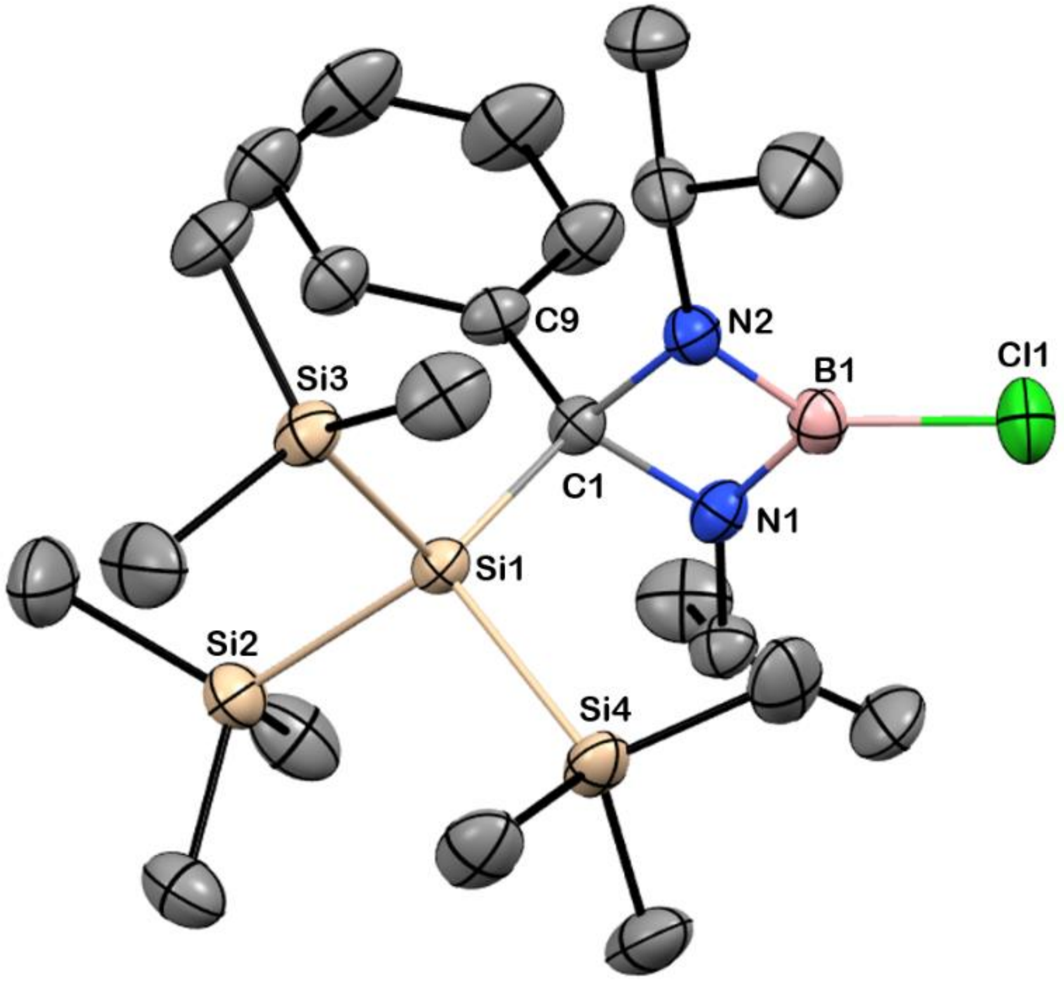

Figure S44. Solid-state structures of **6**. Anisotropic displacement parameters are depicted at the 50% probability level. B is shown in pink, C in grey, N in blue, Cl in green, Si in beige; H atoms are omitted for clarity.

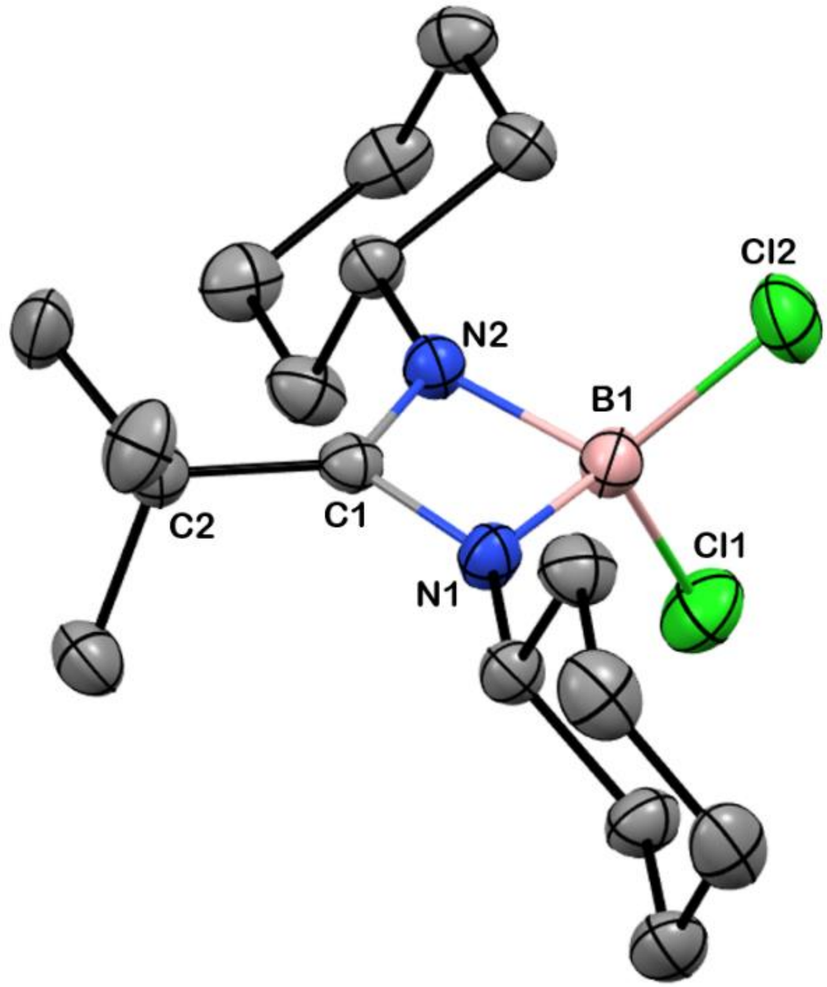

Figure S45. Solid-state structures of **7**. Anisotropic displacement parameters are depicted at the 50% probability level. B is shown in pink, C in grey, N in blue, Cl in green; H atoms are omitted for clarity.

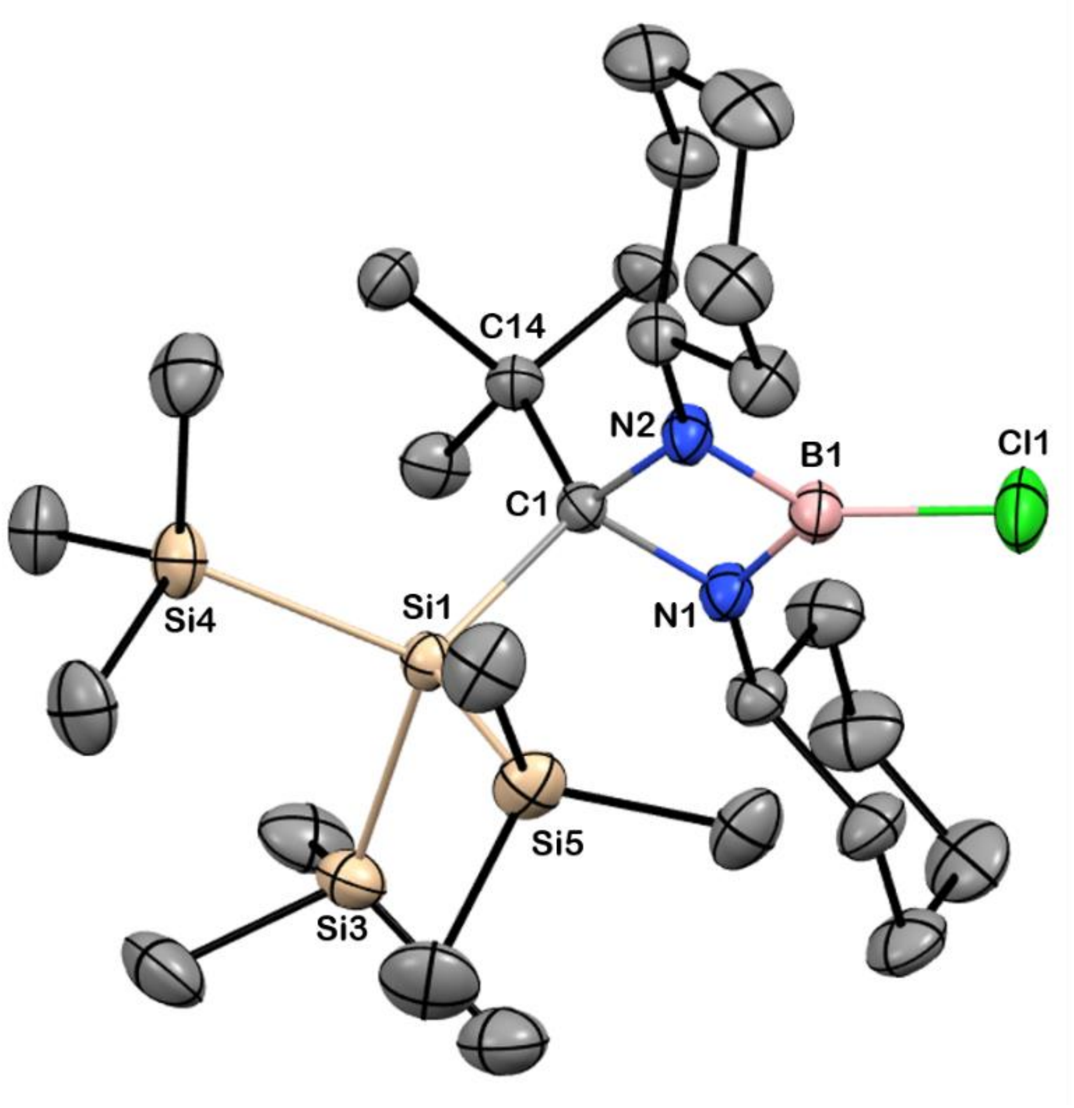

Figure S46. Solid-state structures of **8**. Anisotropic displacement parameters are depicted at the 50% probability level. B is shown in pink, C in grey, N in blue, Cl in green, Si in beige; H atoms are omitted for clarity.

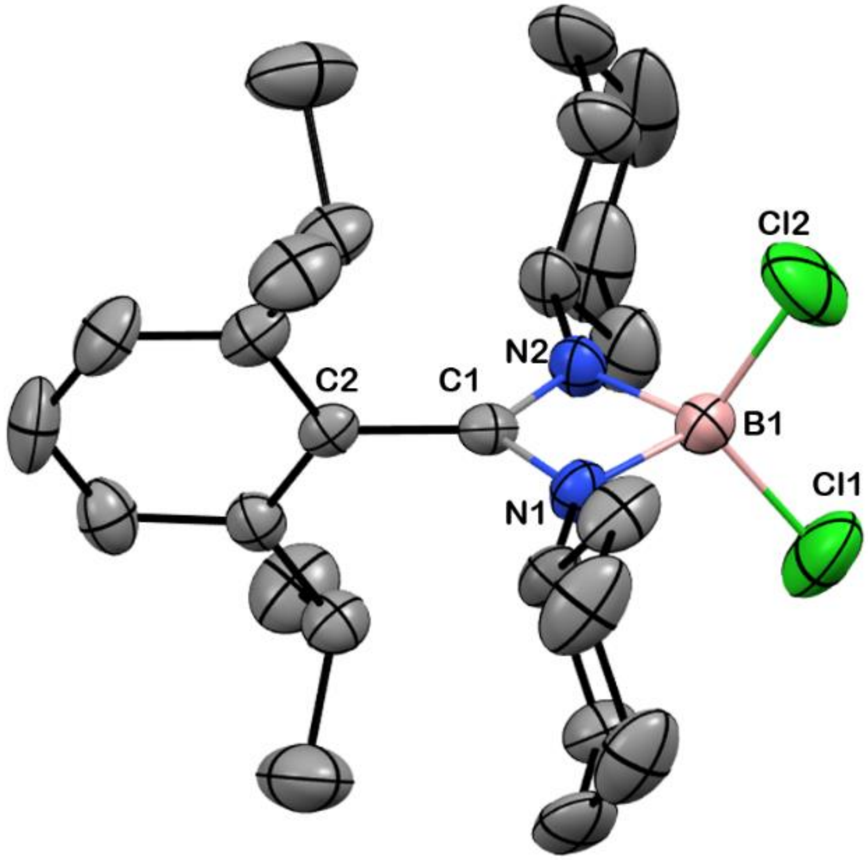

Figure S47. Solid-state structures of **9**. Anisotropic displacement parameters are depicted at the 50% probability level. B is shown in pink, C in grey, N in blue, Cl in green; H atoms are omitted for clarity.

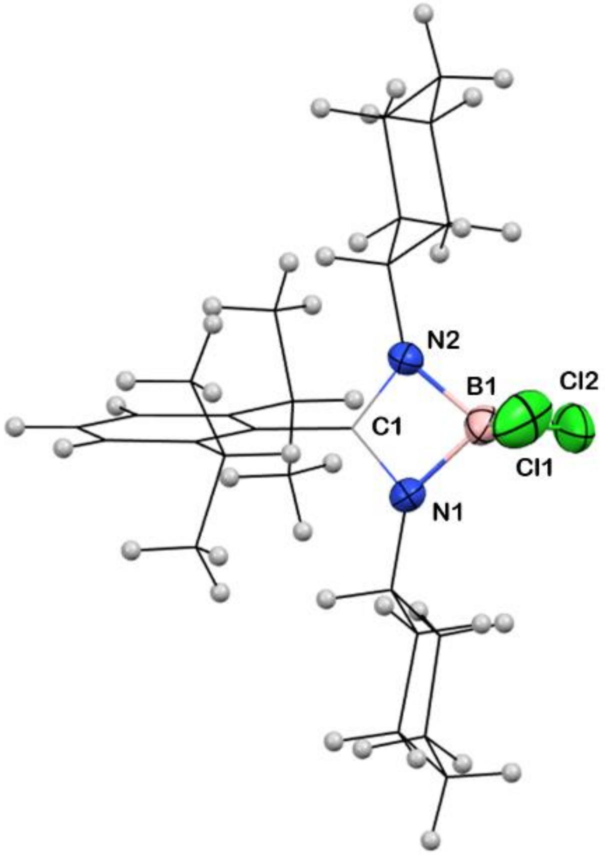

Figure S48. Solid-state structure of compound **9**. Anisotropic displacement parameters are depicted at the 50% probability level. B is shown in pink, C in grey, N in blue, Cl in green; *Cy* and *ph* groups have been drawn as wireframe for clarity. Hydrogen atoms are shown to illustrate the alignment of the isopropyl group of the Dipp (Dipp= 2,6-diisopropylphenyl) and cyclohexyl group to block the backbone of the amidinate ligand.

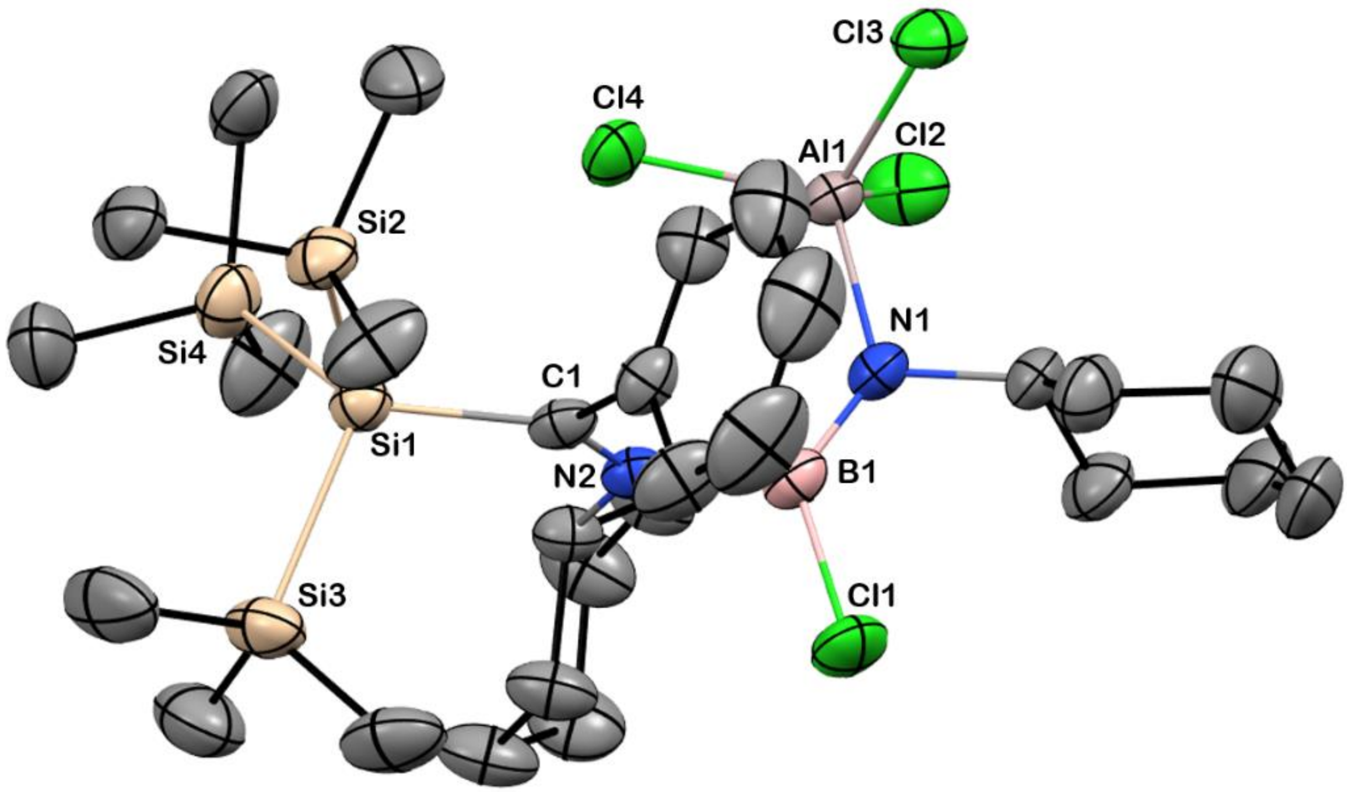

Figure S49. Solid-state structures of **11**. Anisotropic displacement parameters are depicted at the 50% probability level. B is shown in pink, C in grey, N in blue, Cl in green, Si in beige, Al in rosybrown; H atoms are omitted for clarity.

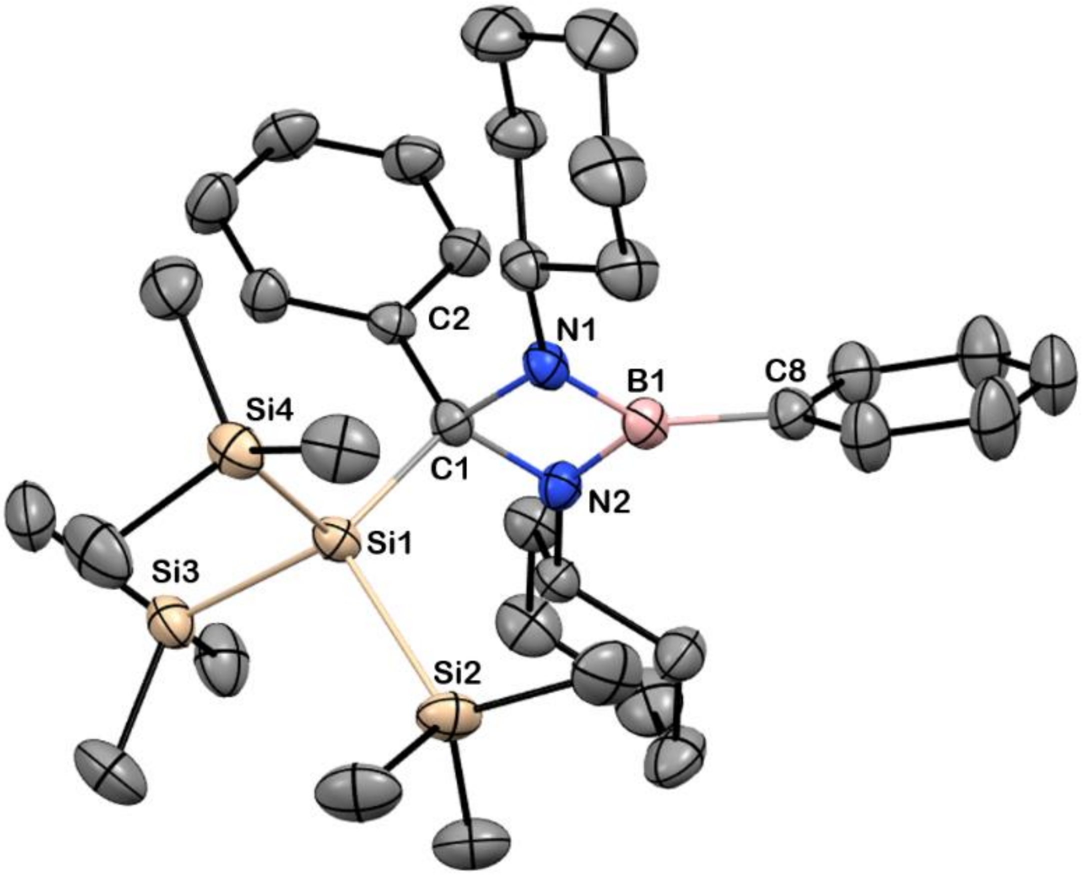

Figure S50. Solid-state structures of **12**. Anisotropic displacement parameters are depicted at the 50% probability level. B is shown in pink, C in grey, N in blue, Si in beige; H atoms are omitted for clarity.

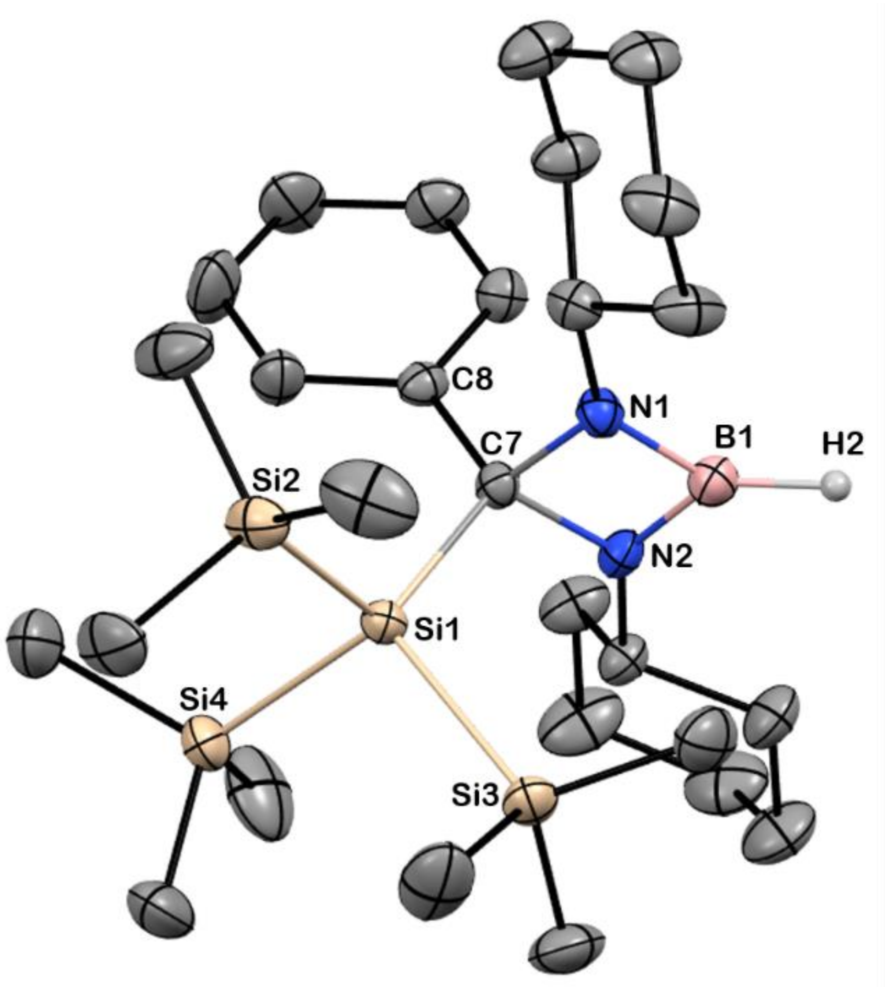

Figure S51. Solid-state structure of **13**. Anisotropic displacement parameters are depicted at the 50% probability level. Hydrogen atoms (except the $B{-}H$) are omitted for clarity. B is shown in pink, C in grey, N in blue, Si in beige, H in white.

6. Solid-state phase transformation details of compound 3

We have observed a phase transition whilst measuring the crystal of compound **3** and we investigated this change further with variable temperature (120 K–290 K) crystallographic studies. The investigation revealed rather complex behavior associated with the phase transformation which is illustrated with results from three crystals (crystals C1–C3) of compound **3**. Transition is recorded from the high-temperature (HI) form to the low-temperature (LT) form. The transformation occurs in a single crystal-to-single crystal manner, thereby enabling the structure of form LT to be determined. Both HT and LT forms are monoclinic, $P2_1/c$ (Table S1) with the same asymmetric unit (Figure S52). The transformation from HT to LT on cooling does not involve major changes in molecular conformation, as illustrated by the overlay of the molecules from the two structures (Figure S53). The process mainly involves slight adjustments in the orientations and positions of the molecules in the solid, as the similarity in crystal packing shows (Figures S54a and S55a). Associated with the transformation are modifications in intermolecular contacts. For example, a different geometry is observed in the two structures for C–H···Cl contacts (Table S2 and Figures S54b, S55b).

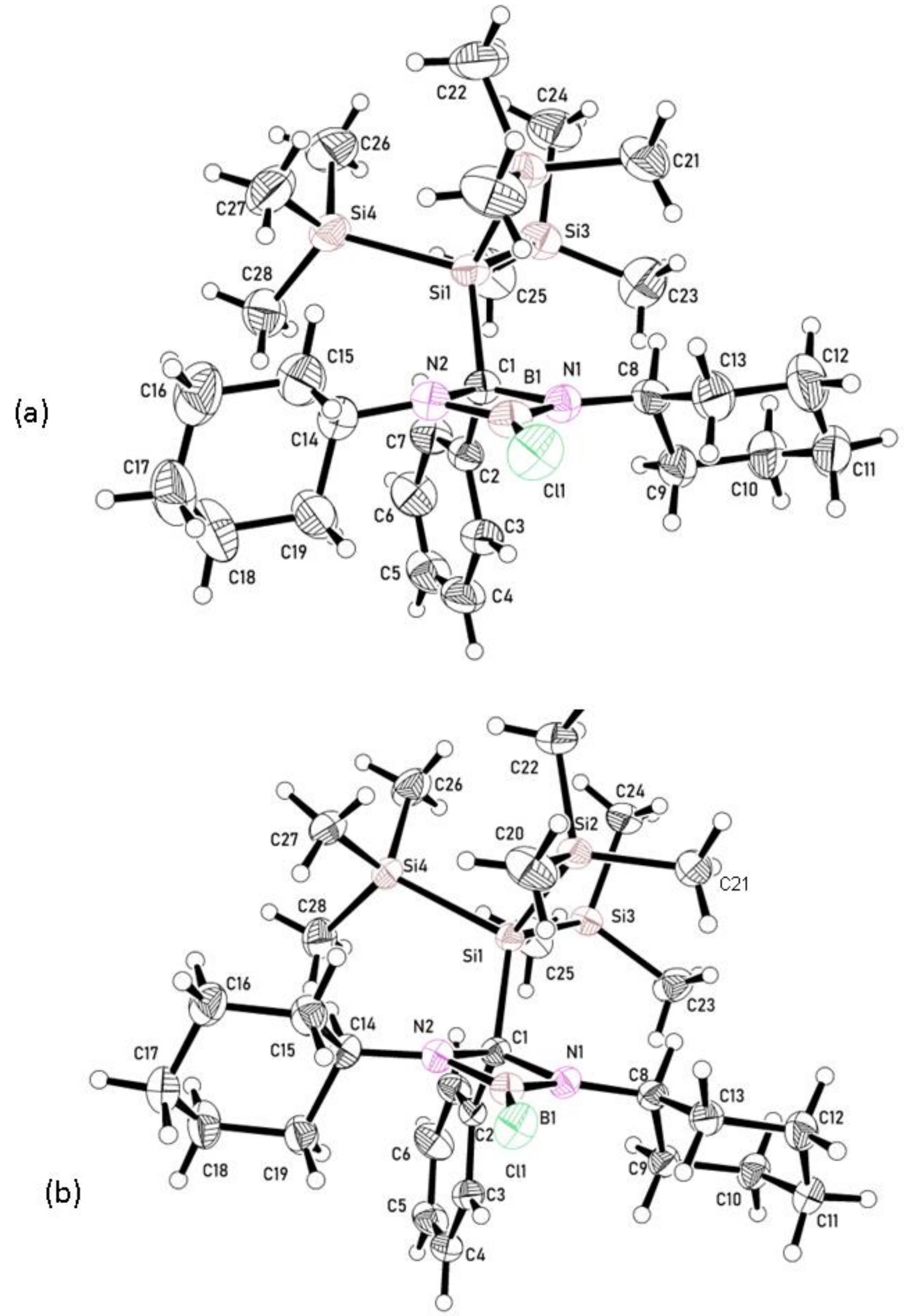

Figure S52. ORTEP representation of (a) HT and (b) LT forms showing 50% atomic displacement parameters (HT and LT from crystals C2 and C3 respectively)

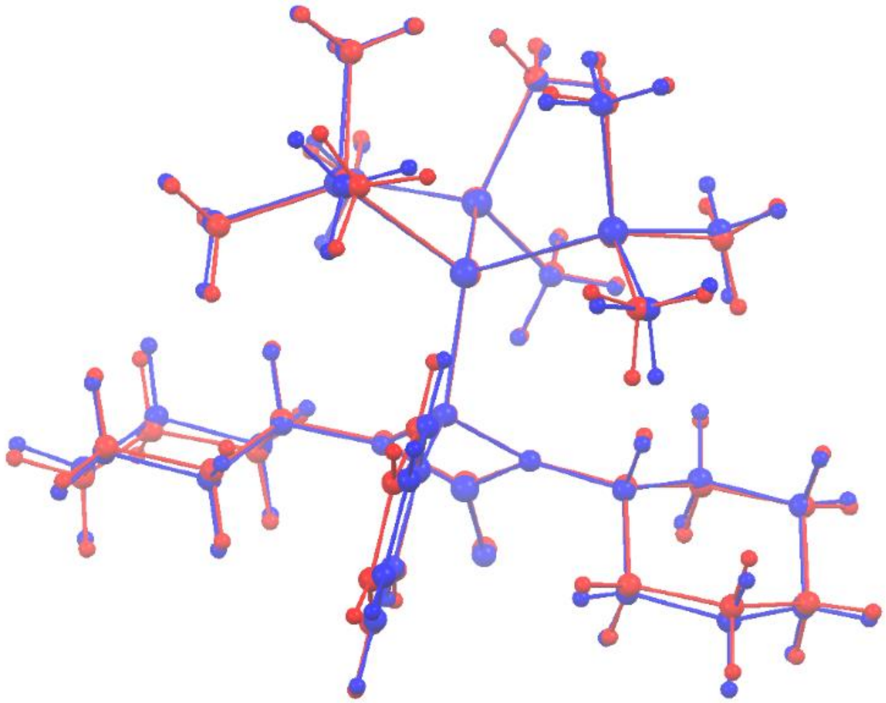

Figure S53. Overlay of the molecules of HT (red) and LT (blue) forms

Table S1. Crystal and structure refinement data (HT and LT from crystals C2 and C3 respectively)

	**HT**	**LT**
**Molecular formula**	$C_{28}H_{54}BClN_2Si_4$	$C_{28}H_{54}BClN_2Si_4$
**Formula weight**	577.35	577.35
**Temperature (K)**	290(2)	200(2)
**Wavelength (Å)**	1.54184	1.54184
**Crystal system**	Monoclinic	Monoclinic
**Space group**	$P2_1/c$	$P2_1/c$
**a (Å)**	12.1787(2)	11.5068(2)
**b (Å)**	16.5126(2)	16.3487(3)
**c (Å)**	17.3463(2)	18.4759(3)
**α (°)**	90	90
**β (°)**	92.6310(10)	91.537(2)
**γ (°)**	90	90
**Volume ($Å^3$)**	3484.70(8)	3474.46(10)
**Z**	4	4
**Density (calculated) (Mg/m^3)**	1.100	1.104
**Absorption coefficient (mm^{-1})**	2.417	2.425
**F(000)**	1256	1256
**Crystal size (mm^3)**	0.310 × 0.240 × 0.170	0.310 × 0.240 × 0.170
**Reflections collected**	26561	13421
**Independent reflections**	6872	6732
**R(int)**	0.0268	0.0386
**Parameters**	334	334
**Goodness-of-fit on F^2**	1.019	1.035
**R_1 [I>2σ (I)]**	0.0371	0.0538
**wR_2 [I>2σ (I)]**	0.0949	0.1397
**Largest diff. peak and hole**	0.192 and -0.237	0.524 and -0.287

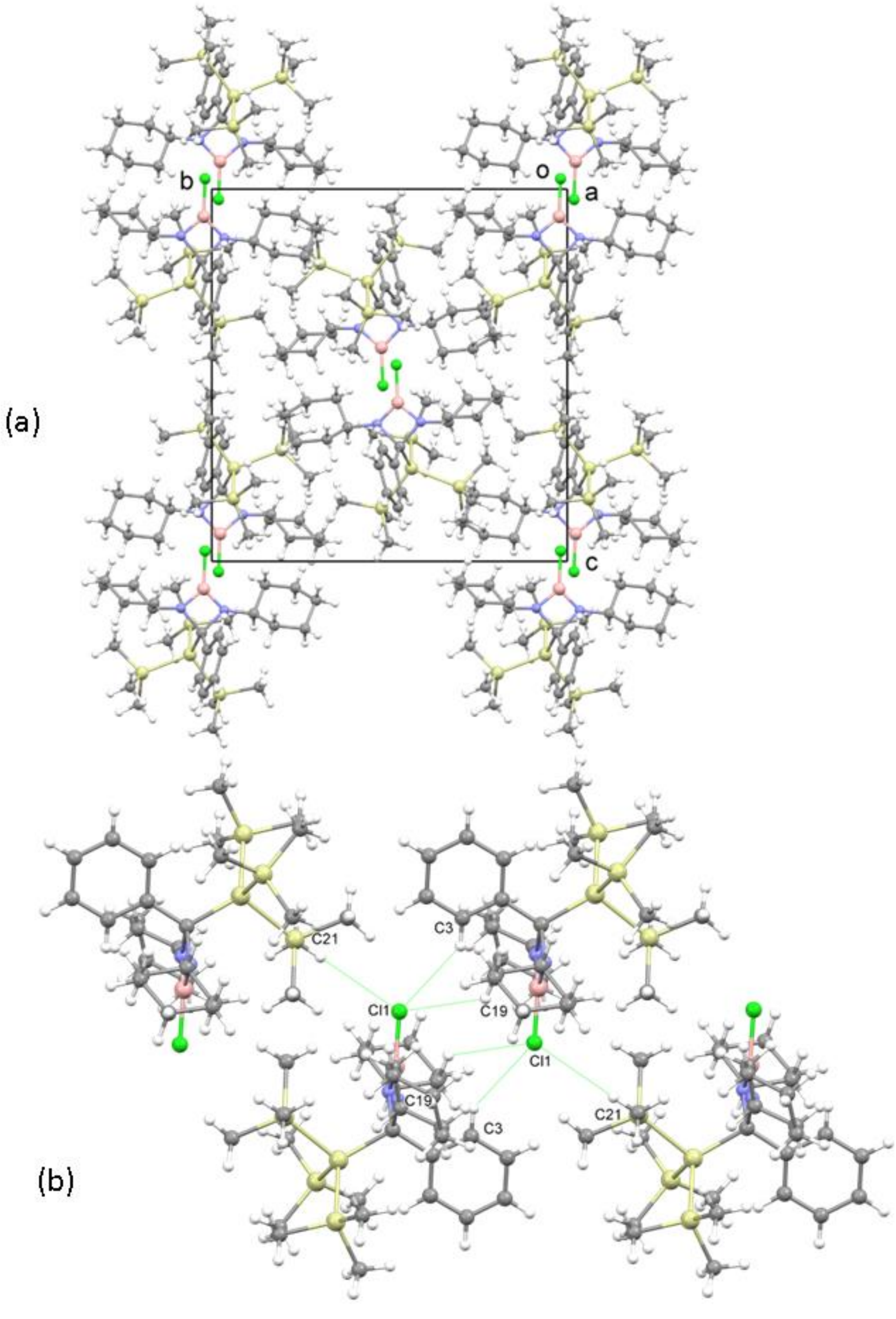

Figure S54. Crystal structure of HT form (a) crystal packing viewed down the a axis and (b) a segment of the structure showing some intermolecular contacts

(a)

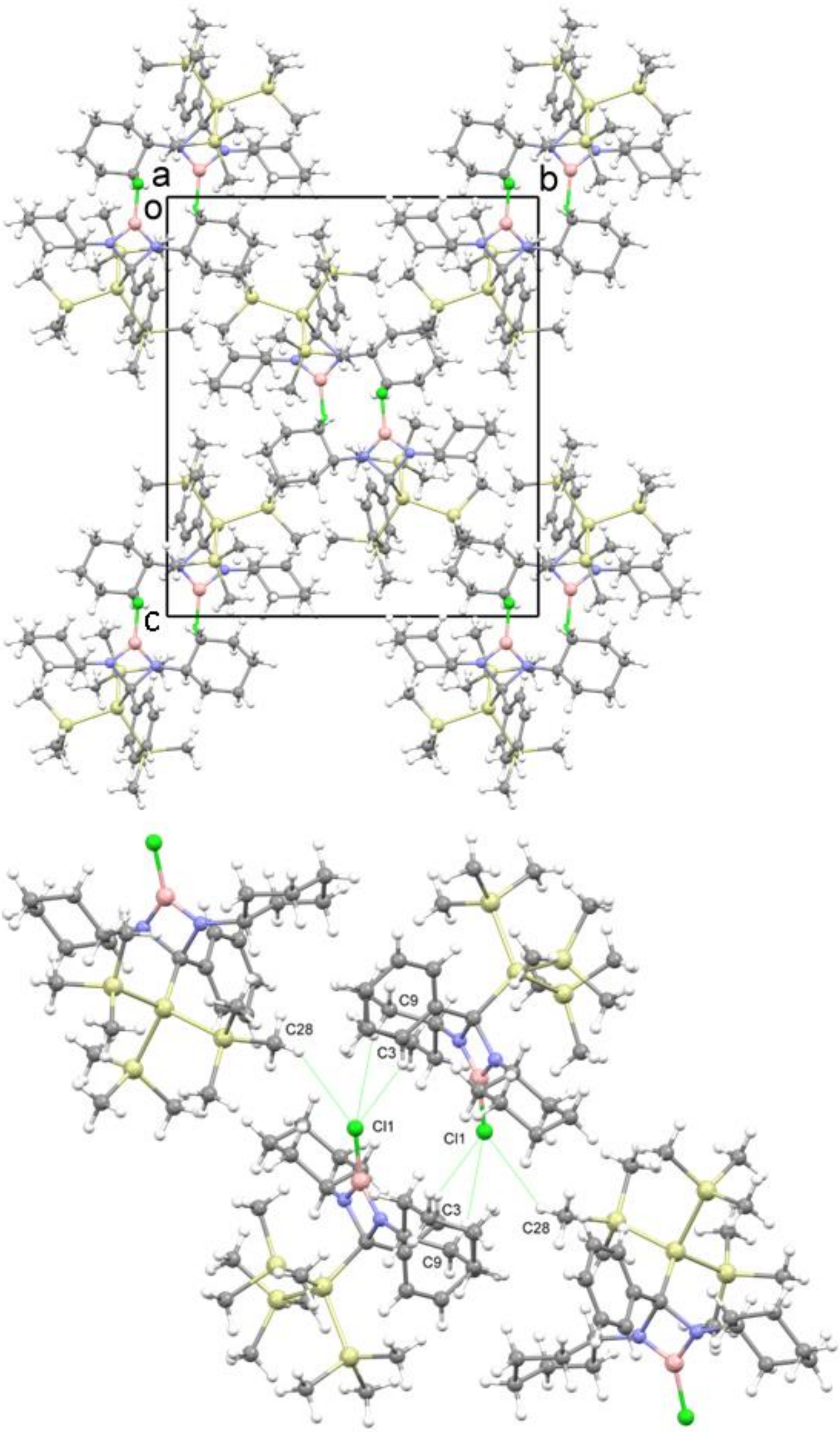

(b)

Figure S55. Crystal structure of LT form (a) crystal packing viewed down the a axis and (b) a segment of the structure showing some intermolecular contacts

The transformation from the HT and LT forms was observed to occur in situ for one crystal (C1) around 200 K during the recording of diffraction data. The unit cell switched from that of the HT form to that of the LT form after 36 minutes of data collection. For this crystal, a rarely observed induction period preceded the phase transformation. (A second crystal C2 from the sample batch did not require the induction period, as discussed below). Particularly notable is the increase in unit cell volume on the transformation of C1 from HT to LT at 200 K. The volume jumps by 1.5%. (Figure S56, red) with a corresponding decrease in the density (Table S3). Both the HT and LT forms have voids in the interstices between the molecules (Figure S57). For C1, these account for 1.7% of the unit cell volume for HT and 6% for LT. The transformation in phase is associated with a 250% increase in the void volume on cooling.

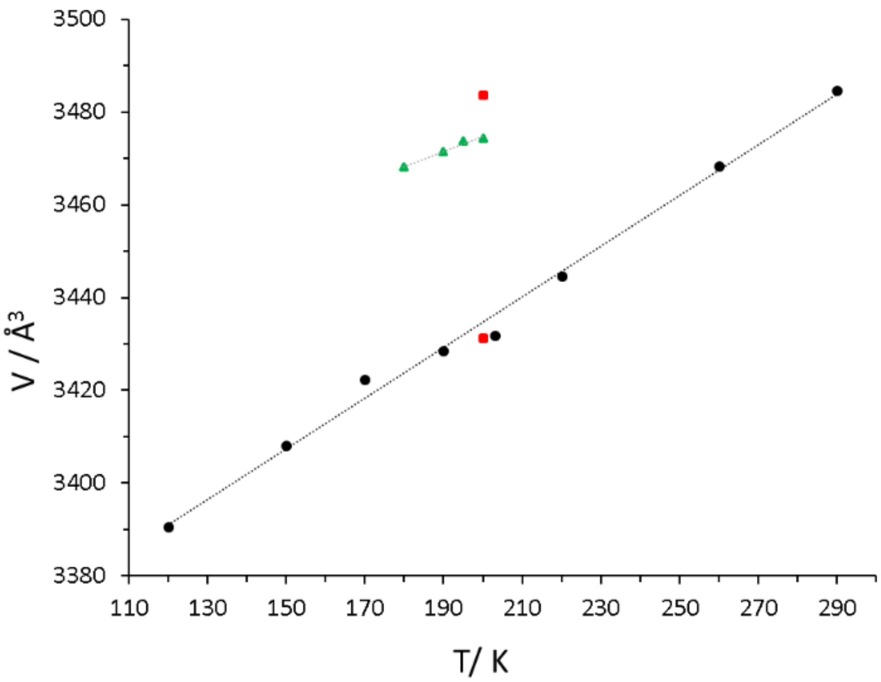

Figure S56. Unit cell volumes recorded on cooling the crystals from room temperature. C1 = red, C2 = green, C3 = black

Crystal C2 was cooled directly to 200 K and was observed to have changed to form LT immediately. Further cooling in steps from 200 K to 180 K led to a slight decrease in the unit cell volume (Figure S56, green), remaining significantly higher than corresponding values for the HT form. A slight increase in density from 1.104 to 1.106 Mg/m^3 accompanied the volume increase.

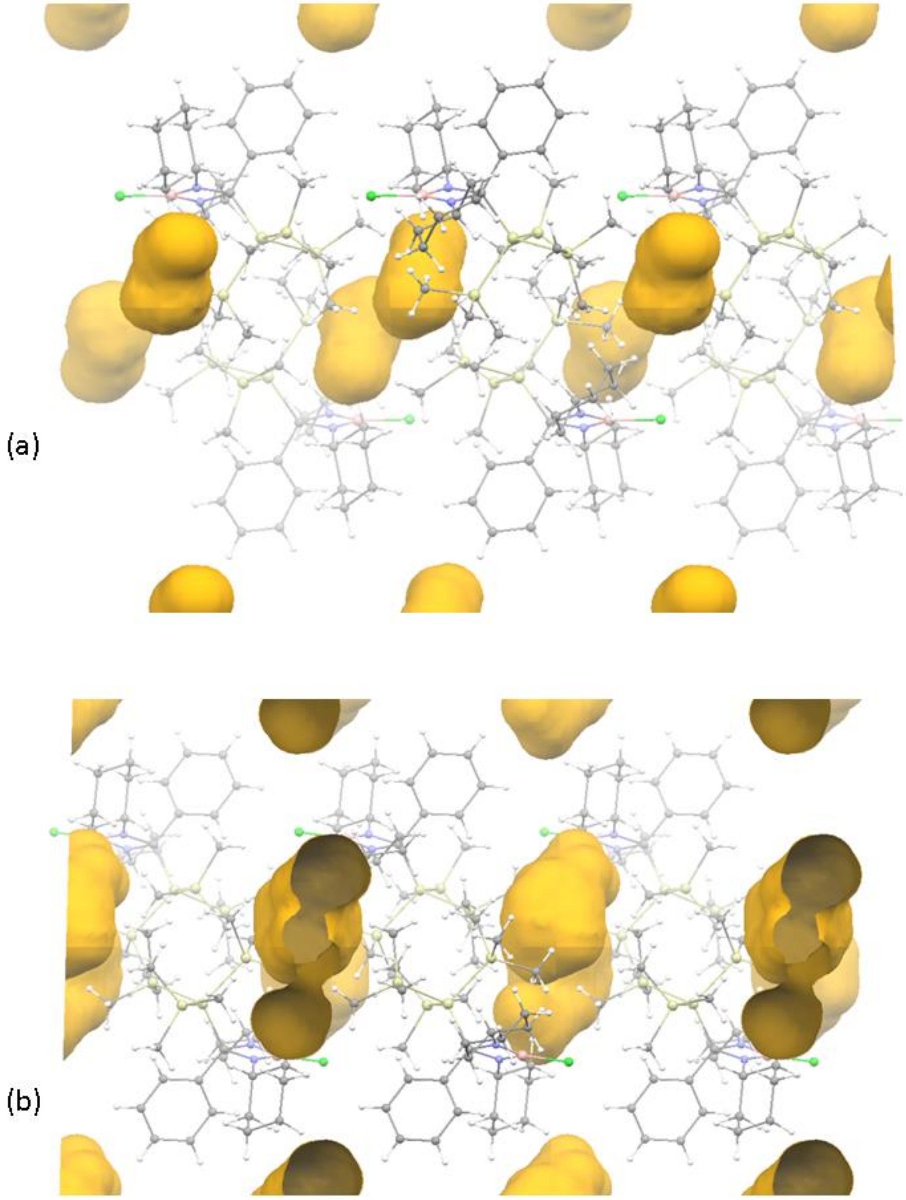

Figure S57. Voids in the crystals of the (a) HT and (b) LT forms

Table S2. Intermolecular C–H···Cl contact geometry (HT and LT from of crystals C2 and C3 respectively)

		**C···Cl distance (Å)**	**C–H···Cl angle (°)**
**HT**	C19–H19A···Cl1	3.943(3)	129.3
	C21–H21A···Cl1	4.140(3)	175.6
	C3–H3···Cl1	3.653(4)	127.1
**LT**	C3–H3···Cl1	3.754(3)	147.4
	C9–H9B···Cl1	3.967(3)	157.9
	C28–H28A···Cl1	3.631(3)	119.2

Table S3. Comparison of parameters for the transformation of crystal C1

	**Volume ($Å^3$)**	**Density (Mg/m^3)**	**Void ($Å^3$ /cell)**
**HT**	3431.3(3)	1.118	59
**LT**	3483.7(1)	1.101	207
**Change (%)**	1.53	-1.52	250

Crystal C3 did not transform on cooling. The HT form persisted from 290 K all the way down to 220 K. Cooling leads to a linear decrease in the unit cell volume with temperature. Notably, the rate of volume decrease for the HT form is about 1.7 times that for the LT form.

7. Computational Details

Gaussian 09, Revision D.01[S4] was used to fully optimize all the structures at the BP86 level[S5] of theory and the 6-31G(d) basis set[S6] was chosen for all atoms. Optimized geometries were verified by frequency computations as minima (zero imaginary frequencies) or transition structures (a single imaginary frequency) at the same level of theory. More accurate electronic energies were obtained by single point energy calculations at the SMD[S7]/M06-2X[S8]-D3/def2-TZVP[S9] level of theory considering the dispersive interactions.[S10] All thermodynamic data were calculated in the standard state (298.15 K and 1 atm). CYLview software was employed to present the 3D structures in the DFT section.[S11]

Cartesian coordinates and total energies for the calculated structures.

Compound **1**

E(BP86/6-31G(d)) = -1795.213672 au

H(BP86/6-31G(d)) = -1794.76206 au

G(BP86/6-31G(d)) = -1794.841853 au

E(SMD(Toluene)/M06-2X-D3/def2-TZVP//BP86/6-31G(d)) = -1795.147057 au

C	0.09432100	0.28580400	0.01322800
N	0.88912300	-0.76674000	-0.29212200
N	-1.08021600	-0.32482200	0.27785900
B	-0.35325800	-1.70851500	0.00116100
C	2.33420000	-0.98631400	-0.13815000
C	3.13521700	-0.59118000	-1.39972100
C	2.93241600	-0.33510900	1.12840600

H	2.42045100	-2.08449500	-0.01968900
C	4.62268400	-0.96475500	-1.24244200
H	3.04871200	0.50266300	-1.55409600
H	2.69358500	-1.08261400	-2.28582900
C	4.42150500	-0.70565400	1.27952900
H	2.83168900	0.76597200	1.05865300
H	2.35656600	-0.66421600	2.01255600
C	5.23109400	-0.33056800	0.02322100
H	5.18587000	-0.65298400	-2.14208000
H	4.71652600	-2.06823500	-1.18103600
H	4.84272400	-0.20958400	2.17413600
H	4.50681900	-1.79702200	1.45711600
H	6.28615800	-0.64245400	0.13960500
H	5.23841100	0.77312900	-0.09003700
C	-2.44006800	0.22055100	0.24464100
C	-2.97845500	0.39481600	-1.19630700
C	-3.37410600	-0.67686000	1.08152500
H	-2.40271700	1.22444000	0.71584300
C	-4.41782500	0.94675400	-1.18204800
H	-2.94929600	-0.58671100	-1.70477400
H	-2.30654200	1.07027700	-1.75948500
C	-4.81404500	-0.12805600	1.08602400
H	-3.36299000	-1.69575100	0.64777500

H	-2.97962900	-0.76601400	2.11006000
C	-5.35506900	0.05522300	-0.34480000
H	-4.79315400	1.03887500	-2.21852700
H	-4.41469700	1.97227400	-0.75745400
H	-5.46903600	-0.80594200	1.66440400
H	-4.83322900	0.84954700	1.61123200
H	-6.37338900	0.48707200	-0.31807800
H	-5.44370800	-0.93739500	-0.83059300
C	0.38482000	1.73588700	0.05842600
C	0.03711000	2.48324600	1.20557700
C	0.97552700	2.38761200	-1.04563200
C	0.29411900	3.86026400	1.25094100
H	-0.40838900	1.97208100	2.06569900
C	1.21974700	3.76743100	-0.99729900
H	1.21805600	1.81180500	-1.94438200
C	0.88368000	4.50431000	0.15027600
H	0.03472300	4.43154900	2.14882300
H	1.67134600	4.26871800	-1.86020700
H	1.08009900	5.58146000	0.18665600
Cl	-0.11344400	-2.75589000	1.50836700
Cl	-0.96963000	-2.66254300	-1.45452300

$KSiMe_3$

E(BP86/6-31G(d)) = -1009.175423 au

H(BP86/6-31G(d)) = -1009.057325 au

G(BP86/6-31G(d)) = -1009.103574 au

E(SMD(Toluene)/M06-2X-D3/def2-TZVP//BP86/6-31G(d)) = -1009.125794 au

Si	0.63646100	-0.00107600	-0.00007000
K	-2.60165900	-0.00167800	0.00028000
C	1.44824100	-1.45355400	-0.99277200
H	1.15030700	-2.43817700	-0.58638000
H	1.15061200	-1.43272400	-2.05784400
H	2.55515800	-1.39464100	-0.95215900
C	1.44546200	-0.13090200	1.75569300
H	1.14583600	0.71412900	2.40342800
H	1.14778700	-1.06307100	2.27130200
H	2.55246300	-0.12461800	1.68596300
C	1.43908300	1.58903800	-0.76341900
H	1.13733700	1.72897600	-1.81832100
H	1.13837300	2.49908300	-0.21126900
H	2.54647100	1.53048500	-0.73607200

KCl

E(BP86/6-31G(d)) = -1060.235382 au

H(BP86/6-31G(d)) = -1060.231002 au

G(BP86/6-31G(d)) = -1060.258162 au

E(SMD(Toluene)/M06-2X-D3/def2-TZVP//BP86/6-31G(d)) = -1060.209745 au

K	0.00000000	0.00000000	1.28866900

Cl	0.00000000	0.00000000	-1.44027700

TS_3A

E(BP86/6-31G(d)) = -2804.39229 au

H(BP86/6-31G(d)) = -2803.82344 au

G(BP86/6-31G(d)) = -2803.925922 au

E(SMD(Toluene)/M06-2X-D3/def2-TZVP//BP86/6-31G(d)) = -2804.282757 au

C	-0.07496800	0.04598500	-0.32025900
N	0.86299000	-0.96239600	-0.08122100
N	-1.16767400	-0.59783500	0.25644800
B	-0.33404700	-1.90649100	0.22850200
C	2.18243600	-1.19048400	-0.67727500
C	3.08385800	0.05812100	-0.59368400
C	2.85997300	-2.39208200	0.01590100
H	2.07201900	-1.46531300	-1.75043000
C	4.46721900	-0.21842700	-1.21369800
H	3.20509200	0.33806600	0.47285700
H	2.59871500	0.91825300	-1.08656300
C	4.24546300	-2.67376800	-0.59754800
H	2.97047500	-2.16698300	1.09756000
H	2.21095100	-3.28255000	-0.05883900
C	5.15159600	-1.42900800	-0.55123100
H	5.10230100	0.68276200	-1.12681000

H	4.34797300	-0.41356600	-2.29919400
H	4.72131100	-3.52153500	-0.07030600
H	4.11715500	-2.99478700	-1.65141000
H	6.12114100	-1.64006200	-1.04056100
H	5.37958800	-1.18320300	0.50678100
C	-2.57903300	-0.21357200	0.17096100
C	-3.25185600	-0.59309600	-1.17133700
C	-3.36333800	-0.81676400	1.35687700
H	-2.61067200	0.89076700	0.27372200
C	-4.72555600	-0.14309900	-1.19556000
H	-3.19061200	-1.68990400	-1.29943800
H	-2.69100900	-0.14200200	-2.00880500
C	-4.84468800	-0.38939800	1.32895100
H	-3.28651000	-1.92077100	1.31278300
H	-2.89620600	-0.50577900	2.31263200
C	-5.51374200	-0.73820500	-0.01404500
H	-5.18978400	-0.43498100	-2.15617400
H	-4.77453000	0.96478300	-1.14828300
H	-5.38636200	-0.86549000	2.16786200
H	-4.91050500	0.70611600	1.49383200
H	-6.55959600	-0.37712100	-0.02389900
H	-5.55776500	-1.84072500	-0.12428000
C	-0.16501500	0.97795600	-1.48119800

C	-0.82005500	2.22742900	-1.41192200
C	0.29004900	0.52795800	-2.74402200
C	-0.99132700	3.01256400	-2.55751500
H	-1.15629200	2.58986700	-0.43561000
C	0.13786900	1.32906200	-3.88716500
H	0.72975900	-0.46786600	-2.84149100
C	-0.49818600	2.57498300	-3.80043300
H	-1.49630600	3.98148000	-2.47564200
H	0.51107600	0.96522500	-4.85112800
H	-0.61147000	3.20208700	-4.69160600
Cl	-0.26898400	-2.89093300	1.87954600
Cl	-0.70397100	-3.11238000	-1.14866100
Si	0.84617200	2.77923100	1.51415500
C	-0.46749000	4.13372000	1.97011600
H	-1.11321100	4.39338900	1.11029800
H	-1.13487500	3.79491300	2.78519500
H	0.02382100	5.06972200	2.30484800
C	1.89603300	2.68059400	3.17639100
H	2.74934300	1.97435300	3.11317100
H	2.33363200	3.67194500	3.41193900
H	1.28486700	2.40309500	4.06185900
C	2.05377500	3.67636800	0.30779500
H	1.58550600	3.84968600	-0.67779700

H	2.35763800	4.66169900	0.71415500
H	2.97220900	3.08567700	0.13613000
K	0.48578400	-0.12163700	2.73103800

TS_3A′

E(BP86/6-31G(d)) = -2804.358695 au

H(BP86/6-31G(d)) = -2803.789718 au

G(BP86/6-31G(d)) = -2803.89194 au

E(SMD(Toluene)/M06-2X-D3/def2-TZVP//BP86/6-31G(d)) = -2804.247628 au

C	0.69438100	0.95771100	-0.03416900
N	1.31646600	-0.20315300	-0.34074400
N	-0.60633000	0.57993300	-0.08627400
B	-0.11694100	-0.88442800	-0.50114100
C	2.75161800	-0.48676000	-0.20430900
C	3.15492200	-0.99534600	1.20072800
C	3.24685300	-1.43383900	-1.31691100
H	3.26246800	0.48770100	-0.34980100
C	4.68002000	-1.20615300	1.28515100
H	2.62615000	-1.94294800	1.40425200
H	2.82242700	-0.26448900	1.96240100
C	4.77041800	-1.64756000	-1.21448600
H	2.72172900	-2.40146700	-1.21971800
H	2.97504100	-1.01472500	-2.30386000
C	5.17924800	-2.15744700	0.18072500

H	4.94787000	-1.59896500	2.28426000
H	5.19696000	-0.22877000	1.18425200
H	5.10107100	-2.35578000	-1.99781100
H	5.29353100	-0.68947500	-1.41800400
H	6.27827900	-2.27440900	0.24050400
H	4.74185400	-3.16323300	0.34281200
C	-1.78239700	1.36743800	-0.47509800
C	-2.28618300	2.31437300	0.63735700
C	-1.58849400	2.15454500	-1.79446900
H	-2.56002200	0.58828000	-0.63066300
C	-3.59465800	3.01453300	0.21910900
H	-1.51442200	3.08294900	0.83705200
H	-2.42815500	1.74383700	1.57269300
C	-2.89677900	2.85265400	-2.21708700
H	-0.79845200	2.91833900	-1.64702600
H	-1.23901700	1.46986100	-2.58679400
C	-3.42832700	3.77948900	-1.10774300
H	-3.92592300	3.70001000	1.02212400
H	-4.39507900	2.25503100	0.10565400
H	-2.73393900	3.42175000	-3.15222600
H	-3.66181800	2.08279200	-2.44769800
H	-4.38923200	4.23619200	-1.41245800
H	-2.71334300	4.61477100	-0.95945700

C	1.30158500	2.26111700	0.29722900
C	1.01990900	2.89776100	1.52838700
C	2.19245700	2.87986000	-0.61199800
C	1.61471000	4.12864800	1.83734500
H	0.35403900	2.40741600	2.24554400
C	2.77627900	4.11440700	-0.29868700
H	2.40100800	2.39787400	-1.57321700
C	2.49137600	4.74170700	0.92612500
H	1.39667600	4.60821200	2.79802600
H	3.45559300	4.58946800	-1.01511800
H	2.95254100	5.70490500	1.17054100
Cl	0.46590700	-2.92416200	0.20687000
Cl	-0.57088600	-1.22945600	-2.31310100
Si	-2.44480900	-1.58052500	1.33410300
C	-4.20920900	-0.79750800	1.58548500
H	-4.20916300	0.30368900	1.49684900
H	-4.92650400	-1.18021600	0.83359000
H	-4.61376600	-1.04615100	2.58822600
C	-2.87204300	-3.46509000	1.71747600
H	-1.98163600	-4.12102300	1.67069600
H	-3.26230800	-3.53596500	2.75251200
H	-3.67363600	-3.88551000	1.07107400
C	-1.46456400	-1.08285800	2.91631500

H	-1.26846300	0.00499700	2.94332200
H	-2.01461800	-1.34915300	3.84095900
H	-0.48633100	-1.59552000	2.93610400
K	-2.03223300	-3.61036000	-1.17395500

Compound **3-SiMe$_3$**

E(BP86/6-31G(d)) = -1744.199042 au

H(BP86/6-31G(d)) = -1743.632346 au

G(BP86/6-31G(d)) = -1743.722234 au

E(SMD(Toluene)/M06-2X-D3/def2-TZVP//BP86/6-31G(d)) = -1744.141536 au

C	-0.00765100	0.30381100	0.35311800
N	1.02764100	-0.81256900	0.16110500
N	-1.07796800	-0.65857200	-0.11439800
B	-0.06693300	-1.65652200	-0.26003100
C	2.39375600	-0.60992500	-0.34076000
C	3.28314700	0.14940300	0.66460800
C	3.03736500	-1.96634300	-0.70032500
H	2.35447400	0.00065200	-1.27201600
C	4.71630800	0.33610100	0.12982600
H	3.30952600	-0.42538600	1.61248300
H	2.83436900	1.13279000	0.89282600
C	4.47570300	-1.78931700	-1.22626900
H	3.04357100	-2.60763100	0.20366100
H	2.42231900	-2.48882500	-1.45344500

C	5.35919700	-1.01481600	-0.23207200
H	5.32972400	0.87164100	0.87886800
H	4.68884900	0.98162600	-0.77190000
H	4.91376700	-2.78161800	-1.44398700
H	4.44770000	-1.24170500	-2.19071800
H	6.37199600	-0.86294700	-0.65131000
H	5.48495900	-1.61709300	0.69112700
C	-2.52782000	-0.53953900	0.02963700
C	-3.08050400	0.72272000	-0.67225900
C	-3.22936300	-1.80237000	-0.51409000
H	-2.79134600	-0.46430300	1.10951100
C	-4.60940000	0.82806600	-0.51723900
H	-2.81344000	0.67113900	-1.74618700
H	-2.59021100	1.62879000	-0.27250100
C	-4.76096900	-1.69953500	-0.37149000
H	-2.95807000	-1.92730100	-1.58113900
H	-2.85537800	-2.69737100	0.01328400
C	-5.31624800	-0.43274100	-1.04791400
H	-4.97776000	1.73035100	-1.04047200
H	-4.86309700	0.96417100	0.55491500
H	-5.23329400	-2.60536000	-0.79569300
H	-5.02766100	-1.68374400	0.70538800
H	-6.40914800	-0.35939700	-0.89099700

H	-5.15784100	-0.50424100	-2.14348400
C	0.20413800	1.47960400	-0.60218300
C	0.11786800	1.27553000	-1.99848500
C	0.53711700	2.76952600	-0.14310300
C	0.34846500	2.32398000	-2.89824900
H	-0.14894100	0.28110800	-2.37196800
C	0.76457500	3.82581700	-1.04221700
H	0.62274200	2.96115300	0.93113300
C	0.67041600	3.60762700	-2.42337000
H	0.27313100	2.14043900	-3.97637100
H	1.01576400	4.82051200	-0.65657000
H	0.84578100	4.42947000	-3.12665200
Si	-0.25184900	0.65014000	2.28076000
C	-0.61352800	-1.02654300	3.09410300
H	-1.57809400	-1.46245000	2.78273700
H	0.18015400	-1.75337900	2.84923600
H	-0.63873000	-0.90879200	4.19309600
C	1.25328100	1.38015200	3.19617000
H	1.61939200	2.33795700	2.78906000
H	0.94600400	1.56546800	4.24319000
H	2.10124700	0.67659400	3.21959000
C	-1.70074000	1.85226000	2.59844700
H	-2.66942800	1.46572600	2.23834600

H	-1.79736600	2.02003800	3.68771500
H	-1.53902000	2.83734500	2.12627800
Cl	-0.14320400	-3.38162300	-0.72410400

Compound **3′-SiMe₃**

E(BP86/6-31G(d)) = -1744.200546 au

H(BP86/6-31G(d)) = -1743.633918 au

G(BP86/6-31G(d)) = -1743.728082 au

E(SMD(Toluene)/M06-2X-D3/def2-TZVP//BP86/6-31G(d)) = -1744.129871 au

C	-0.14158200	0.66948000	-0.02804700
N	1.07678600	0.08483600	0.02423200
N	-0.90362800	-0.35820200	-0.46611800
B	0.38946700	-1.28572400	-0.39169000
C	2.39049300	0.72673100	-0.01952500
C	3.46913300	-0.22682800	0.52953600
C	2.75388800	1.20360200	-1.44800700
H	2.34863300	1.62126300	0.63738500
C	4.86718600	0.42044600	0.48570900
H	3.46509400	-1.14955500	-0.08312700
H	3.20907900	-0.52496800	1.56244600
C	4.15061900	1.85333100	-1.48319700
H	2.71938900	0.33005500	-2.12580700
H	1.98380400	1.91549700	-1.80114800
C	5.22686700	0.89831200	-0.93395500

H	5.62222100	-0.29806900	0.85604300
H	4.89275200	1.28690600	1.17879900
H	4.39526400	2.16031900	-2.51747400
H	4.14215100	2.78160500	-0.87452300
H	6.21730000	1.39159900	-0.93330900
H	5.30979200	0.01897400	-1.60431700
C	-2.34772700	-0.60203800	-0.48706800
C	-3.12198600	-0.05257200	0.73162500
C	-2.99033700	-0.12312200	-1.81155700
H	-2.43698400	-1.70590800	-0.46902800
C	-4.60966500	-0.45505600	0.66041700
H	-3.04090100	1.05093400	0.75571700
H	-2.66297100	-0.42980400	1.66478600
C	-4.47667600	-0.52486100	-1.87860200
H	-2.90535500	0.98010100	-1.87518000
H	-2.42491400	-0.54228700	-2.66304000
C	-5.25946200	0.00117600	-0.66016900
H	-5.15388300	-0.03239200	1.52587400
H	-4.69335400	-1.55794000	0.74596900
H	-4.92550600	-0.15204800	-2.81843800
H	-4.55280600	-1.63085900	-1.91293100
H	-6.31243900	-0.33541600	-0.70499000
H	-5.28126000	1.10993200	-0.69110200

C	-0.48672100	2.05020600	0.38594600
C	-1.16307300	2.92898000	-0.48758400
C	-0.09349100	2.51044400	1.66274200
C	-1.45390800	4.23996200	-0.08361500
H	-1.43930200	2.58538400	-1.48962800
C	-0.39141600	3.82022500	2.06314500
H	0.42254300	1.82475200	2.34316700
C	-1.07229800	4.68687900	1.19207500
H	-1.97543200	4.91597800	-0.77000200
H	-0.09382000	4.16425000	3.05982600
H	-1.30295900	5.71069300	1.50656000
Si	0.35534200	-2.75407900	1.05749400
C	-1.06190700	-4.00277000	0.73893400
H	-2.06046600	-3.55345800	0.89040400
H	-1.02135600	-4.39474700	-0.29320000
H	-0.98212200	-4.86442500	1.42802700
C	1.97903400	-3.76089200	1.05388600
H	2.85010200	-3.14431800	1.33827200
H	1.91850100	-4.60468200	1.76638800
H	2.17682800	-4.17715600	0.05023300
C	0.11397500	-2.03141000	2.81578400
H	-0.81477100	-1.43638200	2.89374500
H	0.05457300	-2.83650600	3.57167700

H	0.95535500	-1.37203900	3.09740300
Cl	0.91631200	-1.97572600	-2.05821100

8. References

S1. (a) Hill, N. J.; Moore, J. A.; Findlater, M.; Cowley, A. H. Isolation of an Intermediate in the Insertion of a Carbodiimide into a Boron-Aryl Bond. *Chem. Commun.* **2005**, *43*, 5462–5464; (b) Dureen, M. A.; Stephan, D. W. Reactions of Boron Amidinates with CO_2 and CO and Other Small Molecules. *J. Am. Chem. Soc.* **2010**, *132* (38), 13559–13568; (c) Marschner, C.A. New and Easy Route to Polysilanylpotassium Compounds. *Eur. J. Inorg. Chem*. **1998**, 221-226.

S2. CrysAlisPro, Agilent Technologies, Version 1.171.37.33 (release 27-03–2014 CrysAlis 171.NET).

S3. SHELXL-2013, G.M. Sheldrick, University of Göttingen, Germany (**2013**).

S4. Frisch, M. J.; Trucks, G. W.; Schlegel, H. B.; Scuseria, G. E.; Robb, M. A.; Cheeseman, J. R.; Scalmani, G.; Barone, V.; Mennucci, B.; Petersson, G. A.; Nakatsuji, H.; Caricato, M.; Li, X.; Hratchian, H. P.; Izmaylov, A. F.; Bloino, J.; Zheng, G.; Sonnenberg, J. L.; Hada, M.; Ehara, M.; Toyota, K.; Fukuda, R.; Hasegawa, J.; Ishida, M.; Nakajima, T.; Honda, Y.; Kitao, O.; Nakai, H.; Vreven, T.; Montgomery, J. A.; Jr.; Peralta, J. E.; Ogliaro, F.; Bearpark, M.; Heyd, J. J.; Brothers, E.; Kudin, K. N.; Staroverov, V. N.; Kobayashi, R.; Normand, J.; Raghavachari, K.; Rendell, A.; Burant, J. C.; Iyengar, S. S.; Tomasi, J.; Cossi, M.; Rega, N.; Millam, J. M.; Klene, M.; Knox, J. E.; Cross, J. B.; Bakken, V.; Adamo, C.; Jaramillo, J.; Gomperts, R.; Stratmann, R. E.; Yazyev, O.; Austin, A. J.; Cammi, R.; Pomelli, C.; Ochterski, J. W.; Martin, R. L.; Morokuma, K.; Zakrzewski, V. G.; Voth, G. A.; Salvador, P.; Dannenberg, J. J.; Dapprich, S.; Daniels, A. D.; Farkas, O.; Foresman, J. B.; Ortiz, J. V.; Cioslowski, J.; Fox, D. J. Gaussian 09, revision D.01, Gaussian Inc.: Wallingford, CT, **2009**.

S5. (a) Becke, A. D. Density-functional exchange-energy approximation with correct asymptotic behavior. *Phys. Rev. A.* **1988**, *38*, 3098-3100. (b) Perdew, J. P. Density-functional approximation

for the correlation energy of the inhomogeneous electron gas. *Phys. Rev. B.* **1986**, *33*, 8822−8824.

S6. Hariharan, P. C.; and Pople, J. A. The influence of polarization functions on molecular orbital hydrogenation energies. *Theor. Chim. Acta J. A.* **1973**, *28*, 213−222.

S7. Marenich, A. V.; Cramer, C. J.; Truhlar, D. G. Universal solvation model based on solute electron density and on a continuum model of the solvent defined by the bulk dielectric constant and atomic surface tensions. *J. Phys. Chem. B.* **2009**, *113*, 6378−6396.

S8. Zhao, Y.; and Truhlar, D. G. Density functionals with broad applicability in chemistry. *Acc. Chem. Res.* **2008**, *41*, 157−167.

S9. Weigend, F.; Furche, F.; Ahlrichs, R. Gaussian basis sets of quadruple zeta valence quality for atoms H–Kr. *J. Chem. Phys.* **2003**, *119*, 12753–12762.

S10. Grimme, S.; Antony, J.; Ehrlich, S.; and Krieg, H. A consistent and accurate ab initio parametrization of density functional dispersion correction (DFT-D) for the 94 elements H-Pu. *J. Chem. Phys.* **2010**, *132*, 15104–15123.

S11. C. Y. Legault, CYLview, 1.0b, Université de Sherbrooke, **2009** (http://www.cylview.org).
